# Extraordinary diversity of the CD28/CTLA4 family across jawed vertebrates

**DOI:** 10.3389/fimmu.2024.1501934

**Published:** 2024-11-13

**Authors:** Sylvie M. A. Quiniou, Thomas Clark, Eva Bengtén, Jonathan P. Rast, Yuko Ohta, Martin Flajnik, Pierre Boudinot

**Affiliations:** ^1^ US Department of Agriculture (USDA)-ARS-WARU, Stoneville, MS, United States; ^2^ The Roslin Institute and Royal (Dick) School of Veterinary Studies, University of Edinburgh, Edinburgh, United Kingdom; ^3^ Department of Cell and Molecular Biology, University of Mississippi Medical Center, Jackson, MS, United States; ^4^ Center for Immunology and Microbial Research, University of Mississippi Medical Center, Jackson, MS, United States; ^5^ Emory University School of Medicine, Pathology & Laboratory Medicine, Atlanta, GA, United States; ^6^ Department of Microbiology and Immunology, University of Maryland School of Medicine, Baltimore, MD, United States; ^7^ Université Paris-Saclay, INRAE, UVSQ, VIM, Jouy−en−Josas, France

**Keywords:** CD28, CTLA4, PD1, CD28H, ICOS, Immune checkpoints, costimulatory receptors

## Abstract

Members of the CD28 family are critical for the control of immune cell activation. While CD28 and CTLA4 were previously identified in teleost fish, most members of the CD28 family have been described only in tetrapods. Using a comparative genomics approach, we found (co)orthologs of all members of the CD28 family both in Chondrichthyes and basal Osteichthyes groups, but not in Agnathans. Four additional members of the family were identified, which were present in both Chondrichthyes and Osteichthyes, some even in the tetrapod lineage but all of them absent in human. Herein, we extend the composition of the jawed vertebrate CD28 family to nine members: CD28, CTLA4, ICOS, CD28H, CD28HL1, CD28HL2, CD28HL3, CD28X and PD-1. Each of these genes had a single extracellular IgSF V domain, and conserved motifs in the V and the cytoplasmic domain. While a genomic cluster of three consecutive genes like CD28/CTLA4/ICOS was conserved across jawed vertebrates except in teleosts, the other members of the CD28 family were located on multiple chromosomes. Our findings show that these co-stimulatory/co-inhibitory receptors likely arose in early jawed vertebrates, and diversified when the Ig/TCR/MHC-based adaptive immunity emerged, heralding the advent of complex regulatory networks controlling lymphocyte activation.

## Introduction

Early studies of TCR-mediated activation of T cells revealed that TCR engagement alone was not sufficient for full activation and led to T cell anergy, as predicted by Bretscher and Cohn (1, 2). Thus, a second “co-stimulatory signal” preventing the induction of T cell anergy had to be delivered by activated antigen (Ag) presenting cells (3, 4). CD28 was identified as a key co-stimulatory receptor when treatment with anti-CD28 monoclonal Ab was shown to provide a second signal which, in combination with TCR engagement, led to full activation of human and mouse T cells (4–6). As a counterbalance, the co-inhibitory receptor CTLA4 is then induced during the cell proliferation, and by binding to B7-1 and B7-2 with a higher affinity than CD28 prevents T cell activation in human and mice (7, 8).

Both CD28 and CTLA-4 are members of the CD28 family, defined by a domain structure with an extracellular Immunoglobulin (Ig) V-set domain with common structural motifs, a transmembrane region, and a cytoplasmic domain with tyrosine signaling motifs (9). Following the discovery of these two co-receptors, three additional co-stimulatory and co-inhibitory receptors belonging to the CD28 family were identified: inducible co-stimulator (ICOS) (10), Programmed cell death-1 (PD1, aka PDCD1) (11) and CD28-homolog (CD28H), aka transmembrane and immunoglobulin domain containing 2 (TMIGD2) or immunoglobulin-containing and proline-rich receptor-1 (IGPR1)) (12, 13). ICOS, a co-stimulatory receptor expressed by human and mouse CD4^+^ and CD8^+^ T cells, induces the production of IL4, IL10 and IL21, and binds its ligand ICOSL expressed by B cells, macrophages, and dendritic cells (14). PD1, a co-inhibitory receptor with cytoplasmic immunoreceptor tyrosine-based inhibitory motif (ITIM)/immunoreceptor tyrosine-based switch motif (ITSM) motifs, is quickly upregulated by TCR or cytokine (IFNa, IL6, IL12) signaling (15) to dampen T cell activation (16, 17) in human and mice. CD28H, the most recently discovered member of the family, is another positive co-stimulatory receptor expressed by human T cells and Natural Killer (NK) cells (18). CD28 family co-inhibitory receptors are key “immune checkpoints” targeted successfully for cancer immunotherapy (19), which resulted in awarding of the Nobel Prize to James P. Allison and Tasuku Honjo for their discovery that by constraining such negative immune regulation ongoing immune responses can be perpetuated.

Orthologs of CD28 and CTLA4 were identified in rainbow trout, a teleost fish (20). They have a typical structure and conserved motifs such as a V domain CDR3 with three consecutive prolines, and the Gly-x-Gly in the CTLA4 IgSF G-strand, which is critical for the high-affinity binding of CTLA4 to B7 molecules competing with the lower affinity CD28 (21). The tyrosine signaling motif was also generally conserved in the CD28 cytoplasmic domain but not in CTLA4, raising questions concerning the mechanisms of action of this receptor. Importantly, signaling motifs vary across fish families as cyprinid CD28 completely lack tyrosine motifs (22). Survey of teleost fish genome and transcriptome datasets led to the discovery of orthologs of B7 and B7-homologs (B7H) (23), including B7H1/B7DC (PD-1 ligands (PDL)1/2), B7H3, B7H4, B7H5 and B7H7/HERV-H LTR-associating protein 2 (HHLA2) (22, 24). However, orthologs of CD28 family members PD1 (binding B7H1/B7DC) or CD28H (binding B7H7/HHLA2) were not found. Neither were ICOS/ICOSL genes detected. These observations raised questions about the pathways in which teleost CD28 members were involved, as well as about the origin and evolution of this family of co-stimulatory receptors and their immune functions.

In this study, we investigated whether CD28 family members were present in the genomes of Agnathans, Chondrichthyes, and basal Osteichthyes taxa, in comparison to teleosts and tetrapods. Our analysis was based on domain structure, key motifs such as those found in CD28 (25), and conserved syntenies. We failed to identify CD28-related genes in lamprey or hagfish, but orthologs of CD28, CTLA4, ICOS, PD1 and CD28H were detected in basal branches of jawed vertebrates, showing that all members of the CD28 family were present much earlier than previously demonstrated. We also discovered a set of new homologs of CD28H, which are absent in human and have been lost at different stages of tetrapod evolution. Altogether, these findings raise interesting questions about the evolution of the control of adaptive immunity in all vertebrates.

## Materials and methods

### Protein sequence identification

The identification of a complete array of the CD28 family co-stimulatory molecules in basal branch of vertebrates and other species of interest was conducted using a combination of several strategies. Human and rainbow trout protein sequences were used to conduct searches using NCBI TBLASTN or BLASTP (with default parameters) against genome assemblies, RNAs and expressed sequence tags (EST) sequences available in NCBI, and subsequently found sequences in other species were also used. For the sea lamprey (*Petromyzon marinus*), kPetMar1.pri annotation models (Annotation Release 100) were also searched using HMMSEARCH (v. 3.3.0, with a low stringency domain Evalue of 100 [-E 100]) and immunoglobulin domain PFAM models (http://pfam-legacy.xfam.org/; V-set, V-set_2, ig, Ig_2, Ig_3, Ig-4, I-set, Receptor_2B4, Big_3_4, C1-set, C2-set, C2-set_2). Individual hits were then inspected to identify genes encoding proteins with structural organization showing similarity to CD28-family members. Automatic annotations of the different genomes were also searched using gene names. Genes of interest were also identified based on their domain structure and their proximity to conserved genes in the respective synteny environment of each gene model then verified by reverse TBLASTN against mammals or other tetrapods. Genes were considered as members of the CD28 family when (1) they encoded proteins with the conserved IgV-TM-IC domain structure and with conserved motifs in the CDR3 neighborhood and in the cytoplasmic region and (2) were located in genomic regions with conserved synteny markers. Protein sequences for all genes analyzed here were collected from GenBank. The species and genome assembly used are available in **Supplementary Data Sheet 1**. Accession numbers for all sequences are found in **Supplementary Data Sheets 2, 4**.

### Sequence analysis

The protein structure analysis (Ig domains, TM domain, leader peptide) was performed using SMART (http://smart.embl.de/). Protein domains were also analyzed using InterProScan (https://www.ebi.ac.uk/jdispatcher/pfa/iprscan5). Protein sequence alignments and computing of sequence similarity scores were conducted using Clustal Omega (https://www.ebi.ac.uk/jdispatcher/msa/clustalo).

### Synteny analysis

Synteny for each gene of interest was analyzed from the NCBI genome data viewer or Ensembl resources. The respective genomic locations were extracted in the NCBI genome data viewer, and all coordinates and locations are given according to genome assemblies available at the NCBI. Neighboring gene identity was verified by blast and domain analysis when relevant.

### Phylogenetic trees

Phylogenetic reconstruction was performed using the Maximum Likelihood method and JTT matrix-based model. The optimal tree is shown. The percentage of replicate trees in which the associated taxa clustered together in the bootstrap test (100 replicates) are shown next to the branches. Evolutionary analyses were conducted in MEGA X (26).

## Results

### Orthologs of tetrapod CD28, CTLA4 and ICOS are present in the genomes of basal jawed vertebrates

**Figure 1:**
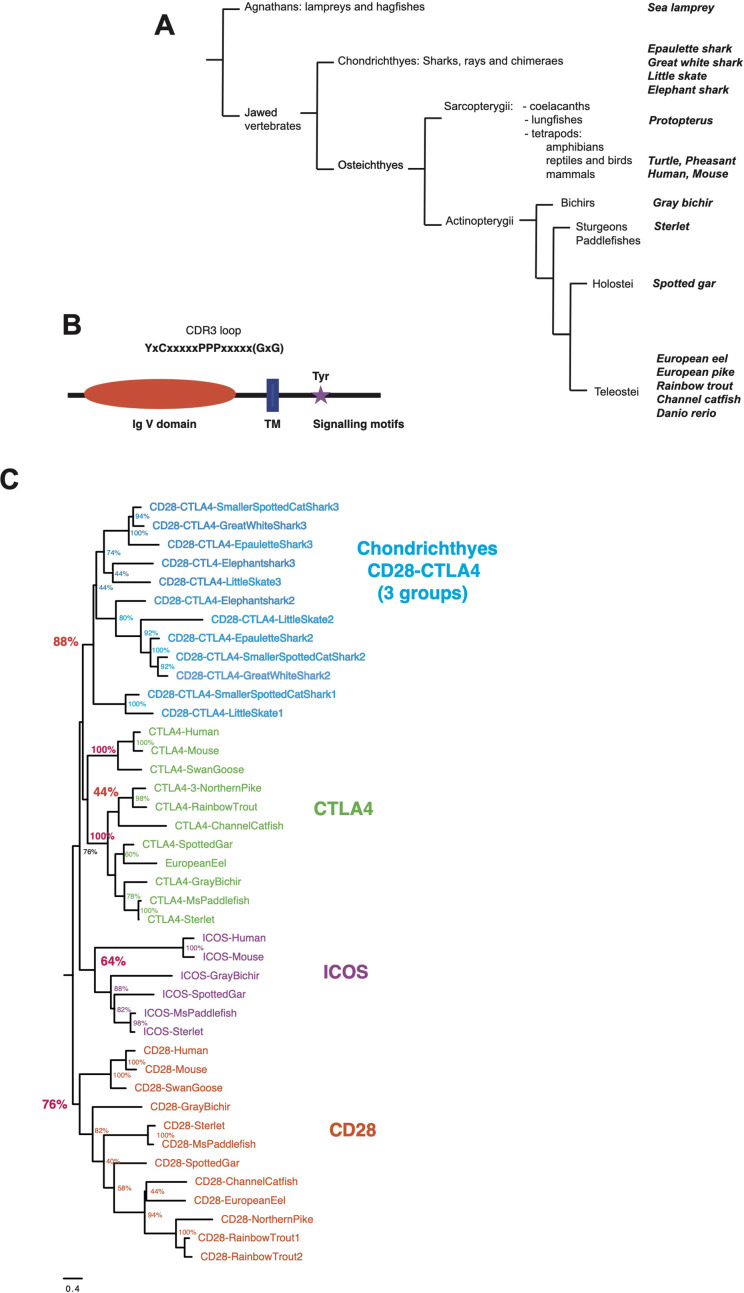
**(A)** Schematic phylogenetic tree of the taxonomic groups considered in this study. Taxonomy as in Helfman et al. (2010) for fish and in NCBI. **(B)** Domains and motifs generally conserved in members of the CD28 family. The GxG motif present in CTLA4 G-strand is not present in all members and is indicated with “()”. **(C)** Evolutionary analysis of CD28, CTLA4, ICOS and chondrichthyan CD8/CTLA4 homologs. The evolutionary history was inferred by using the Maximum Likelihood method and JTT matrix-based model. The tree with the highest log likelihood (-15105.34) is shown. The percentage of trees in which the associated taxa clustered together is shown next to the branches. Initial tree(s) for the heuristic search were obtained by applying the BioNJ method to a matrix of pairwise distances estimated using a JTT model. A discrete Gamma distribution was used to model evolutionary rate differences among sites. The number at the end of chondrichthyan genes refers to their position in the genomic cassette of three CD28/CTLA4 genes.

To revisit the repertoire of CD28 and related receptors across vertebrates, we first performed tblastn analysis using sequences from human, rainbow trout, and other fish species on high-quality genomes and EST databases selected from bony fish and Chondrichthyes. Sequences related to CD28 and CTLA4 were identified in basal branches of the vertebrate phylum, *i.e,.* bichirs, sturgeons, gar, and early sarcopterygians (**Figure 1A**). Candidates were selected on the basis of detection by blast analysis combined with domain structure and presence of canonical motifs (**Figure 1B**). The tree produced by maximum likelihood phylogenetic reconstruction was mostly consistent with the taxonomic tree, and identified 4 main clusters of sequences: (1) a CD28 cluster; (2) a CTLA4 cluster, with mammalian and trout receptors as well as other bony fish sequences but no chondrichthyan-specific orthologs; (3) an ICOS cluster with mammalian and bird references, and sequences from gar and sterlet, but no hit from teleosts, chondrichthyans, or basal Sarcopterygians (*e*.*g*., Coelacanth, Protopterus); and (4) a cluster of CD28/CTLA4 related sequences exclusively found in Chondrichthyes (**Figure 1C**). These data suggested that the specific features of CD28 and CTLA4 were acquired after the divergence of Chondrichthyes and Osteichthyes. They also revealed that ICOS is an ancient receptor.

To further confirm identification of these sequences, we then focused on canonical conserved motifs (24) (**Supplementary Data Sheet 3–A**).

#### CD28

The PPP motif located in the CDR3 loop of the V domain was conserved in all CD28-like sequences from mammals, bichir, gar, sterlet, and bony fish (**Supplementary Data Sheets 2**, **3–A**). As in human and rainbow trout, this motif was associated with a G-strand lacking the GxG motif, typically conserved in CTLA4 sequences (24). There were some exceptions, as in sequences from chondrosteans (e.g., sturgeons and paddlefish), which clustered with other CD28 sequences with high bootstrap values but contained a typical GxG motif in the positions where it is found in CTLA4, and in Protopterus (**Supplementary Data Sheet 3–A**). The dimerization motif YxxxxT, which is conserved in tetrapod CD28 transmembrane region (27), was also present in most of these proteins, indicating that they likely function as dimers. This motif, which is similar to the transmembrane CD3ζ dimerization motif, has been shown to be critical for the expression of stable CD28 dimers (27). The signaling motif in the cytoplasmic tail of CD28 proteins showed a well-conserved consensus [D/E/V/I]-Y-M-[N/D]-[M/I/V/T] containing a putative binding site for Phosphoinositide 3-kinase (PI3K) (YxxM) and Growth factor receptor bound protein 2 (GRB2)/Grb2-related adaptor downstream of Shc (GADS) (YxNx), except in cyprinids as previously reported (22). Hence, typical CD28 genes are present in basal groups of Osteichthyes. In contrast, the important proline signaling motifs PRRP and PYAP of human CD28, which were not conserved in teleosts (24), were also not present in Chondrichthyes or basal fish groups (**Supplementary Data Sheets 2** and **3-A**).

#### CTLA4

CTLA4-related sequences also clustered together in phylogenetic reconstruction, although mammalian and fish sequences formed distinct distant branches. A [M/L/I]-[Y/F]-P-P-P-Y motif was present in all these sequences associated to a GxG motif found in a typical Ig or TCR V-domain G strand (**Supplementary Data Sheets 2**, **3-A**). This di-glycine motif (G-x-G), which implies the presence of a β bulge (28), has been reported previously as a conserved feature in CTLA4 from fish to mammals (20, 24), and as mentioned above is important for the high-affinity binding of CTLA4 to B7, causing disruption of CD28-B7 interactions (21). Another conserved feature of CTLA4 sequence was the V-[N/D]-L motif in the E strand of the V domain; in humans, a glycan attached to this asparagine is critical for the structural integrity of the domain (28) (**Supplementary Data Sheet 3**). The YxxxxT dimerization motif was conserved between CD28 and CTLA4 in human and mouse but not found in fish CTLA4 sequences. Cytoplasmic signaling motifs were also less conserved. The Y-based motif corresponding to the human SHP-2 binding site YVKM was missing in most teleost CTLA4 sequences. This motif may have been lost during the early evolution of this group since it is present in sterlet and in eel. The JAK2-binding PTEP motif conserved in human and mouse (29) was not found in all tetrapods and also absent in fish. The P-Y-x-x-P motif located at the C-terminus of human CTLA4, which has been shown to be involved in CTLA4 signaling (29), may be more ancient. This motif was either absent or had degenerated in reptiles and amphibians (**Supplementary Data Sheet 3-A**), but a Y-based motif was present at this position in the gray bichir (Y-V-M-F), in Chondrosteans (Y-V-I-L), and it was also well conserved in spotted gar and teleosts (Y-[E,G,R]-N-F) (**Supplementary Data Sheets 2**, **3-A**). Importantly, this teleost YxNF motif supports clathrin-mediated endocytosis, although with significantly lower efficiency than the mammalian YVKM motif (30). Altogether, these observations suggest that the CTLA4 signaling has evolved in multiple ways across tetrapods but supports the idea of potential similar functions.

#### ICOS

ICOS homologs have been reported in mammals and reptiles (birds; turtles) but were never found in teleost fish (24) nor in Chondrichthyes. Interestingly, we found sequences with ICOS-specific features in basal groups of bony fish (spotted gar and sturgeons) where they consistently clustered with ICOS from human and reptiles in phylogenetic trees (**Figure 1C**). The P-[P/A/L]-P motif in the CDR3 loop was associated with a G-strand GxG motif in turtle, gar and sterlet but not mouse and human, and was less conserved than in CD28 or CTLA4. Interestingly, while the YxxxxT dimerization motif found in human CD28 and CTLA4 was not conserved in mammalian or reptilian ICOS, we found it in gar, sterlet and paddlefish ICOS-like sequences, suggesting it may function as a dimer in these species. The cytoplasmic signaling motif E-Y-M-[D/P/F]-M was fully conserved across all these sequences from fish to human (**Supplementary Data Sheets 2**, **3-A**).

#### Chondrichthyan CD28/CTLA4 related sequences

In Chondrichthyes, two or three sequences similar to CD28 and CTLA4 were found per genome. These sequences clustered in a separate group in phylogenetic trees. This group comprised three well-supported branches with representative(s) in each species, respectively with genes from the first, second and third position in the genomic cluster (**Figure 1C**). However, we failed to identify hints of robust orthology between these three respective sets and CD28, CTLA4 and ICOS sequences. The P-[P/L]-P motif in the V domain CDR3 loop was followed by a GxG as described for CTLA4 in these sequences. A YxxxxT dimerization motif was generally present in the TM, as in human CD28 and CTLA4 (**Supplementary Data Sheet 2**). A Y-based signaling motif was found in most sequences at the same location as in CD28 or CTLA4; of note, a EYxNM motif is generally present in sequences located in the third position in the genomic cluster of three CD28/CTLA4 co-orthologs (**Supplementary Data Sheets 2**, **3-A**).

**Figure 2:**
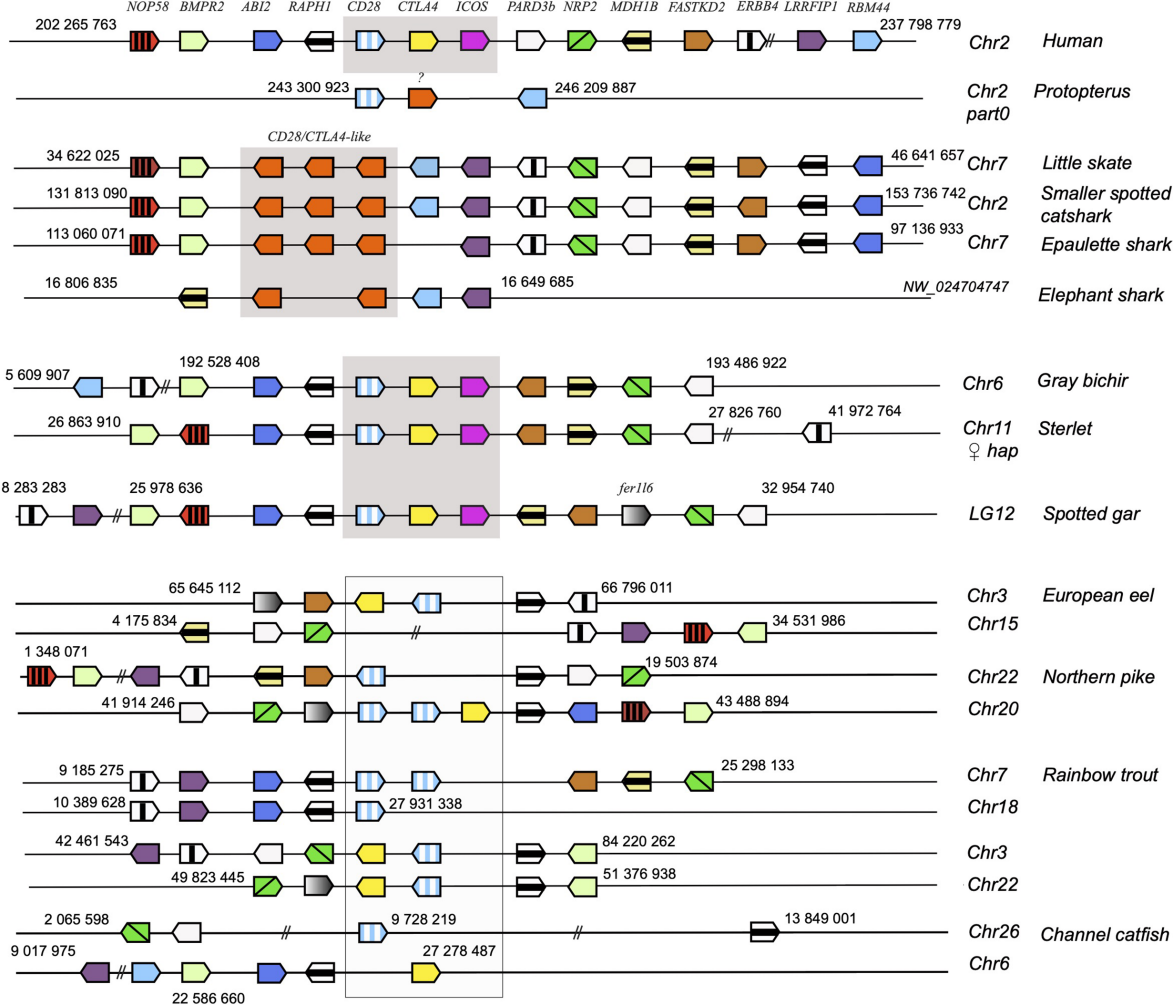
Conserved synteny blocks with CD28 (striped blue and white), CTLA4 (yellow), ICOS (pink) and chondrichthyan CD28/CTLA4 homologs (orange). Consecutive gene groups are boxed in grey. Only conserved markers are represented in the regions of interest, of which start and end location are indicated in the reference NCBI chromosome/LG of each species.

To further confirm the evolutionary affinities of these sequences, we analyzed their syntenic regions for conserved genetic markers. In mammals and birds, *cd28, ctla4* and *icos* are closely linked in the context of a conserved group marker genes (**Figure 2**). Strikingly, this configuration was conserved in gar and sterlet. Furthermore, the CD28/CTLA4-related sequences found in Chondrichthyes also formed a genomic cluster of three genes in little skate, great white shark and epaulette shark (**Figure 2**). The markers flanking human CD28/CTLA4/ICOS and paralogs were also syntenic with CD28 and CTLA4 in different chromosomes in teleosts, but ICOS was apparently lost. In the European eel, a species belonging to the monophyletic sister group to all other teleosts in the taxon Elopomorpha, both CD28 and CTLA4 were retained on Chr3. No gene of the CD28 family were present in paralogous region located on Chr15. European pike CD28 and CTLA4 were found in the two copies of the conserved synteny group produced by the teleost-specific whole genome duplication (WGD) and located on Chr 20 and 22 (**Figure 2**). In salmonids such as rainbow trout, CD28 and/or CTLA4 were associated with each of the 4 copies of this conserved region, retained after teleost and salmonid-specific WGDs (**Figure 2**). This conserved genomic context confirms cluster of three consecutive genes encoding CD28-related receptors was the primordial genomic configuration of the CD28-related loci, which is conserved in both Chondrichthyes and Osteichthyes. Overall, our data indicate that CD28 and CTLA4 emerged with their distinctive features after the divergence between Chondrichthyes and Osteichthyes, as well as receptors with some ICOS-like features. The related sequences found in Chondrichthyes could not be classified as orthologs of CD28, CTLA4 or ICOS, and appear to have evolved independently in this group.

### CD28H is an ancient, highly conserved member of the CD28 family

**Figure 3:**
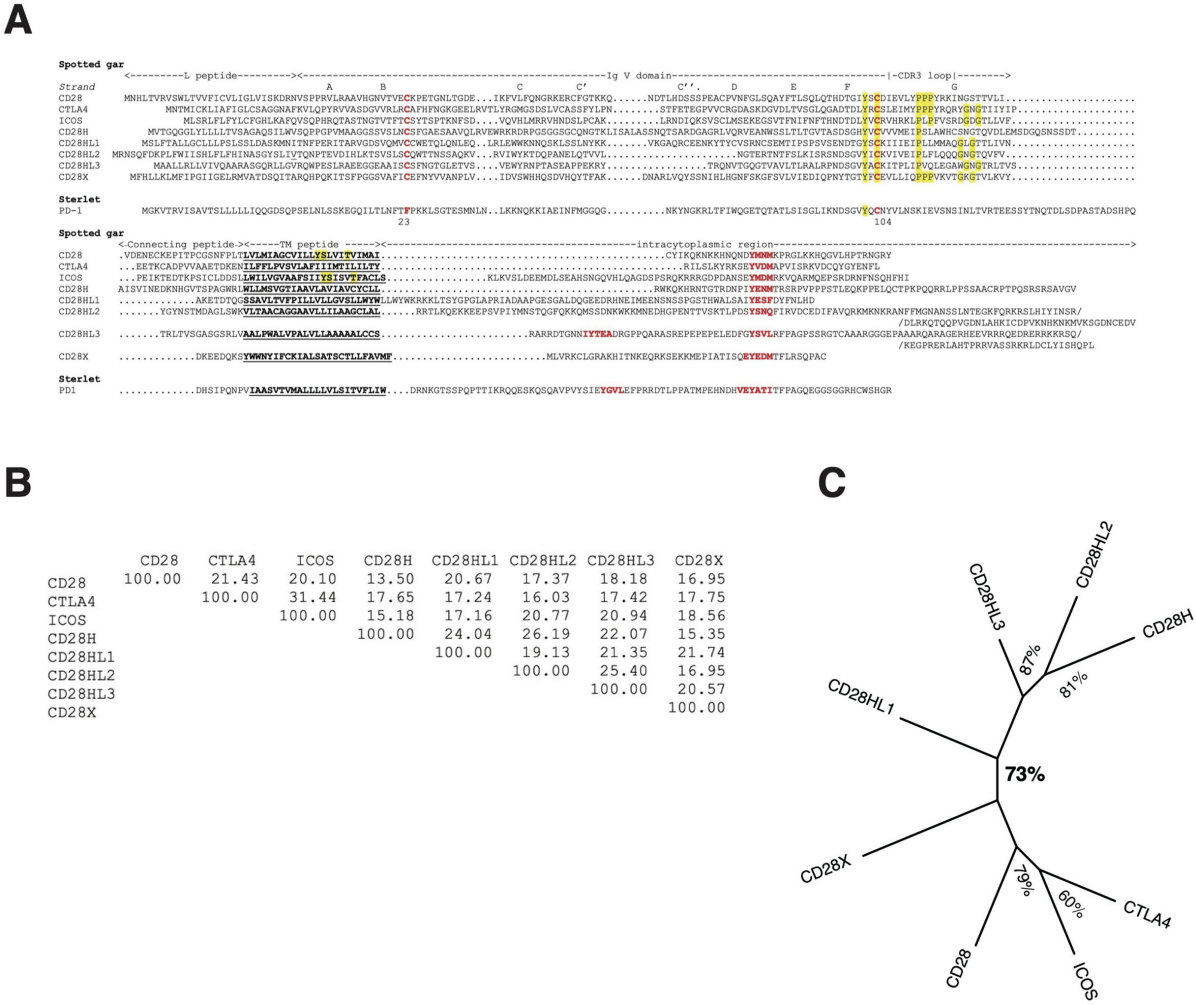
The repertoire of CD28 family members. **(A)** Multiple alignment of the CD28 family member AA sequences of the species with the widest repertoire, the spotted gar. A PD-1 putative homolog was identified in the spotted gar but the protein model lacked key motifs and might be partial, hence the sterlet sequence was included in the figure for a better representativity. Similarity matrix **(B)** and unrooted ML phylogenetic tree **(C)** of Spotted gar sequences showing clustering of CD28H with CD28HL sequences.

CH28H (aka TMIG2) is another member of the CD28 family, which has been described in human as a positive T cell co-stimulatory receptor binding B7H7 (31). We identified closely related homologs in several chondrichthyans, as well as in ancient bony fish groups as shown for sterlet, spotted gar and European eel in **Supplementary Data Sheet S3-B** (see also **Figure 3**). Using these sequences for blast analysis, we then found similar sequences in teleost species including Northern pike, Atlantic salmon, and three-spined stickleback. CD28H appears to be lacking in a number of species including great white shark in Chondrichthyes and the gray bichir. While *cd28h* was present in birds and turtles, it seems to be missing in squamata. We also failed to find it in amphibians, and in basal sarcopterygians.

An E-I-P motif in the CDR3 loop of the V domain was highly conserved in CD28H from sharks to mammals, but E-I-P-P-P was found only in reptiles and chondrichthyans, suggesting it may be the primordial sequence (**Supplementary Data Sheets 3-B**, **4**). The Y-based signaling motif in the cytoplasmic tail was present in all these sequences with a Y-[E/G/V/T/A]-N-[V/I/M/T] consensus, which is consistent with a GRB2/GADS binding motif (YxNx) (**Supplementary Data Sheet 4**).

**Figure 4:**
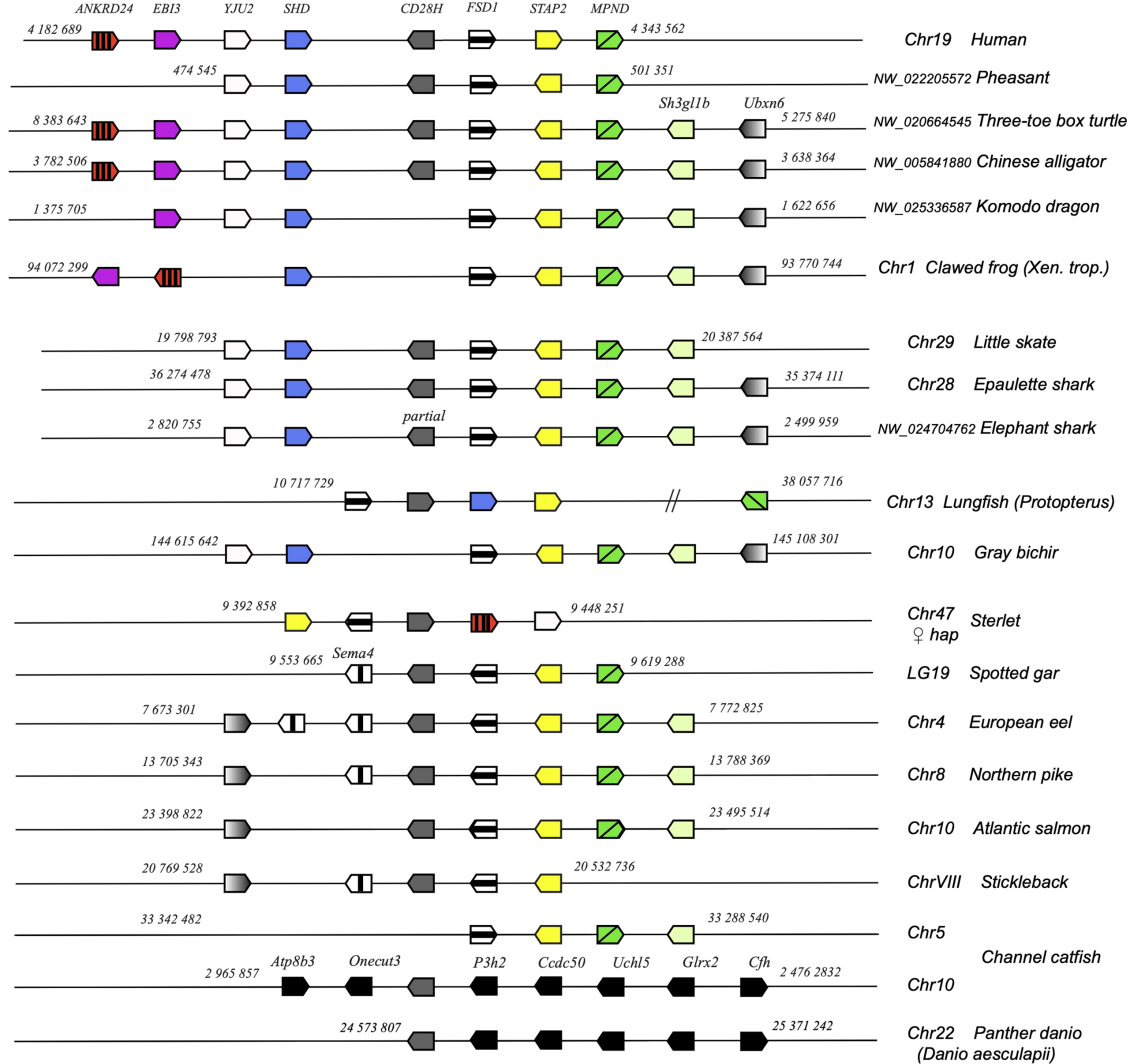
Conserved synteny blocks with CD28H (dark grey). Only conserved markers are represented in the regions of interest, of which start and end location are indicated in the reference NCBI chromosome/LG of each species.

**Figure 5:**
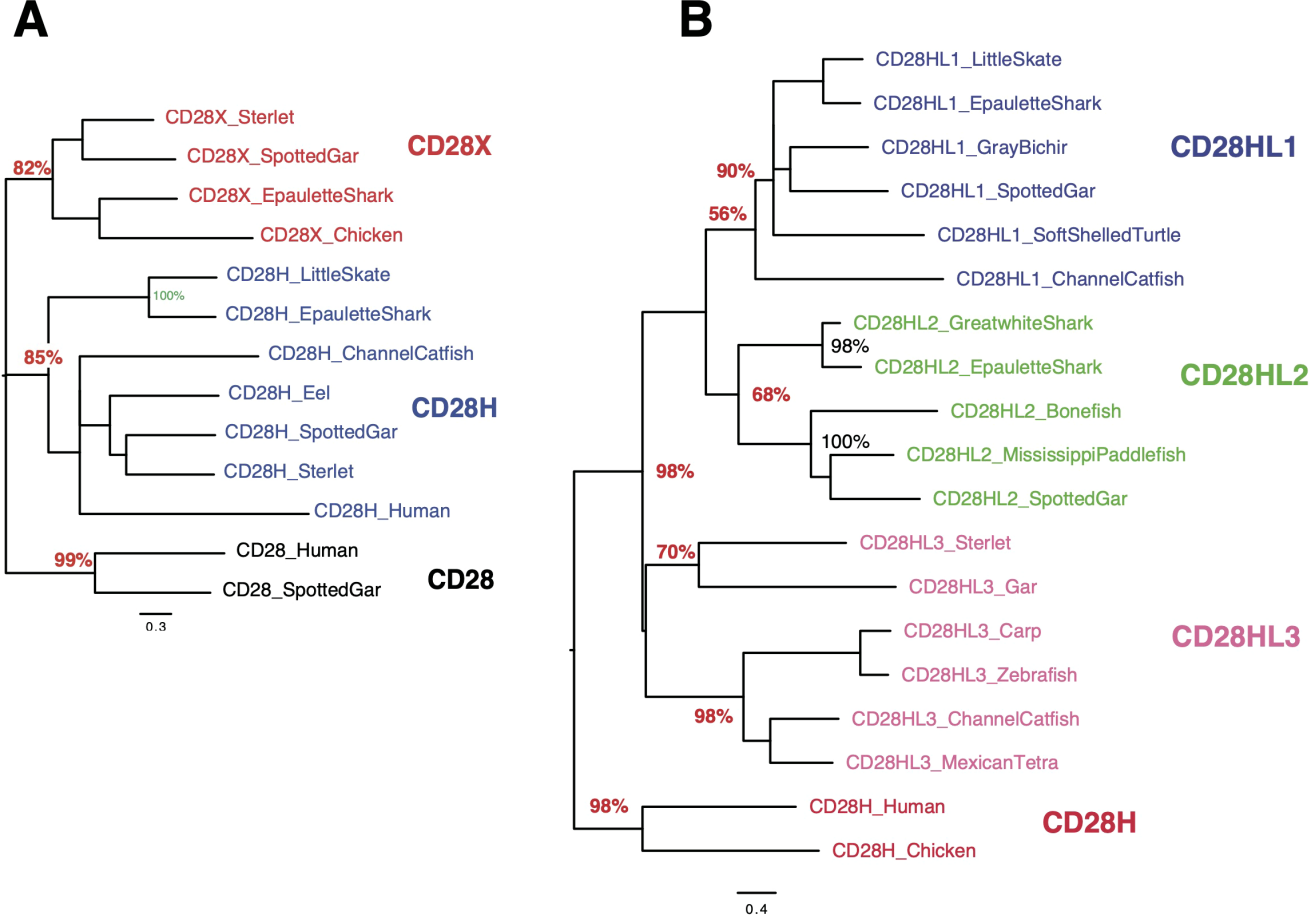
Evolutionary analysis of CD28, CD28H and CD28X **(A)** or CD28H and CD28HL1-3 **(B)**. As in **Figure 1C**, the evolutionary history was inferred by using the Maximum Likelihood method and JTT matrix-based model. The trees with the highest log likelihood are shown, and the percentage of trees in which the associated taxa clustered together is shown next to the branches.

*cd28h* genes were part of a conserved synteny block, with *fsd1* and *stap2* conserved from Chondrichthyes to tetrapods and teleosts (**Figure 4**). However, in channel catfish and other siluriformes as well as in cypriniformes and clupeiformes, *cd28h* was present in another genomic context, although with all the canonical motifs. CD28H sequences clustered as a separate branch in phylogenetic trees (**Figure 5A**). Hence, we propose that CD28H is an ancient member of the CD28 family, which was present in common ancestors of Chondrichthyes and Osteichthyes, and has been retained in most jawed vertebrates.

### Four paralogs of *cd28h* are found across Chondrichthyes and Osteichthyes but have been lost in human and mouse

When searching for CD28H sequences in Chondrichthyes and basal fish groups, we found other homologous sequences, which were not found in the *cd28h* synteny groups and had different motifs. These sequences were present in different ranges of species and had all been lost in human. The spotted gar has the largest repertoire of CD28 family members, as illustrated in **Figure 3**. These sequences shared the hallmarks of CD28 family members, with distinctly conserved signaling motifs (**Figure 3**, **Supplementary Data Sheet 4**). Three sequences, which we named CD28HLike1-3 (**Figures 3A–C**), were closest to CD28H while the fourth paralog was rather distant from CD28, CTLA4, ICOS and CD28H and was named CD28X. Phylogenetic analysis supported this annotation, thus defining four additional members of the CD28 family (**Figures 5A, B**). We therefore analyzed the domain structure and synteny for these 4 genes across jawed vertebrates.

#### cd28hl1

The CD28HL1 V domain contained a conserved motif YxC[E/K/Q/N-polar]xxx-E-I-P similar to CD28H in the CDR3 region (**Supplementary Data Sheets 3-C**, **4**); the EIP motif was not followed by P except in Chondrichthyes (**Supplementary Data Sheet 4**). In contrast, the highly conserved Y[E/D]SF motif located in the cytoplasmic region was different from the YEDM found in CD28H, suggesting that it may recruit different kinases or mediate other signals. *cd28hl1* was found in little skate, epaulette shark and elephant shark, hence is present in the three main groups of Chondrichthyes (**Supplementary Data Sheet 3-C**). It was part of a synteny group conserved between Chondrichthyes and tetrapods, including for example *man2b, kiaa0232* and *tcb1d14* (**Supplementary Data Sheet 5**). This gene was also found in Chondrostei (sterlet), bichir and spotted gar, in the same conserved synteny block, as well as in teleost species (northern pike, rainbow trout and channel catfish). In tetrapods, *cd28hl1* was found in amphibians (*Xenopus tropicalis*), turtles, and birds. We found it neither in Squamata (snakes and lizards) in the corresponding synteny block nor in mammals. In particular, it was absent from the human genomic region despite the conserved synteny of the associated markers (**Supplementary Data Sheet 5**).

#### cd28hl2

This set was found both in Chondrichthyes and Osteichthyes but appeared to be lacking in many species across the groups. It was located in a conserved synteny block with *slbp, tacc3* and *tmem129* (**Supplementary Data Sheet 6**), and showed a conserved Y-x-C-[E/K/Q/N-polar]-xxx-E-I-P-[P/A/L/V]-[P/F/L] motif in the CDR3 region. A PxP motif was therefore present in most but not all species (**Supplementary Figure S3-D** in **Supplementary Data Sheet 3**). No well-conserved Y-based motif was identified in the cytoplasmic region after the transmembrane region, but a Y-x-N-x motif was present in almost all species closer to the C terminus. In Chondrichthyes, *cd28hl2* was found in great white shark and epaulette shark, but not in rays or elephant shark. It was present in bichir, paddlefish, and spotted gar. In teleosts, we found it in basal Elopomorphs such as eel and bonefish, but not in other teleosts. It was also present in the lungfish (both in the genome assembly and as a Transcriptome Shotgun Assembly (TSA) sequence GGXP01048466, but is apparently missing in tetrapods.

#### cd28hl3

A last set of sequences related to CD28H was found in bichir, spotted gar, sterlet, and among teleosts in cypriniformes and siluriformes taxa. CD28HL3 had its own conserved motifs, and is located in a synteny block with *prkab1b*, *pla2g1b* and *tmem233* (**Supplementary Data Sheet 7**). This genomic region was present in human with the same set of markers, but did not contain a *cd28h-*like sequence. A YxCxxxxx[I,A,V,L]P motif was present in the CDR3 region, while a typical ITIM/ITSM was found in the cytoplamic region ([I,V]xYxxL (X)_n_VTYxxV) in many species (**Supplementary Data Sheet 3-E**). We failed to detect *cd28hl3* sequences in Chondrichthyes.

#### cd28x

Another set of CD28 related sequences was found in Chondrichthyes (in sharks and rays) and in tetrapods, which was 15 to 20% similar to all CD28, CTLA4 and CD28H within a given species as illustrated in gar in **Figures 3B, C** (see also **Figure 5A**). We named this gene *cd28x*, as we believe it is a true member of the CD28 receptor family based on its conserved motifs: the CD28X sequence of the V domain CDR3 region shows a conserved motif YxCx_6_PPPx_4/5_GxG reminiscent of the typical CTLA4 signature, with PPP and a GxG motifs in the canonical positions (**Supplementary Data Sheets 3-F**, **4**). A potential YxxxxT dimerization motif could be present in CD28X transmembrane region, but not in all species (**Supplementary Data Sheet 4**). CD28X also has a highly conserved EYEDM signaling motif in the cytoplasmic region, which is a potential PI3K recruitment site (**Supplementary Data Sheet 4**). *Cd28x* genes were found in a conserved synteny block with the markers *pudp, sts, anos1, nlgn4x* and *pnpla4*, both in Chondrichthyes and Osteichthyes (**Supplementary Data Sheet 8**). We found *cd28x* in sterlet and spotted gar, but we could not detect it in any teleost. In tetrapods, typical *cd28x* was found in turtles, birds, and crocodiles, but not in squamata. This gene seems to have been lost in mammals.

### PD-1 orthologs in Chondrichthyes, and basal Osteichthyes and teleosts

PD-1 is encoded by the gene *pdcd1* and contains an Ig V domain, a stalk, a transmembrane region, and a cytoplasmic domain with ITIM and ITSM. It delivers co-inhibitory signals, and its expression is induced by TCR signaling. The CDR3 loop of the V domain does not have conserved Pro residues. Although its expression wanes at the end of an immune response, it remains high in particular T cell subsets like tissue-resident memory T cells, exhausted CD8^+^ T cells, and T follicular helper cells. It is considered a major immune checkpoint receptor and is also expressed on T regs, in which it conveys suppressive functions.

**Figure 6:**
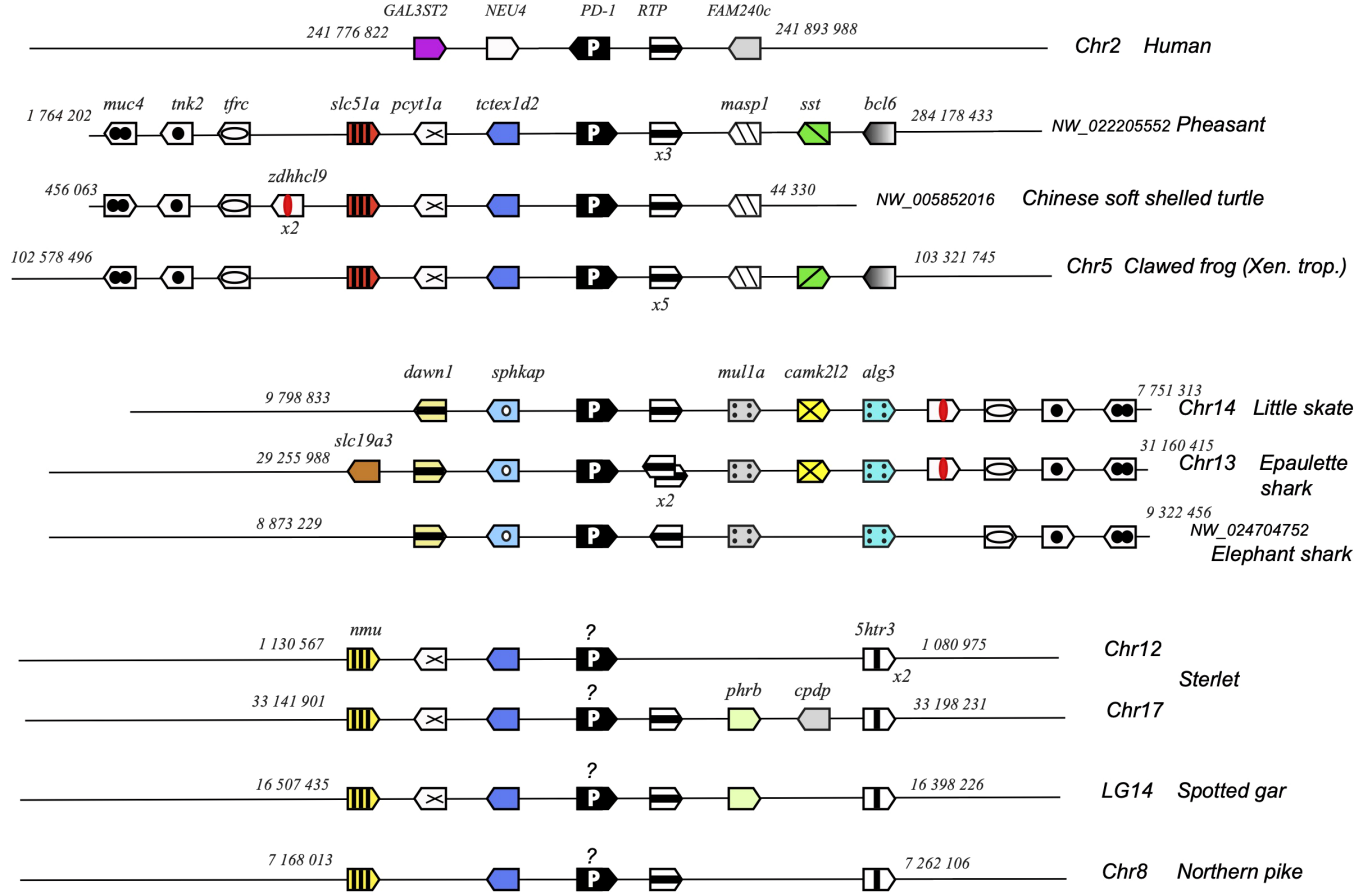
Conserved synteny blocks with PD-1 (white P on black background). Only conserved markers are represented in the regions of interest, and start and end locations are indicated in the reference NCBI chromosome/LG of each species. PD-1 genes encoding a divergent IgV domain are indicated with a “?”.

We did not find PD-1 orthologs in teleost fish in our previous surveys (24, 32). However, orthologs of B7H1/PDL1 and B7DC/PDL2 were identified in several bony fish species (32, 33), suggesting that a homolog of PD-1 might be present. Following the same approach as for CD28H, we could find potential PD-1 homologs in Chondrichthyes and basal groups of bony fish, including the sterlet and the spotted gar (**Figure 3**, **Supplementary Data Sheet 3-G**, **Figure 6**). Homologs of these sequences were then identified in teleosts such as the northern pike through conserved synteny analysis (**Supplementary Data Sheet 3-G**). These sequences clustered with PD-1 from mammals and reptiles in phylogenetic trees (data not shown), and they contained highly conserved ITIM-related motifs in their cytoplasmic domain apart from Chondrichthyes (**Supplementary Data Sheet 3-G**, **Figure 3A**). However, the ITSM consensus TxYxxV/I conserved in Amniotes seems to be divergent in the frog and fish sequences, and the V domain was also very divergent in bony fish (**Supplementary Data Sheet 3-G**): the canonical C^23^ and W^41^ and other key residues of the V domain were absent from sterlet, spotted gar, and teleost sequences.

*Pdcd1* is not part of a large stable synteny set: even within tetrapods, the syntenic region of this gene is rather different between human/mammals and reptiles or amphibians (**Figure 6**). However, *pdcd1* was consistently linked to the markers *pcyt1a, tctexd2* and *rtp* in both tetrapods and bony fish groups, and to the markers *muc4, tnk2, tfrx, zdhhcl9* and *rtp* in tetrapods and Chondrichthyes (**Figure 6**).

Taken together, these structural features and genome locations indicate that Chondrichthyes and bony fish may possess a *pdcd1* ortholog, at least in Osteichthyes. However, the degeneracy of the V domain sequence in bony fish questions conservation of the mechanisms leading to inhibitory signaling via the conserved cytoplasmic motifs.

### No member of the CD28 family was found in Agnathans, and the immune checkpoint receptor IgSF-11 appears to be the closest relative of CD28 present in lamprey and human

We then scanned lamprey and hagfish databases for IgSF members similar to CD28 or related sequences, but no convincing orthologues could be found using blast searches, domain profiling (HMMSEARCH) using immunoglobulin domain PFAM profiles, or synteny-based characterization. The lack of hagfish or lamprey protein models containing Interpro motifs IPR008093 (CD28) or IPR040216 (CD28/CTLA4) or IPR039695 (present in TMIGD2 aka CD28H) also supported these observations. We also failed to find any convincing homolog of CD28, CTLA4 or ICOS in genomes of non-vertebrate deuterostomes including acorn worm (*Saccoglossus kowalesvskii*, hemichordate), sea urchin (*Strongylocentrotus purpuratus*, echinoderm), sea squirt (*Ciona intestinalis*) and lancelet (*Branchiostoma floridae*)(chordates).

The closest related sequence identified in sea lamprey (*Petromyzon marinus*) appeared to be a homolog of genes encoding the immune checkpoint IgSF-11/V-Set and Immunoglobulin Domain Containing 3 (VSGIG3) (LOC116948162) and also A33 (LOC116948206), belonging to the CTX family. In human, while A33 was not clearly linked to immune regulatory functions, Ig-SF11 was identified as the ligand of VISTA, a member of the B7 family (34) that is an immune checkpoint of growing importance in tumor immunology (35, 36). This membrane protein with 2 IgSF domains inhibits T-cell responses and promotes an immune-suppressive microenvironment in tumors. Mouse IgSF-11 controls osteoclast differentiation through modulation of Pyruvate kinase isozymes M2 (PKM2)-mediated glucose metabolism, a mechanism requiring interaction with the adaptor protein postsynaptic density 95 (PSD-95) via 75 C-terminal amino acids of IgSF1 (37, 38). Interestingly, the C-terminal sequences of human and lamprey IgSF-11 are highly conserved, suggesting that they might interact with the same intracellular partners (**Supplementary Data Sheet 9**).

## Discussion

**Figure 7:**
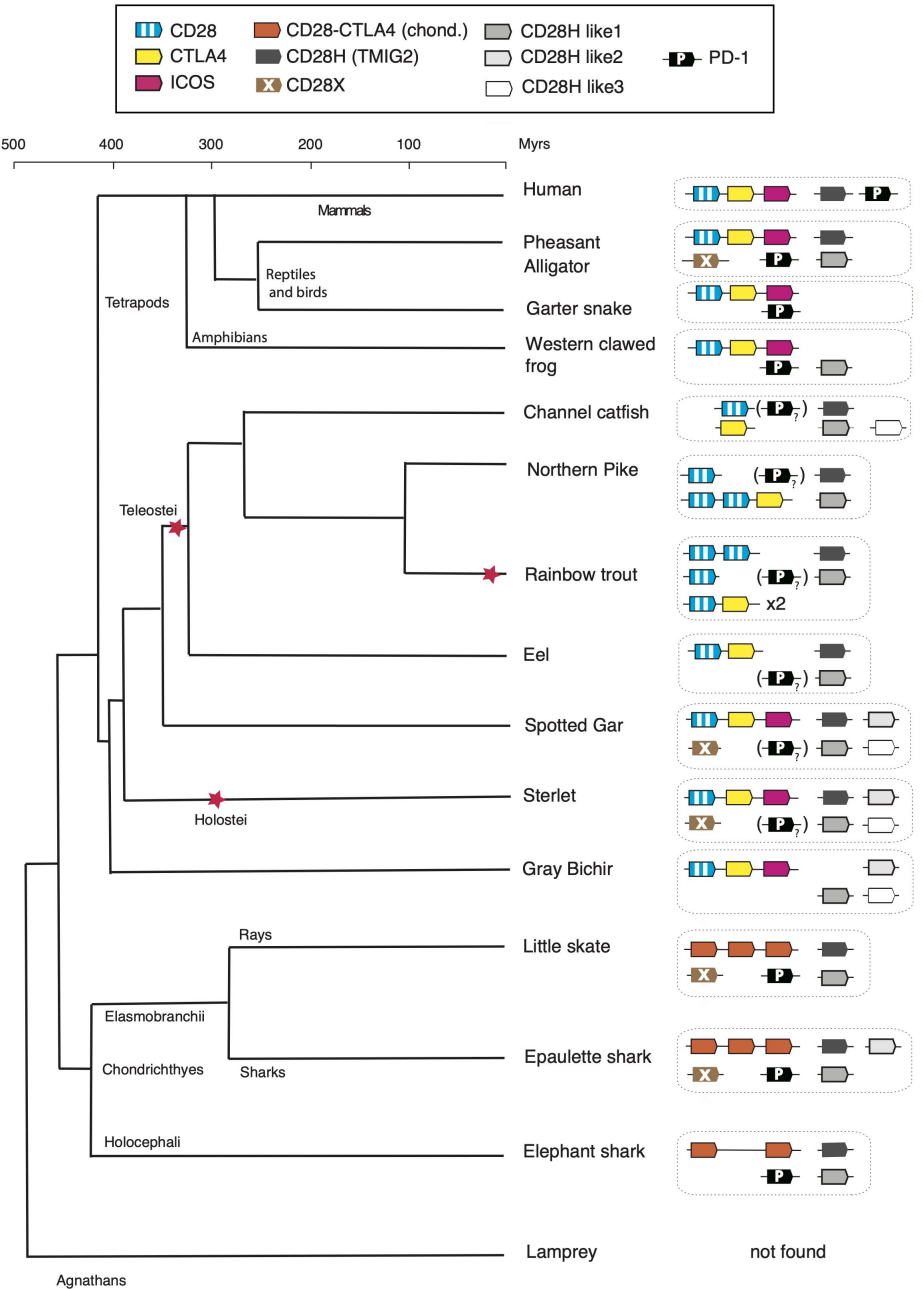
Evolution of the repertoire of CD28 family members in vertebrates. The “?” and “()” refer to the PD-1 homolog with degenerated IgV domain in bony fish species. The red stars denote whole genome duplication events.

In this work, we found (co)orthologs of all members of the CD28 family both in the Chondrichthyes and basal Osteichthyes groups, but not in Agnathans, suggesting that these key regulators of T cell activation appeared in early jawed vertebrates in concert with the adaptive immune system based on VDJ-diversified Ig domain Ag-specific receptors. Four additional members of the family were identified, which were present in both Chondrichthyes and Osteichthyes, some even in the tetrapod lineage but all absent in human (**Figure 7**). Thus, the composition of the CD28 family appears to be CD28/CTLA4/ICOS, CD28H, CD28HL1, CD28HL2, CD28HL3, CD28X and PD-1, as defined by domain structure, conserved motifs and conserved synteny. This diverse repertoire was already well defined in the common ancestors of Chondrichthyes and Osteichthyes and provides a striking example of evolution by diversification-decimation/standardization in contrast with a model of progressive increased complexity of the immune system from basal vertebrates to tetrapods. It is interesting to note that the retention rate of these genes is highly variable: *cd28hl1* is present in almost all vertebrate groups including marsupials, missing only in eutherians, while *cd28hl2* seems to have been lost in chimeras, rays, teleosts and tetrapods, and *cd28x* has been retained by particular groups across vertebrates including birds and crocodiles (but not squamata), basal bony fish groups (but not teleosts) and some chondrichthyans. These various distribution ranges likely relate to the functional specificities of the receptors, and emphasize key roles of the most conserved members of the family including CD28/CTLA4, ICOS, CD28H, and PD1. Chondrichthyes and ancient Osteichthyes such as chondrosteans and holosteans show the highest retention rate of CD28 family members, a trend which might be interesting to compare with other gene families of the immune system.

Understanding the co-stimulatory/inhibitory properties of these diverse receptors requires experimental work, but the analysis of conserved motifs provides interesting hints. The YMNM cytoplasmic motif found in human CD28, which contains motifs for the binding of PI3K (YxxM) and GRB2/GADS (YxNx) is conserved in lungfish, sterlet, and spotted gar. In teleosts, the motif is rather variable or even absent, although CD28 is still a positive co-stimulatory receptor (at least in rainbow trout) (20). The GxG motif in the CDR3 loop is present in chondrichthyan homologs of CD28 and in basal bony fish but absent in teleost, tetrapods, and mammals. In CTLA4, the GxG motif and a cytoplasmic YVxM are also well conserved in basal groups of bony fish, suggesting that the functions of CTLA4 may be similar in these species and in mammals. Interestingly, the dimerization motif in the TM is conserved in CD28, but restricted to tetrapods in CTLA4; whether the CTLA4 orthologs function as monomers simply competing with CD28 is an interesting question. In Chondrichthyes, the CD28/CTLA4 orthologs have features of both receptors, and it is difficult to make any prediction about their functions. Of note, the genes located in the first, second, and third positions in the genome, respectively, share similarities between Chondrichthyes species and might have different roles. We did not find ICOS features in chondrichthyan sequences, but its cytoplasmic signaling motif was fully conserved between tetrapods and bichir, gar and sterlet, suggesting that it is likely a positive co-stimulatory receptor in all these species. Interestingly, a dimerization motif is present in fish supporting that this receptor is probably expressed as a homodimer as in human (although this motif is not present in human or other tetrapod ICOS) (10). The ITIM-ITSM motif present in tetrapod PD-1 was also found in all bony fish PD-1-like sequences, although the ITSM consensus sequence was not canonical. While this difference might affect signal reinforcement and intensity, these receptors may be inhibitory, like their mammalian counterparts. Similarly, the YEDM cytoplasmic motif of CD28X was strictly conserved, with PI3K-binding site and possibly a degenerated GRB2/GADS binding site (YxNx). Future studies will be required to confirm whether it delivers positive co-stimulation. Hypotheses regarding the type of co-stimulation associated with CD28H and its paralogs are more complicated because their signaling motifs appear to be less conserved. However, a YxNx motif was consistently present in all CD28H sequences (YxNM in lungfish, sterlet, gar, eel, pike and salmon), supporting the idea of a positive co-stimulatory function, while the conserved ITIM present in CD28HL3 suggests the inhibitory role. A YxSx was present in most CD28HL1, while Y residues were present in the CD28HL2 cytoplasmic domain, however they were not part of any known regulatory motifs and offered little hint of function.

CD28 family members primarily bind receptors of the B7 family, another subset of IgSF proteins typically comprising V and C domains. Genes encoding B7 family members are located in MHC paralogons, hence originate from a precursor located in the proto-MHC region containing *b2m* before the 2R whole genome duplication and the emergence of vertebrates (39, 40). While no clear counterpart of B7 was found in Agnathans or non-vertebrate deuterostomes, it has been proposed that hagfish IGSF3 may be related to the common ancestor of the B7 family (39). Our work shows that a core repertoire of CD28 family members comprising a CD28/CTLA4/ICOS receptor, CD28H, CD28HL1 and 2, CD28X and PD-1 was present in the common ancestor of Chondrichthyes and Osteichthyes but apparently not in agnathans. In contrast to B7 family, we have no evidence of a gene that derives from a common ancestor(s) of CD28 family members that predated the 2R WGD and the emergence of vertebrates, and the apparent lack of these genes in Agnathans does not support this idea either. Of note, *cd28h*, which is one of the primordial members of the family and has been retained in almost all species, is located in a major MHC paralogous region in the human genome (19p13). Another interesting feature is the presence of a GxG motif in the G-strand of the V domain in several CD28 family members, including CTLA4. The J consensus FGxGTx(L/V)xV (41) is generally not present, but the GxG motif should produce a bulge conformation and indeed affects the affinity for the ligand (21). This GxG motif has not been retained in all CD28 family members, but it connects these genes to the set of IgSF members with joined VJ domains, comprising CD79, NITR, SIRP, CD8, NKp30, PRARP, that share a V domain architecture similar to that of rearranging antigen receptors (42, 43). While our work did not reveal the origin of the CD28 family, it shows that this family of receptors likely arose in early jawed vertebrates, and diversified when the Ig/TCR/MHC-based adaptive immunity emerged. This strongly supports the acquisition of new complex regulatory networks controlling lymphocyte activation by “second signals” delivery (39, 44, 45). Our findings reveal that CD28/B7 family members could play a key role in these networks across all jawed vertebrate groups. In contrast, it appears that mechanisms controlling the activation of agnathan lymphocytes rely on totally different mechanisms and receptors. Agnathan lymphocytes express different classes of somatically diversified antigen receptors based on leucine-rich repeats named Variable lymphocyte receptors (VLR). They can be classified into T-like and B-like cell subsets (46), likely require tight control of activation and probably have counterparts of second signals. However, VLRA, the Ag-specific receptors expressed by the agnathan T-like lymphocyte lineages, signal through receptor-proximal pathways different from the TCR/CD3 mediated pathways of jawed vertebrate T cells. VLR signaling pathways remain poorly understood, and may provide insights into lymphocyte co-stimulation partners and mechanisms in these species. Besides, the lack of MHC molecules certainly imposes fundamental differences. Another interesting point is that the specific features of CD28, CTLA4 and ICOS appeared after the divergence of Chondrichthyes and Osteichthyes; it will be interesting to elucidate positive versus negative signaling requirements associated with each of the three genes found in Chondrichthyes in the region corresponding to the *cd28/ctla4/icos* cassette.

Further research is needed to understand to what extent different members of the CD28 family interact with their respective ligand(s) and how cross-binding may connect different pathways, as PD-L1 (B7H1) interactions with PD-1 and B7-1 can be seen as a crossroad between inhibitory PD-1/PD-L1 and CTLA4/B7-1 signaling pathways (19). The range of expression of these receptors (and their ligands) will inform about their relative importance for the activation of T cells, NK cells, B cells and possibly other leukocytes. In summary, our findings suggest that the control of lymphocyte stimulation has evolved and specialized from a diverse repertoire of CD28-related receptors, during the evolution of the different groups of vertebrates.

## Data Availability

The original contributions presented in the study are included in the article/**Supplementary Material**, further inquiries can be directed to the corresponding author/s.
