# Supplementary material for: Extraordinary diversity of the CD28/CTLA4 family across jawed vertebrates
Source: Front Immunol. 2024 Nov 13;15:1501934. doi: 10.3389/fimmu.2024.1501934 (PMC11599192; doi:10.3389/fimmu.2024.1501934)
Supplement: Supplementary file 1 [file DataSheet1.pdf]

Supplementary file S1. List of species and genome assemblies used in this study.

Human, Homo sapiens, GRCh38.p14 (GCF_000001405.40)
House mouse, Mus musculus, GRCm39 (GCF_000001635.27)
Tasmanian devil, Sarcophilus harrisii, mSarHar1.11 (GCF_902635505.1)
Gray short-tailed opossum, Monodelphis domestica, mMonDom1.pri (GCF_027887165.1)
Common wombat, Wonbatus ursinus, bare-nosed wombat genome assembly (GCF_900497805.2)
Okarito brown kiwi, Apteryx rowi, aptRow1 (GCF_003343035.1)
Chicken, Gallus Gallus, bGalGal1.mat.broiler.GRCg7b (GCF_016699485.2)
Ring-necked Pheasant, Phasianus colchicus, ASM414374v1 (GCF_004143745.1)
Swan goose, Anser cygnoides, Taihu_goose_T2T_genome (GCF_040182565.1)
Komodo dragon, Varanus komodoensis, ASM479886v1 (GCF_004798865.1)
Green Anole, Anolis carolinensis, rAnoCar3.1.pri (GCF_035594765.1)
Western terrestrial garter Snake, Thamnophis elegans, rThaEle1.pri (GCF_009769535.1)
Chinese soft-shelled turtle, Pelodiscus sinensis, PelSin_1.0 (GCF_000230535.1)
Three-toed Box turtle, Terrapene Carolina triunguis, T_m_triunguis-2.0 (GCF_002925995.2)
Chinese Alligator, Alligator sinensis, ASM45574v1 (GCF_000455745.1)
American Alligator, Alligator mississippiensis, rAllMis1 (GCF_030867095.1)
Western clawed frog, Xenopus tropicalis, UCB_Xtro_10.0 (GCF_000004195.4)
West African lungfish, Protopterus annectens, PAN1.0 (GCF_019279795.1)
Gray bichir, Polypterus senegalus, ASM1683550v1 (GCF_016835505.1)
Mississippi paddlefish, Polyodon spathula, ASM1765450v1 (GCF_017654505.1)
Sterlet, Acipenser ruthenus, fAciRut3.2 maternal haplotype (GCF_902713425.1)
Bonefish, Albula goreensis, AGOR_1.0 (GCA_022829145.1)
Spotted gar, Lepisosteus oculatus, LepOcu1 (GCF_000242695.1)
European eel, Anguilla anguilla, fAngAng1.pri (GCF_013347855.1)
American eel, Anguilla rostrata, ASM1855537v3 (GCF_018555375.3)
European conger, Conger conger, fConCon1.1 (GCF_963514075.1)
Three-spined stickleback, Gasterosteus aculeatus aculeatus, GAculeatus_UGA_version5 (GCF_016920845.1)
European seabass, Dicentrachus labrax, dlabrax2021 (GCF_905237075.1)
Western Mosquitofish, Gambusia affinis, SWU_Gaff_1.0 (GCF_019740435.1)
Southern bluefin tuna, Thunnus maccoyii, fThuMac1.1 (GCF_910596095.1)
Nile tilapia, Oreochromis niloticus, O_niloticus_UMD_NMBU (GCF_001858045.2)
Northen Pike, Esox lucius, fEsoLuc1.pri (GCF_011004845.1)
Rainbow trout, Oncorhynchus mykiss, USDA_OmykA_1.1 (GCF_013265735.2)
Atlantic salmon, Salmo salar, Ssal_v3.1 (GCF_905237065.1)
Channel Catfish, Ictalurus punctatus, Coco_2.0 (GCF_001660625.3)
Common carp, Cyprinus carpio, ASM1834038v1 (GCF_018340385.1)
Sinocyclocheilus anshuiensis, SAMN03320099.WGS_v1.1 (GCF_001515605.1)
Razorback sucker, Xyrauchen texanus, RBS_HiC_50CHRs (GCF_025860055.1)
Zebrafish, Danio rerio, GRCz11 (GCF_000002035.6)
Panther danio, Danio aesculapii, fDanAes4.1 (GCF_903798145.1)

Red-bellied piranha, Pygocentrus nattereri, fPygNat1.pri (GCF_015220715.1)
Mexican tetra, Astyanax mexicanus, AstMex3_surface (GCF_023375975.1)
Striped catfish, Pangasianodon hypophthalmus, fPanHyp1.pri (GCF_027358585.1)
Chinese Large-mouth catfish, Silurus meridionalis, ASM1480568v1 (GCF_014805685.1)
Elephant Shark, IMCB_Cmil_1.0 (GCF_018977255.1)
Epaulette Shark, sHemOce1.pat.X.cur. (GCF_020745735.1)
Smaller spotted catshark, sScyCan1.1 (GCF_902713615.1)
Great white shark, sCarCar2.pri (GCF_017639515.1)
Little Skate, Leucoraja erinacea, Leri_hhj_1 (GCF_028641065.1)
Japanese Lamprey, Lethenteron camtschaticum, IMCB_Ljap_1.0 (GCA_018977245.1)
Hagfish, Myxine glutinosa, kmMyxGlut1.1 (GCA_964187855.1)
