# Supplementary material for: Extraordinary diversity of the CD28/CTLA4 family across jawed vertebrates
Source: Front Immunol. 2024 Nov 13;15:1501934. doi: 10.3389/fimmu.2024.1501934 (PMC11599192; doi:10.3389/fimmu.2024.1501934)
Supplement: Supplementary file 2 [file DataSheet2.pdf]

Supplementary file S2. Motifs and domains present in all protein models used in this study for CD28, CTLA4, ICOS, and chondrichthyan CD8/CTLA4 homologs

Relevant motifs discussed in the text are in red. Note also the conserved C residues in the TM sequences of ICOS (in blue), which might play a role in dimerization. After chondrichthyan sequences, the number refers to the position in the locus.

	Species	CDR3 region	TM	Y based Signaling motif	Second Y based Signaling motif	Accession #
CD28	Human	YFCKIEVMYPPPYLDNEKSNG	GVLACYSLLVT	DYMNM	PYAPP	AJ517504
	Mouse	YFCKIEFMYPPPYLDNERSNG	GVLFCYGLLVT	DYMNM	PYAPA	BC064058
	Swan goose	YFCKIEAMYPPPYVYNEKSNG	GLLAFYSILIT	DYMNM	PYAPT	XP_013027129
	Frog (Xenopus tropicalis)	YFFRKEDMYPPPYTCYQDNGT	GVLAAYSLTIT	EYINV	PYAPA	XP_017952834
	Lungfish (Protopterus)	YECIIEVMYPPPYQKYGNGTL	LVLGIYGIFMT	DYMNM	PYAPQ	XP_043916657
	Bichir (Polypterus)	YSCDMLTLYPPPFLKIMGQKT	AFLALYSLITS	DYMNM	RVVHP	XP_039613786
	Sterlet	YYCQLNIMYPPPFRSPKGNGT	VMLAIYGMTVS	DYMNM	GVLHP	XP_033899352
	Paddlefish	YYCQLNILYPPPFRNPKGTGT	AVIVLYGVIIS	EYMNM	GVCHP	XP_041120529
	Gar	YSCDIEVLYPPPYRKINGSTT	CVILLYSLVIT	DYMNM	GVLHP	XP_015214827
	European eel	FACNAERLYPPPYLKSPGVEN	GVAAAYSLIMT	DYMNM	GVQHP	XP_035264839
	Pike	YSCKAEKLWPPPYKEDSVETL	GLLTVYSIIAT	VYMNV	RIQQP	XP_010898663
	Pike	YSCKAERMWPPPYKEDTVETM	GVLFFYSVIIT	IYMNV	RVQKP	XP_010898665
	Pike	YTCKVQNTYPPPVEDVKEMCQ	WINLVYGLAVT	DYMNT	GVQHP	XP_010891418
	Rainbow trout	YICRAERLWSPPYKVDCVHTL	GVLLLYSTILT	TYLNI	RVQYP	XP_036831245
	Rainbow trout	YTCEGQPMCPPPIEKLPDETQ	WVTIIYGLAVT	DDMNI	GVQHP	XP_021464608
	Rainbow trout	YTCEGYPMYPPPIEKVPDEPQ	WVTTIYGLVVT	DYMDI	GVQHP	NP_001118004
	Rainbow trout	YTCEGQPMYPPPIEKVQDETQ	WITIIYGLAVT	DYMNI	GVRHP	XP_021427178
	Rainbow trout	YTCIAERMRSPLPYQEDSVQT	CGFLLYSIIIT	DYVNM	RVQHP	XP_036815388
	Channel catfish	YTCTAEKSYPPPMVTIQEEPQ	GILSVYCLIIT	DYMNM	GIHHP	XP_017313746
CTLA4	Human	YICKVELMYPPPYYLGIGNG	SGLFFYSFLLT	VYVKM	PYFIP	NP_005205
	Mouse	YLCKVELMYPPPYFVGMGNG	LGLFFYSFLVT	VYVKM	PYFIP	NP_033973
	Swan goose	YVCKMERMYPPPYFMNKGNG	SGFFLYSFIIS	VYVKM	PFHIT	XP_013027132
	Frog (Xenopus tropicalis)	YICKLDIMYPPPYRTTEGNG	LVMFLYSMFIT	NYEKM	PYYIR	XP_012825450
	Lungfish (Protopterus)	YICKMEIMYPPPYRVRMGNG	AGLLLHSIVMT	DYENM	PYQIL	XP_043916244
	Bichir (Polypterus)	YRCKVEILYPPPYRLGLGSG	VCTVIVIISIL	EYVDT	RYVMF	XP_039610963
	Sterlet	YKCRVEIMYPPPYRQRVGNG	ACTVIIIAAIL	DYVDM	RYVIL	XP_033900514
	Paddlefish	YKCRVEIMYPPPYRQRVGKG	ACTVIIIAAIL	DYVDM	RYVIL	XP_041119710
	Gar	YRCSLEIMYPPPYRQRYGNG	VLAFIIIMTIL	EYVDM	GYENF	XP_015214835
	European eel	YRCQLEVLYPPPYLMKYGNG	VMAIFIIIFII	MYPHM	GYENF	XP_035266074
	Pike	YRCAIEVMYPPPYLRVFGNG	SVAWVSLLLMV	no conserved motif	GYENF	XP_012986927
	Rainbow trout	YYCGIEVMYPPPYLRRFGNG	GLVAANILMVI	no conserved motif	GYENF	XP_036831244
	Rainbow trout	YRCAIEVLYPPPYLRTFGNG	GLAAVLIVISA	no conserved motif	DYENF	NP_001118005
	Channel catfish	YRCQIDIIFPPPYISKFGNG	LSAILITTTIT	no conserved motif	DYENF	XP_017324683
ICOS	Human	YFCNLSIFDPPPFKVTLTGG	AFVVVCILGCI	EYMFM	no conserved motif	XP_047299978
	Mouse	YFCSLSIFDPPPFQERNLSG	AFVVVLLFGCI	EYMFM	no conserved motif	XP_006496201
	Three toed box turtle	YLCCLEILLPAPYIDCRVNE	AFSMVSCICCI	EYMPM	no conserved motif	XP_026503133
	Frog (Xenopus tropicalis)	YTCEIRIFYPPPFRSIIINE	GLAVFLFLCCI	EYMPM	no conserved motif	XP_031749766
	Bichir (Polypterus)	YICKVEKRKPFPYEDGVGVG	GILLLYSVAIS	EYMDM	no conserved motif	XP_039613787
	Sterlet	YVCRVEIRSPPPFVYAEGTG	GLLLLYSIVIT	EYMDM	no conserved motif	XP_058889561
	Paddlefish	YVCRVEVRSPPPFVYGEGTG	GLLLLYSIIIT	EYMDM	no conserved motif	XP_041120674
	Gar	YVCRVHRKLPLPFVSRDGDG	AFSIIYSISVT	EYMDM	no conserved motif	XP_015214822
an hom	Little skate (1)	YFCKVEKMFPLPYTQATGNG	SLFLLYSIVVT	EYINM	no conserved motif	XP_055494224
	Little skate (2)	YICNTRKTYPPPYLTISGQW	ALLLLYSVSVT	VYINV	no conserved motif	XP_055494225
	Little skate (3)	YFCKIELLYPPPFESSEGSG	TLCMINIFFLT	FYQEP	no conserved motif	XP_055494226
	Catshark (1)	YICQIEKMHPPPYIKSNGNG	AIFILYSVFIT	EYVNM	no conserved motif	XP_038642918
	Catshark (2)	YFCQVSKTHPPPYFESPGQW	ILLFLYSVSVT	IYINV	no conserved motif	XP_038646294
	Catshark (3)	YFCQIEKLYPPPYEKKQGDG	FLTMGILGGLT	VYERM	no conserved motif	XP_038642929
	Epaulette shark (1)	FFCKIEKLYPPPYEGKQGNG	LLIMGILSFLT	IYSIV	no conserved motif	XP_060684085
	Epaulette shark (2)	YICVVLNTHPPPFYESSGQW	FLPTIILIVLA	LYSAS	no conserved motif	XP_060684151
	Epaulette shark (3)	YICHMAKIHPPPYIEGKGDG	TQSFMIVLVIA	FSSIF	no conserved motif	XP_060684086
	Elephant shark (1)	YYCKVTKMHPPPIINSIGNG	LVFLLYSIVIT	EYVNM	no conserved motif	XP_007888795
	Elephant shark (2)	YICQIEKMHPPPYIKSNGNG	AIFILYSVFIT	EYVNM	no conserved motif	XP_007888798
?	Lamprey	–		–		–
