# Supplementary material for: Extraordinary diversity of the CD28/CTLA4 family across jawed vertebrates
Source: Front Immunol. 2024 Nov 13;15:1501934. doi: 10.3389/fimmu.2024.1501934 (PMC11599192; doi:10.3389/fimmu.2024.1501934)
Supplement: Supplementary file 3 [file DataSheet3.pdf]

Supplementary file S3. Multiple sequence alignments of CD28 family members across representative vertebrate species.

Alignments were performed using clustal omega at https://www.ebi.ac.uk/jdispatcher/msa/clustalo. Sequences motifs discussed in the text, which are located in the CDR3 neighborhood in the V domain or in the intracytoplasmic region (tyr-based signaling motifs) are highlighted in yellow.

See also Supplementary files S2 and S4.

A-CD28-CTLA4-ICOS and chondrichthyan related sequences. Multiple alignment.

```
CD28-Hosa-AJ517504                                            ---------------------------MPC-GLSALIMCPKGM-VAVVVAVDD------G    25
CD28-Mumu-BC064058                                            -----------------------------------------MT-LRLLFLALN------F    12
CD28-SwanGoose-XP_013027129                                   -----------------------------------------ML-LGILVVLCF------I    12
CD28-Green_Anole-XP_003223584-NoLOC                           ------------------------------------MSRPSWILFTTLSIIHL------A    18
CD28-Xenopustrop-XP_017952834-NoLOC                           -------------------------------------MCL-WMAF----LLSC------I    12
CD28-Protopterus-XP_043916657-NoLOC                           ------------------------------------MVCI-------MIIAIL------I    11
CD28-Polypterus-XP_039613786-LOC120531949                     -----------------------------------------MILPAL-LVM--------A    10
CD28-MsPaddlefish-XP_041120529-LOC121323502                   -----------------------------------------MLTVIFYYIF--------L    11
CD28-Sterlet-XP_033899352-LOC117426134                        ---MSSALSSVAFQ------------SETP-KQKLPLLAVTMLTVIFYYIF--------L    36
CD28-Gar-XP_015214827-LOC106878821-LG12-27177048              --------------------------------MNHLTVRVSWLTVVFICVL--------I    20
CD28-Anguilla-XP_035264839-Chr3-si:dkey-1h24.6                -------------------------------------MNVIWITATLLSRG--------L    15
CD28-1-Esox-XP_010898663-Chr20-42937353-LOC105027959          -----------------------------M-NLKIKMEINLCITVFVM---------FCS    21
CD28-2-Esox-XP_010898665-Chr20-42928519-LOC105027960          ------------------------------------MDGYLWMTA-IL---------LAF    14
CD28-Esox-XP_010891418-Chr22-17852626-si:dkey-1h24.6          -------------------------------------MNICWILTILLSLC--------F    15
CD28-Onmy-XP_036831245-LOC118964336-Chr3-1-83647331           ------------------------------------MEGHLWMTTILLSFCPLILLSFSL    24
CD28-Onmy-XP_021464608-LOC110527567-Chr7-1-25301800           -------------------------------------MNVYWIPTILLS----------L    13
CD28-Onmy-NP_001118004-LOC100136274-Chr7-2-25315200           -------------------------------------MNVYWIPTILLS----------L    13
CD28-Onmy-XP_021427178-si:dkey-1h24.6-Chr18-27925782          -------------------------------------MNVYWIPTILLSLC--------L    15
CD28-Onmy-XP_036815388-LOC118943619-Chr22-1-50522004          ---------------------------------MKMMECNVWMTAVLLS--------LYL    19
CD28-Icpu-XP_017313746-si:dkey-1h24.6-Chr26                   -------------------------------------MKTSWLLATLLPLF--------L    15
CTLA4-Hosa-NP_005205                                          ----------------MACLGFQRH-KAQL---NLATRTWPCTLLFFLLFI----PV---    33
CTLA4-Mumu-NP_033973                                          ----------------MACLGLRRY-KAQL---QLPSRTWPFVALLTLLFI----PV---    33
CTLA4-SwanGoose-XP_013027132                                  -------------------------------------MLSVLVTVGFLCTA----TA---    16
CTLA4-Green_Anole-XP_003223584-NoLOC                          -------------------------------------MVALFLTIAFSSIV----AG---    16
CTLA4-Xenopustrop-XP_012825450-NoLOC-54,453,384               ------------------------------------MMRIPFIT-GIFCLI-----T---    15
Protopterus-CTLA4-XP_043916244-LOC122792727                   -----------------MILSITRPNHSEMWMCSQRTTLFHVFTILFFCNH----IK---    36
CTLA4-Polypterus-XP_039610963-LOC120530608                    ---------------------------------------MALTRLALVSVL----LC---    14
CTLA4-MsPaddlefish-XP_041119710-LOC121323053                  ---------------------------------------MIFKVIACISIC----TG---    14
CTLA4-Sterlet-XP_033900514-LOC117426713                       ---------------------------------------MIFKVIACISIC----IG---    14
CTLA4-Gar-XP_015214835-LOC102690085-LG12-27229939             ------------------------------------MNTMICKLIAFIGLC----SA---    17
Anguilla-CTLA4-XP_035266074-Chr3-NoLOC                        ---------------------------------------MIPALITLISLC----TH---    14
CTLA4-3-Esox-XP_012986927-Chr20-42914370-No                   MPLWRMHYSSISFQNDGLYKSLPHYSAGPF---HPVIMSLALLTGLC--LC----LP---    48
CTLA4-Onmy-XP_036831244-LOC110505466-Chr3-2-83647331          -------------------------------------MSLSLLTFLCLSLY----LP---    16
CTLA4-Onmy-NP_001118005-Chr22-2-50495067                      -------------------------------------MTLSLLTFLCLGLC----LP---    16
CTLA4-Icpu-XP_017324683-NoLOC-Chr6                            ---------------------------------------MI-AFVITLIVG----FP---    13
ICOS-Hosa                                                     ------------------------------------MK-SGLWYFFLFCLRIKVLTG-EI    22
ICOS-Mumu-XP_006496201                                        ------------------------------------MK-PYFCRVFVFCFLIRLLTG-EI    22
ICOS-SwanGoose-XP_013027130                                   ------------------------------------MK-SVAVTFCLLCFQFGALYG---    20
ICOS-Green_Anole-XP_062818357-LOC134293749                    -----------------------------------MKI-DAALVFGLLCFHFKTLHG-QI    23
ICOS-Turtle-XP_026503133-LOC112110999                         ------------------------------------MK-SGVLTFCLLCLQLEALCG---    20
ICOS-Alligator-XP_019347544-LOC102571456                      ------------------------------------MK-SGILTFCLLCFHFETLYG---    20
ICOS-Xenopustrop-XP_031749766-LOC100493997-Chr9-51,952,126    ---------------------------------MNVNL-TGFPLFILLV-----LHA-QL    20
ICOS-Polypterus-XP_039613787-LOC120531950                     ------------------------------------ML--PGLLLL--ALC----LG---    13
ICOS-MsPaddlefish-XP_041120674-LOC121323595                   ------------------------------MLKSESML--PKLIVI--CTF----IG---    19
ICOS-Sterlet-XP_058889561-LOC117426591                        ------------------------------------ML--PKLFVI--CTF----IG---    13
ICOS-Gar-XP_015214822-LOC107078819-LG12-27245174              ------------------------------------ML--SRLFLF--YLC----FG---    13
CD28-CTL4-Cami-XP_007888795-NoLOC                             -----------------------------------MRRYIVSLAIWSAWLLM---AI---    19
CD28-CTLA4-Cami-XP_007888798-LOC103176829                     ------------------------------MRLTNIMEHSRFLVLGF--ICLQ-IEL---    24
CD28-CTLA4-SCyCa-XP_038642918-LOC119958447                    ------------------------------------MQHLGQFAIFIICLLEA-FNM---    20
CD28-CTLA4-SCyCa-XP_038646294-LOC119962407                    ---------------------------------MEKMRNLGLFILCFPAFI----QL---    20
CD28-CTLA4-SCyCa-XP_038642929-LOC119958457                    ------------------------------------MECHRNLLVLFLWMRTV-WRL---    20
CD28-CTLA4-Leueri_XP_055494226_LOC129698880_Chr7_35060123-First        ------------------------------------MALLRCLLLAFLWMCMT-GRP---    20
CD28-CTLA4-Leueri_XP_055494225_LOC129698879_Chr7_35020935-Second       ---------------------------------MERMRNVWFFILCFPAVT----TL---    20
CD28-CTLA4-Leueri_XP_055494224_LOC129698878_Chr7_34987938-Third        ------------------------------------MKNLCHLATLLVCLQTVLLKV---    21
CD28-CTLA4-Hemoc_XP_060684085_LOC132817598_Chr7_112525810-First        ------------------------------------MACPRNLLVVFLWIYTA-QRL---    20
CD28-CTLA4-Hemoc_XP_060684151_LOC132817669_Chr7_112615465-Second       ------------------------------------------------------------    0
CD28-CTLA4-Hemoc_XP_060684086_LOC132817599_Chr7_112629451-Third        ------------------------------------MKRLGHFAAWFLCLLEV-LHT---    20
CD28-CTLA4-Carcar_XP_041057727_LOC121285321_Chr12_125148145-Third      ------------------------------------MKHRGQLVVLLTCFLGA-LNM---    20
CD28-CTLA4-Carcar_XP_041057149_LOC121285085_Chr12_125379474-First      ------------------------------------MDCLRNLLVVFLWMHTA-CKL---    20


CD28-Hosa-AJ517504                                            D-SQALAG------------------N-------------KILVKQSPMLVAYD---NAV    50
CD28-Mumu-BC064058                                            F-SVQVTE------------------N-------------KILVKQSPLLVVDS---NEV    37
CD28-SwanGoose-XP_013027129                                   P-AADVTE------------------N-------------KILVAQHPLLIVAN---KTA    37
CD28-Green_Anole-XP_003223584-NoLOC                           Y-TTVPPA------------------T-------------TMTMEQSPLHVVVS---QNA    43
CD28-Xenopustrop-XP_017952834-NoLOC                           T-VTECTD------------------S-------------SETQQQVVLVAVKQ---IEI    37
CD28-Protopterus-XP_043916657-NoLOC                           Y-TA---A------------------A-------------GLNISQPLTLTTNT---SDV    33
CD28-Polypterus-XP_039613786-LOC120531949                     A-ICCTEA-----------------------------------SQFQRKVVPVG---SSA    31
CD28-MsPaddlefish-XP_041120529-LOC121323502                   R-MVCSEE----------------------------------IIQPKFIIATEH---EEA    33
CD28-Sterlet-XP_033899352-LOC117426134                        R-MVYSQE----------------------------------IIQPKFIVATEH---AEA    58
CD28-Gar-XP_015214827-LOC106878821-LG12-27177048              G-LVISKD----------------------------R----NVSPPRVLRAAVH---GNV    44
CD28-Anguilla-XP_035264839-Chr3-si:dkey-1h24.6                L-AVAETE-------------------------------KC--PDMPIHRITPG---ANV    38
CD28-1-Esox-XP_010898663-Chr20-42937353-LOC105027959          L-DDVF---------------------T--------EHLDCKARAMYIQHVVPH---SQV    48
CD28-2-Esox-XP_010898665-Chr20-42928519-LOC105027960          W-GDVFTA------------------QS--------QAPNCQARTKYIQHVVPH---SQV    44
CD28-Esox-XP_010891418-Chr22-17852626-si:dkey-1h24.6          S-STANVI------------------SY--------NIF----DDLHVVQVRVN---GTT    41
CD28-Onmy-XP_036831245-LOC118964336-Chr3-1-83647331           S-DHLFTE------------------LS--------QHPNCKDRPLSIQSVSLH---SRV    54
CD28-Onmy-XP_021464608-LOC110527567-Chr7-1-25301800           S-SAANMI------------------SS--------NNCKDKLRMFNVVRVSVN---GTA    43
CD28-Onmy-NP_001118004-LOC100136274-Chr7-2-25315200           S-SAANMI------------------SS--------NNCKDKLRTFYVVRVSVN---GIA    43
CD28-Onmy-XP_021427178-si:dkey-1h24.6-Chr18-27925782          S-SAANMI------------------SS--------HNCKDKLRTFHVVRVSVN---GTA    45
CD28-Onmy-XP_036815388-LOC118943619-Chr22-1-50522004          S-DHVLT--------------------E--------QLPNCKDRPLYIQRVSLH---SRV    47
CD28-Icpu-XP_017313746-si:dkey-1h24.6-Chr26                   F-SCALPE----------------------------KQCQCKDNVLKIQRVTVH---GNV    43
CTLA4-Hosa-NP_005205                                          ------------------------------------FCKAMHVAQPAVVLASSR---GIA    54
CTLA4-Mumu-NP_033973                                          ------------------------------------FSEAIQVTQPSVVLASSH---GVA    54
CTLA4-SwanGoose-XP_013027132                                  ------------------------------------IAEVMEVSQPAMVLANRQ---GVA    37
CTLA4-Green_Anole-XP_003223584-NoLOC                          ------------------------------------FSKVMEVTQPVFTVVKSQ---GAA    37
CTLA4-Xenopustrop-XP_012825450-NoLOC-54,453,384               ------------------------------------NASGLKVTQPDIIVANRH---GKA    36
Protopterus-CTLA4-XP_043916244-LOC122792727                   ------------------------------------YLQAIKVYQPAVVVANRN---GEA    57
CTLA4-Polypterus-XP_039610963-LOC120530608                    ------------------------------------RAAALHIYQPYRVNANSD---GLA    35
CTLA4-MsPaddlefish-XP_041119710-LOC121323053                  ------------------------------------WGSAFEVFQPNRVEANNS---RVA    35
CTLA4-Sterlet-XP_033900514-LOC117426713                       ------------------------------------WGNAFEVFQPNRVEANNS---GVA    35
CTLA4-Gar-XP_015214835-LOC102690085-LG12-27229939             ------------------------------------GGNAFKVLQPYRVVA-SD---GVV    37
Anguilla-CTLA4-XP_035266074-Chr3-NoLOC                        ------------------------------------AGRALRVQQPYRVEA-RD---GEA    34
CTLA4-3-Esox-XP_012986927-Chr20-42914370-No                   ------------------------------------TWNALTVFQTYRAVS-QD---GEV    68
CTLA4-Onmy-XP_036831244-LOC110505466-Chr3-2-83647331          ------------------------------------VWNALRVTQPYRVVG-FR---GEV    36
CTLA4-Onmy-NP_001118005-Chr22-2-50495067                      ------------------------------------VWNALRVTQPYRVVS-HR---GEV    36
CTLA4-Icpu-XP_017324683-NoLOC-Chr6                            ------------------------------------LGHAFTVSQPYYAIG-GQ---GQV    33
```

```
ICOS-Hosa                                                        N-----------------------------------------GSANYEMFIFHN---GGV       38
ICOS-Mumu-XP_006496201                                           N-----------------------------------------GSADHRMFSFHN---GGV       38
ICOS-SwanGoose-XP_013027130                                      -----------------------ADTCSSR-PCK--NIDQLHVSDPQVMVEFKS---GNF       51
ICOS-Green_Anole-XP_062818357-LOC134293749                       IITHPVSESQKCSNLQDRGAKGGDGNCSSHTACNKDNLTEDLLSHSQKIDEFPK---ENF       80
ICOS-Turtle-XP_026503133-LOC112110999                            -----------------------TERCSSHRLCK--NIEQPTVSDPQVTVEFHN---GIF       52
ICOS-Alligator-XP_019347544-LOC102571456                         -----------------------AGQCLSCPLCK--DLGQPSFLDPQVKVEFHK---GIF       52
ICOS-Xenopustrop-XP_031749766-LOC100493997-Chr9-51,952,126       S-----------------------------------------KSYPKVVIASQD---GEP       36
ICOS-Polypterus-XP_039613787-LOC120531950                        ------------------------------------HLKAFKVVQSQRVPITSG---GMA       34
ICOS-MsPaddlefish-XP_041120674-LOC121323595                      ------------------------------------YLNAFQVTQPNLVAANSS---GFV       40
ICOS-Sterlet-XP_058889561-LOC117426591                           ------------------------------------YLNAFQVTQPHLVAANSS---GIA       34
ICOS-Gar-XP_015214822-LOC107078819-LG12-27245174                 ------------------------------------HLKAFQVSQPHRQTASTN---GTV       34
CD28-CTL4-Cami-XP_007888795-NoLOC                                ------------------------------------KMEELKVTQPKFLMANSSDSTGTI       43
CD28-CTLA4-Cami-XP_007888798-LOC103176829                        ------------------------------------GFQDIQVFQPRFLEANSD---GEA       45
CD28-CTLA4-SCyCa-XP_038642918-LOC119958447                       ------------------------------------PALALQIIQPRSILAESH----NA       40
CD28-CTLA4-SCyCa-XP_038646294-LOC119962407                       ------------------------------------GVANMSISTLQFLKANHN---GEA       41
CD28-CTLA4-SCyCa-XP_038642929-LOC119958457                       ------------------------------------D-AGLSISQPVLLEVEST---GEA       40
CD28-CTLA4-Leueri_XP_055494226_LOC129698880_Chr7_35060123-First  ------------------------------------S-VGLNVSQPRCLGVSNM---NEF       40
CD28-CTLA4-Leueri_XP_055494225_LOC129698879_Chr7_35020935-Second ------------------------------------GGPSRSSSKPHFVTANHR---GEV       41
CD28-CTLA4-Leueri_XP_055494224_LOC129698878_Chr7_34987938-Third  ------------------------------------PALAMKVTQPIKIIAERN----NV       41
CD28-CTLA4-Hemoc_XP_060684085_LOC132817598_Chr7_112525810-First  ------------------------------------DGAGLRVDQPRFLEVDST---GKA       41
CD28-CTLA4-Hemoc_XP_060684151_LOC132817669_Chr7_112615465-Second ------------------------------------------------------------       0
CD28-CTLA4-Hemoc_XP_060684086_LOC132817599_Chr7_112629451-Third  ------------------------------------PALALQVLQPRRILADSH----NV       40
CD28-CTLA4-Carcar_XP_041057727_LOC121285321_Chr12_125148145-Third ------------------------------------PALALQVIQPRLILAESH----NV      40
CD28-CTLA4-Carcar_XP_041057149_LOC121285085_Chr12_125379474-First ------------------------------------D-AGLKISQPVLLEVEST---GEA      40

CD28-Hosa-AJ517504                                               -NLSCKY-SYNLF-SREFRASLHKGLDSA---VEVCVVYGNYSQQLQ-------------       91
CD28-Mumu-BC064058                                               -SLSCRY-SYNLL-AKEFRASLYKGVNSD---VEVCVGNGNFTYQPQ-------------       78
CD28-SwanGoose-XP_013027129                                      -NLVCNY-SYNGT-GKEFRASLHKGTDSA---VEVCFISWNKTK-SS-------------       77
CD28-Green_Anole-XP_003223584-NoLOC                              -EISCNY-TSQKE-LNLLKISLLKGVEKR---VHVCSYSWNTTS-NA-------------       83
CD28-Xenopustrop-XP_017952834-NoLOC                              -TFPSNF----KD-NKESRVSLLRGVKKS---ITVCSGSFNSSYQSFE------------       76
CD28-Protopterus-XP_043916657-NoLOC                              -VLHCPF-SLPAN-TEEFKAVLHRNYSQG---SFICGSTFNITSKV--------------       73
CD28-Polypterus-XP_039613786-LOC120531949                        -IVPCSY--QNST-AKELKVQLLRGEKEE-----VCSVLI---PENKV------------       67
CD28-MsPaddlefish-XP_041120529-LOC121323502                      -ALECVYTLEGK--HKRVTLDVLKGSDQF--CTPVSVRQA----ENDSAFNS--------       76
CD28-Sterlet-XP_033899352-LOC117426134                           -TLECIYALEGK--HKHVTLDVLKGPDPF--CVPVSVRQI----ENGTVFNS--------       101
CD28-Gar-XP_015214827-LOC106878821-LG12-27177048                 -TVECKPETGNLT-GDEIKFVLFQNGRKE-----RCFGTK---KQNDTLHDS--------       86
CD28-Anguilla -XP_035264839-Chr3-si:dkey-1h24.6                  -SVRCPN----TT-GAEVKFELWKDKCKV-----GSVTRK----------QN--------      69
CD28-1-Esox-XP_010898663-Chr20-42937353-LOC105027959             -SIACPN----LT-GYDLRFHLLGSVHEN--SVTIHTTELNETKEQNRNHTGTN-----R       95
CD28-2-Esox-XP_010898665-Chr20-42928519-LOC105027960             -SIACPN----LT-GDDLRFHLLGSVHEN--SIKIHTTVLKKTKEQNRNHTGTN-----R       91
CD28-Esox-XP_010891418-Chr22-17852626-si:dkey-1h24.6             -PVCCPK-LKGKN-GEERLYTLLLGGSEI-----RNHSYD-----IGSNYS---------       79
CD28-Onmy-XP_036831245-LOC118964336-Chr3-1-83647331              -SISCPN----MT-GDDLRFHLFLN------QSLVHTTVL----KRNQNHTRNN-----Q       93
CD28-Onmy-XP_021464608-LOC110527567-Chr7-1-25301800              -SVSCPN-LTGKD-QEEMRFHLYLGLVEV-----GNHTHD-----NGHNHNSTE-----T       85
CD28-Onmy-NP_001118004-LOC100136274-Chr7-2-25315200              -SVRCPN-LTGKD-QEEMRFHLYLGLVEV-----GNHTHD-----SAHNHNSTE-----T       85
CD28-Onmy-XP_021427178-si:dkey-1h24.6-Chr18-27925782             -SVSCPN-LTGKD-QEEMRFHLYLGLVEV-----GNHTHD-----SAHNHNSTE-----T       87
CD28-Onmy-XP_036815388-LOC118943619-Chr22-1-50522004             -SISCPN----TT-GEDLSFHLSLN------QSLVHTTVL----ERNQIHTRTN-----Q       86
CD28-Icpu-XP_017313746-si:dkey-1h24.6-Chr26                      -SVPCPM----FT-AVEMTFKLHKGQNIT-----ATVTYV------NASHKS--------       78
CTLA4-Hosa-NP_005205                                             -SFVCEYASPGKA--TEVRVTVLRQADSQ--VTEVCAATYMMGNELT--FL-DD------       100
CTLA4-Mumu-NP_033973                                             -SFPCEYSPSHNT--DEVRVTVLRQTNDQ--MTEVCATTFTEKNTVG--FL-DY------       100
CTLA4-SwanGoose-XP_013027132                                     -SLVCKYKHIGNA--KEIRVTLLKQTGDQ--FTEICASTYTTEFKMF--SVEEV------       84
CTLA4-Green_Anole-XP_003223584-NoLOC                             -NFQCGFTNTEDA--SKLRITLLKQEGNE--SVQICALSFPLEEGSLFKTNEDG------       86
CTLA4-Xenopustrop-XP_012825450-NoLOC-54,453,384                  -MLVCDYRIHAKV--EEMRFRLLRKMGNQ--VKEICAFSYSTNYESV--TTGDA------       83
Protopterus-CTLA4-XP_043916244-LOC122792727                      -TVECRYNFNGTV--EEMRVSLFKRKDV---PLLVCAASFSTEFKPF--ETKGL------       103
CTLA4-Polypterus-XP_039610963-LOC120530608                       -QLACSFNHTGR--GQELRVTLLRGLAGK---DVVCKSSFQLPNTSL--ESDE-------       80
CTLA4-MsPaddlefish-XP_041119710-LOC121323053                     -RLRCNYNFTGK--GDEFRITLLRGISDQ---TIVCASSFHLPNTTF--ETKEG------       81
CTLA4-Sterlet-XP_033900514-LOC117426713                          -RLRCNYNFTGK--GEEFRITLFRGISDQ---TIVCASSFHLPNTTF--ETKEG------       81
CTLA4-Gar-XP_015214835-LOC102690085-LG12-27229939                -RLRCAFHFNGK--GEELRVTLYRGMGSD---SLVCASSFYLPNSTF--ETEGP------       83
Anguilla-CTLA4-XP_035266074-Chr3-NoLOC                           -RLRCSYAVRGS--PEEMRLTLYRGKFGD---DLVCQRTFNTTQSHF--QTDGP------       80
CTLA4-3-Esox-XP_012986927-Chr20-42914370-No                      -QFCCSYNHIGRHVPEELRVTLYRGLFGEEEEQEVCTSSFSHNQTLF--QTKGERGGVGA       125
CTLA4-Onmy-XP_036831244-LOC110505466-Chr3-2-83647331             -ELFCSYHHTGRHEPEELRVTLYRGAYREG-KQEVCTSSFTHNQTSF--QVEGV------       86
CTLA4-Onmy-NP_001118005-Chr22-2-50495067                         -ELFCSYHHTGRNEPEELRITLYRGMYGEQEEQKVCTSSFTHNNTAF--QVEGE------       87
CTLA4-Icpu-XP_017324683-NoLOC-Chr6                               -SFHCTINTKSQ--PGEMQVSVYKGMYGE---KRICSAYVNISYPHI--ATNGG------       79
ICOS-Hosa                                                        -QILCKYPDIVQ----QFKMQLLKGGQ------ILCDLTKTKGSGNTVSIKSLK------       81
ICOS-Mumu-XP_006496201                                           -QISCKYPETVQ----QLKMRLFRERE------VLCELTKTKGSGNAVSIKNPM------       81
ICOS-SwanGoose-XP_013027130                                      -KFIFHNPKNVS----EFSMTLFKGHE----KKEICALHVSK--EKAIPKSNVT------       94
ICOS-Green_Anole-XP_062818357-LOC134293749                       -TFHCPLPSRVK----AFRMRLLKGQR------EVCVFYKDN--GQ-INCTCKE------       120
ICOS-Turtle-XP_026503133-LOC112110999                            -KFNFSDPKNVT----EFSMTLLKGHE----RQEICAFHMEN--GKSIPETNSS------       95
ICOS-Alligator-XP_019347544-LOC102571456                         -QFNISSPKNVT----DFSITLLKGRE----RQSICALHVTE--GKAVPENGSS------       95
ICOS-Xenopustrop-XP_031749766-LOC100493997-Chr9-51,952,126       CLLCHHVPSTDS----KFNLTLMRGNK----KQEVCMVYTDGKNTSFYSWNDNP------       82
ICOS-Polypterus-XP_039613787-LOC120531950                        -SLSCSYEIPLSE-AKEVHVFLHRVLHND---TTSCDKSTGTSVDPF-------------       76
ICOS-MsPaddlefish-XP_041120674-LOC121323595                      -IMTCDYKYS-KE-IIELKTYLLRARHND---TISCETSVNDSKKGI-------------       81
ICOS-Sterlet-XP_058889561-LOC117426591                           -ILTCDYKYS-KQ-IVELKIYLLRARHND---TISCETSVNESKKGS-------------       75
ICOS-Gar-XP_015214822-LOC107078819-LG12-27245174                 -TFTCSYTSPTKN-FSDVQVHLMRRVHND---SLPCAKLRNDIQKSV-------------       76
CD28-CTL4-Cami-XP_007888795-NoLOC                                -TMVCGYIFTGT-LSEEFRITIYKGRIVK--EAEVCVASFNSSMLPF--EKNQT------       91
CD28-CTLA4-Cami-XP_007888798-LOC103176829                        -TLVCSHDHTGHA---VELQVTIRKT--N--QEEVCNGTYNSTTKPF--QTRAR------       89
CD28-CTLA4-SCyCa-XP_038642918-LOC119958447                       -TFACAYNNTGEGPLEEFRISVHKGEIGR--DTEVCAFFFNTTINYL--ETTMA------       89
CD28-CTLA4-SCyCa-XP_038646294-LOC119962407                       -SLVCALRSTEAG--EELQVVLYKGEQSQ--RTEVCTASFTNGTME-PSERKDL------       89
CD28-CTLA4-SCyCa-XP_038642929-LOC119958457                       -TLQCLDSCDEKS---ELKLTILKGK--D--KIVICTGTINASTHSV--DSQGL------       84
CD28-CTLA4-Leueri_XP_055494226_LOC129698880_Chr7_35060123-First  -ALECEHSSDDVA---ELALTILKGL--N--KSEVCRGVISSTELYI--TSYGQ------       84
CD28-CTLA4-Leueri_XP_055494225_LOC129698879_Chr7_35020935-Second -TLLCGLNGTMIG--EEFQVVLHQGAVHI--TTVVCIVFFSNWTIQ-PSKTKDR------       89
CD28-CTLA4-Leueri_XP_055494224_LOC129698878_Chr7_34987938-Third  -TLKCVYNITGDGKAQVFRVTVYKSNEEK--NSEVCAASFNNSMKPF--EKRDV------       90
CD28-CTLA4-Hemoc_XP_060684085_LOC132817598_Chr7_112525810-First  -TLQCNHSWDEKT---ELKLTILKER--D--SREVCIGRTNSSVHYV--NSTEL------       85
CD28-CTLA4-Hemoc_XP_060684151_LOC132817669_Chr7_112615465-Second ---------------------------------------------------MGL------       3
CD28-CTLA4-Hemoc_XP_060684086_LOC132817599_Chr7_112629451-Third  -TLECAYDTTGDGPFEQFGIIIYKGEPTN--DTEVCAMFFNNSANHF--KTATA------       89
CD28-CTLA4-Carcar_XP_041057727_LOC121285321_Chr12_125148145-Third -TLKCAYDNTRDEPLEQFRIHILKGERGA--NIKVCDLYFDISKNKL--PN--D------      87
CD28-CTLA4-Carcar_XP_041057149_LOC121285085_Chr12_125379474-First -TLECAHNYDENT---ELKLTILKGR--V--ENVACIGTTNSSVYSV--NSTGL------      84

CD28-Hosa-AJ517504                                               VYSKTGFNCDG-KLGN-----ESVTFYLQNLYVNQTDIYFCKIEVMYPP-PYLDNEKSNG       144
CD28-Mumu-BC064058                                               FRSNAEFNCDG-DFDN-----ETVTFRLWNLHVNHTDIYFCKIEFMYPP-PYLDNERSNG       131
CD28-SwanGoose-XP_013027129                                      SNSNKEFNCQG-TFYE-----DKVIFNLWNMNANQTDIYFCKIEAMYPP-PYVYNEKSNG       130
CD28-Green_Anole-XP_003223584-NoLOC                              TKTERDFNCWV-DFAKK---QNKTTFHLQNLHVNQTDIYICKIEDLYPP-PYEASVSHNG       138
CD28-Xenopustrop-XP_017952834-NoLOC                              SNAYQSIRCIG-IPTE-----NKITFHLSGLTENHTDIYFFRKEDMYPP-PYTCY-QDNG       128
CD28-Protopterus-XP_043916657-NoLOC                              TSPHNGSQCIV-TADN-----TTLSFFLSNLTPKDTAVYECIIEVMYPP-PYQKK-YGNG       125
CD28-Polypterus-XP_039613786-LOC120531949                        --NVTEPGACR-IEVFN---H-SVNFHLSSLRPNDSDLYSCDMLTLYPP-PFLKI-MGQK       118
CD28-MsPaddlefish-XP_041120529-LOC121323502                      SA---RFNHCE-GMDKG---D-RVVFKLSSLQEKDTDMYYCQLNILYPP-PFRNP-KGTG       126
CD28-Sterlet-XP_033899352-LOC117426134                           SV---SFKHCE-GMDKG---D-RVIFKLSNLQEKDTDMYYCQLNIMYPP-PFRSP-KGNG       151
CD28-Gar-XP_015214827-LOC106878821-LG12-27177048                 SSPEACPVNF-----GL---S-QAYFTLSQLQTHDTGIYSCDIEVLYPP-PYRKI-NGST       135
CD28-Anguilla-XP_035264839-Chr3-si:dkey-1h24.6                   QTETEGAVDFR-DQDQN---T-TARFVLSQVDRNSTGIFACNAERLYPP-PYLKS-PGVE       122
CD28-1-Esox-XP_010898663-Chr20-42937353-LOC105027959             QE---TFVQFG-QVEPA---LNQNLSNRYVFTVNGTGLYSCKAEKLWPP-PYKE--DSVE       145
CD28-2-Esox-XP_010898665-Chr20-42928519-LOC105027960             QE---SFVQFG-QVEPA---LNQNLSNSYVFTVNGTGLYSCKAERMWPP-PYKE--DTVE       141
CD28-Esox-XP_010891418-Chr22-17852626-si:dkey-1h24.6             -ETVKEGLRIQ-VNKME---Y-TFKFVLSGITTQQAGVYTCKVQNTYPP-PVEDV-KEMC       131
CD28-Onmy-XP_036831245-LOC118964336-Chr3-1-83647331              SD---TWVQFY-LAEQN---LNHNLTNRYVFTVNGTGLYICRAERLWSP-PYKV--DCVH       143
CD28-Onmy-XP_021464608-LOC110527567-Chr7-1-25301800              VSPVGEGLGLR-VNEQD---H-TVSFVLSGMTTERAGVYTCEGQPMCPP-PIEKL-PDET       138
CD28-Onmy-NP_001118004-LOC100136274-Chr7-2-25315200              VSPVGEGLGLR-VNEQD---H-TVSFVLSGMTTERAGVYTCEGYPMYPP-PIEKV-PDEP       138
CD28-Onmy-XP_021427178-si:dkey-1h24.6-Chr18-27925782             VSPVGEGLGLR-VNEQD---H-TVSFVLSGMTTERAGVYTCEGQPMYPP-PIEKV-QDET       140
CD28-Onmy-XP_036815388-LOC118943619-Chr22-1-50522004             TD---TWAQFY-LVDQN---RNQNLINRYVFTVNSTGLYTCIAERMRSPLPYQE--DSVQ       137
CD28-Icpu-XP_017313746-si:dkey-1h24.6-Chr26                      SPEPLGSKMYH-SVKDD---N-TTHFLLYNVTMDATGLYTCTAEKSYPP-PMVTI-QEEP       131
CTLA4-Hosa-NP_005205                                             ------SICTG-TSSGN-----QVNLTIQGLRAMDTGLYICKVELMYPP-PYYLG-IGNG       146
CTLA4-Mumu-NP_033973                                             ------PFCSG-TFNES-----RVNLTIQGLRAVDTGLYLCKVELMYPP-PYFVG-MGNG       146
CTLA4-SwanGoose-XP_013027132                                     ------IQCRV-SPSRN-----NVTLTLTGLQVNDTGLYVCKMERMYPP-PYFMN-KGNG       130
CTLA4-Green_Anole-XP_003223584-NoLOC                             ------IQCQI-HPGRE-----SVNITLWGLKATDAGLYVCQMEWIYPP-PYYSV-LGSG       132
CTLA4-Xenopustrop-XP_012825450-NoLOC-54,453,384                  ------IQCEG-EPGPN-----NVTLHLSGMQMSDTGMYICKLDIMYPP-PYRTT-EGNG       129
Protopterus-CTLA4-XP_043916244-LOC122792727                      ------LQCKG-QPGPA-----NVSITVSGLKAADTGTYICKMEIMYPP-PYRVR-MGNG       149
CTLA4-Polypterus-XP_039610963-LOC120530608                       -----PLSCQG-NASAN-----RLDLTVPGLSGADTDFYRCKVEILYPP-PYRLG-LGSG       127
CTLA4-MsPaddlefish-XP_041119710-LOC121323053                     -----VFSCQG-EASRN-----SVDLTISGMSSSDTDIYKCRVEIMYPP-PYRQR-VGKG       128
CTLA4-Sterlet-XP_033900514-LOC117426713                          -----LFSCQG-EASRN-----SVDLTISGMSSFDTDIYKCRVEIMYPP-PYRQR-VGNG       128
CTLA4-Gar-XP_015214835-LOC102690085-LG12-27229939                -----VV-CRG-DASKD-----GVDLTVSGLQGADTDLYRCSLEIMYPP-PYRQR-YGNG       129
Anguilla-CTLA4-XP_035266074-Chr3-NoLOC                           -----VVRCRG-ELTPG-----GVDLAVSGLRGEDTDIYRCQLEVLYPP-PYLMK-YGNG       127
CTLA4-3-Esox-XP_012986927-Chr20-42914370-No                      GDRETPVLCRG-QVEAG-----MVNLTISGLRGNDTDLYRCAIEVMYPP-PYLRV-FGNG       177
CTLA4-Onmy-XP_036831244-LOC110505466-Chr3-2-83647331             --GE--VRCRG-QLGPG-----RVNLTISGLQGNDTDLYYCGIEVMYPP-PYLRR-FGNG       134
CTLA4-Onmy-NP_001118005-Chr22-2-50495067                         --GERKVRCRG-QLRPG-----KVNLTISGLRGNDTDLYRCAIEVLYPP-PYLRT-FGNG       137
CTLA4-Icpu-XP_017324683-NoLOC-Chr6                               ------VYCRG-NVSRG-----KVDLTIFGLRGEDTDLYRCQIDIIFPP-PYISK-FGNG       125
ICOS-Hosa                                                        -------FCHS-QLSNN-----SVSFFLYNLDHSHANYYFCNLSIFDPP-PFKV--TLTG       125
ICOS-Mumu-XP_006496201                                           -------LCLY-HLSNN-----SVSFFLNNPDSSQGSYYFCSLSIFDPP-PFQER-NLSG       126
ICOS-SwanGoose-XP_013027130                                      -------YCQA-EHSNT-----STTFILTNLERKHIDTYTYCLEMFLPP-PYIDC-RLKE       139
```

```
ICOS-Green_Anole-XP_062818357-LOC134293749                              -------FCDV-MPHDG-----RVSFQLKNLNSKHSDTYTCCLEIVS-P-IFSRC-KAGE          164
ICOS-Turtle-XP_026503133-LOC112110999                                   -------YCEP-VHSGN-----STSFILRNLESKHTGIYLCCLEILLPA-PYIDC-RVNE          140
ICOS-Alligator-XP_019347544-LOC102571456                                -------YCQP-HYFGN-----NTSFILQNLQRNHSDIYIYCLQILLPP-PYIDC-KVDE          140
ICOS-Xenopustrop-XP_031749766-LOC100493997-Chr9-51,952,126              -------KCNWTKSNDS-----SISFTLSNFDIKHTDNYTCEIRIFYPP-PFRSI-IINE          128
ICOS-Polypterus-XP_039613787-LOC120531950                               -------NCAM-SSQEQNSTHSSFTLTIENLKKDDEDVYICKVEKRKPF-PYEDG-VGVG          126
ICOS-MsPaddlefish-XP_041120674-LOC121323595                             -------HCLM-ERYNN-----SVALTITLFNVSDADLYVCRVEVRSPP-PFVYG-EGTG          126
ICOS-Sterlet-XP_058889561-LOC117426591                                  -------LCLM-ECYEN-----SVTLTISRLNVSDADLYVCRVEIRSPP-PFVYA-EGTG          120
ICOS-Gar-XP_015214822-LOC107078819-LG12-27245174                        -------SCLM-SEKEG-----SVTFNIFNFTHNDTDLYVCRVHRKLPL-PFVSR-DGDG          121
CD28-CTL4-Cami-XP_007888795-NoLOC                                       ------FHCLG-KPSAD-----KVSITLSGLNMLDTNMYYCKVTKMHPP-PIINS-IGNG          137
CD28-CTLA4-Cami-XP_007888798-LOC103176829                               ------FHCWI-QPGEG-----NVSVTIYGFNSSAIDYYFCKLERVHPP-VYQSG-IGNG          135
CD28-CTLA4-SCyCa-XP_038642918-LOC119958447                              ------LHCQG-WSRNG-----SVSITISEINTSQSDLYICQIEKMHPP-PYIKS-NGNG          135
CD28-CTLA4-SCyCa-XP_038646294-LOC119962407                              ------FRCQV-SLSQN-----NVSITVSGLNVTDTDRYFCQVSKTHPP-PYFES-PGQW          135
CD28-CTLA4-SCyCa-XP_038642929-LOC119958457                              ------LQCQI-NQKQK-----ILSATLCGFNSSFIDNYFCQIEKLYPP-PYEKK-QGDG          130
CD28-CTLA4-Leueri_XP_055494226_LOC129698880_Chr7_35060123-First         ------LNCQF-QRIPN-----KISVIISGLHSSMIDIYFCKIELLYPP-PFESS-EGSG          130
CD28-CTLA4-Leueri_XP_055494225_LOC129698879_Chr7_35020935-Second        ------LRCEF-QYSQS-----NVSVTIKDLNVTDTDRYICNTRKTYPP-PYLTI-SGQW          135
CD28-CTLA4-Leueri_XP_055494224_LOC129698878_Chr7_34987938-Third         ------LHCIG-QPRRN-----SISIQFIGMNMSHSDWYFCKVEKMFPL-PYTQA-TGNG          136
CD28-CTLA4-Hemoc_XP_060684085_LOC132817598_Chr7_112525810-First         ------LHCQI-NRTSG-----SIYVTFYGFNSSLIDNFFCKIEKLYPP-PYEGK-QGNG          131
CD28-CTLA4-Hemoc_XP_060684151_LOC132817669_Chr7_112615465-Second        ------LHCKV-SLSRN-----NVSITIKGLNATDTDRYICVVLNTHPP-PFYES-SGQW          49
CD28-CTLA4-Hemoc_XP_060684086_LOC132817599_Chr7_112629451-Third         ------PNCQA-QIQNG-----SVSVTLSGLNTSHSDLYICHMAKIHPP-PYIEG-KGDG          135
CD28-CTLA4-Carcar_XP_041057727_LOC121285321_Chr12_125148145-Third       ------QSCQG-QPRNG-----SVLITFNGLNTSQSDLYICQIQKIHPP-PYITS-FGNG          133
CD28-CTLA4-Carcar_XP_041057149_LOC121285085_Chr12_125379474-First       ------LQCRI-TRKRY-----IVSVTLCGLNSSLIDNYFCKIEKLYPP-PYEGV-KGNG          130
                                                                                                          :

CD28-Hosa-AJ517504                                                      TIIHVKGKHL-----------------CPSP-----------L--FPGPS--------            164
CD28-Mumu-BC064058                                                      TIIHIKEKHL-----------------CHTQ-----------S-----SP--------            148
CD28-SwanGoose-XP_013027129                                             TVIHVKETPI-----------------QTQ---------------EPQSA--------            148
CD28-Green_Anole-XP_003223584-NoLOC                                     TLIHVKGVSE-----------------VPVC-----------Q--CHQHG--------            158
CD28-Xenopustrop-XP_017952834-NoLOC                                     TVIHVKELVA-----------------PVKD-----------------PLP--------           145
CD28-Protopterus-XP_043916657-NoLOC                                     TLIDVNGVPP-----------------SPVP-----------C--V--PN--------            143
CD28-Polypterus-XP_039613786-LOC120531949                               TMVCVIDKAE---------------EKICTQC--------PDVRS----DGP--------          143
CD28-MsPaddlefish-XP_041120529-LOC121323502                             TMIIFQRKNS-----------------TETLC--------S---L----TSD--------          146
CD28-Sterlet-XP_033899352-LOC117426134                                  TLIIFQRKNN----------------STEPPC--------SPTGT----SKD--------          175
CD28-Gar-XP_015214827-LOC106878821-LG12-27177048                        TVLIVDENEC-----------------KEPI--------TPCGS----NFP--------           157
Anguilla-CD28-XP_035264839-Chr3-si:dkey-1h24.6                          NMVVVNE-LP-----------------QVRA--------CPL------EKP--------           141
CD28-1-Esox-XP_010898663-Chr20-42937353-LOC105027959                    TLLI-EDEQC---------------SGHQDPL--------AKQNC-ADDPRR--------          172
CD28-2-Esox-XP_010898665-Chr20-42928519-LOC105027960                    TMLI-EG-EC---------------QNHQVPQ--------ENPIC-PEDLKP--------          167
CD28-Esox-XP_010891418-Chr22-17852626-si:dkey-1h24.6                    QTLVLVQDYQ-----------------GPA-----------GCEG---HG--------            150
CD28-Onmy-XP_036831245-LOC118964336-Chr3-1-83647331                     TLLI-TEKQC---------------LKPHDPLTT----------C-VQDHNP--------          168
CD28-Onmy-XP_021464608-LOC110527567-Chr7-1-25301800                     QTLVLVEALQ-----------------CQEM--------KIGVCAG-----------             159
CD28-Onmy-NP_001118004-LOC100136274-Chr7-2-25315200                     QTLVLVEAFQ-----------------CQAG-----------GCVGPRVHG--------           161
CD28-Onmy-XP_021427178-si:dkey-1h24.6-Chr18-27925782                    QTVVLVEAYQ-----------------CQAG--------KPVGCVGSRVDG--------           166
CD28-Onmy-XP_036815388-LOC118943619-Chr22-1-50522004                    TLLI-EEKQC---------------LTHRDPLANQDQDPLANQNC-AEVSKP--------          172
CD28-Icpu-XP_017313746-si:dkey-1h24.6-Chr26                             QTIVIVENHG-----------------CVQN--------SQRMD----PSA--------           153
CTLA4-Hosa-NP_005205                                                    TQIYVIDPEP-----------------C-------------PD-----SD--------            161
CTLA4-Mumu-NP_033973                                                    TRIYVIDPEP-----------------C-------------PD-----SD--------            161
CTLA4-SwanGoose-XP_013027132                                            TQLYVIDPEP-----------------C-------------PD-----TA--------            145
CTLA4-Green_Anole-XP_003223584-NoLOC                                    TQLFLLERDA-----------------C-------------PA-----VY--------            147
CTLA4-Xenopustrop-XP_012825450-NoLOC-54,453,384                         TLIYVSDLMS-----------------ECAQS--------IEPPE----FI--------           151
Protopterus-CTLA4-XP_043916244-LOC122792727                             TLVHVFDIEP-----------------C-------------ED-----PF--------            164
CTLA4-Polypterus-XP_039610963-LOC120530608                              TLIYVAERPP-----------------CPAK--------EHSM----------D--W--           147
CTLA4-MsPaddlefish-XP_041119710-LOC121323053                            TVIHIPEIQT-----------------CPKI--------ESNM----------E--W--           148
CTLA4-Sterlet-XP_033900514-LOC117426713                                 TVIYIPEIQT-----------------CPKI--------ESSM----------E--W--           148
CTLA4-Gar-XP_015214835-LOC102690085-LG12-27229939                       TIIYIPEETK-----------------CADP--------VVAAE-----TDK-ENIL--           155
Anguilla-CTLA4-XP_035266074-Chr3-NoLOC                                  TIVYIPEKTD-----------------CPTP--------ASQAQ-----DEP-QSTQ--           153
CTLA4-3-Esox-XP_012986927-Chr20-42914370-No                             TLLYIPEEPD-----------------CPAL--------EPKRR-----DDS-GSLW--           203
CTLA4-Onmy-XP_036831244-LOC110505466-Chr3-2-83647331                    TLLFIPEEPG-----------------CSLP--------EAQRT-----SD-MGETS--           160
CTLA4-Onmy-NP_001118005-Chr22-2-50495067                                TLLYITEEPG-----------------CFTP--------EAQRR-----DDVTGETS--           164
CTLA4-Icpu-XP_017324683-NoLOC-Chr6                                      TLVHIPDSPD-----------------CPPE--------HTQAR-----IQEIPEITYD           154
ICOS-Hosa                                                               GYLHIYESQ--------------------------------LC-----CQ--------            138
ICOS-Mumu-XP_006496201                                                  GYLHIYESQ--------------------------------LC-----CQ--------            139
ICOS-SwanGoose-XP_013027130                                             TYLYIQDKE-----------------------------DCIS-----LG--------             154
ICOS-Green_Anole-XP_062818357-LOC134293749                              KHLYIEDPAE-----------------SYSA--------KPCLF-----SE--------           185
ICOS-Turtle-XP_026503133-LOC112110999                                   TYLYIHDSE------------------------------TCFV-----SE--------            155
ICOS-Alligator-XP_019347544-LOC102571456                                TYLFIHDKE------------------------------ECTL-----PR--------            155
ICOS-Xenopustrop-XP_031749766-LOC100493997-Chr9-51,952,126              TYVYIHDLQQ-----------------CGS-----------GM-----QE--------            145
ICOS-Polypterus-XP_039613787-LOC120531950                               TLVLIAAQLP----------------NECDKV----------LEM-----QP-----D--          148
ICOS-MsPaddlefish-XP_041120674-LOC121323595                             TILYVAETSQ----------------IPRSEY----------TD-----FN-----W--           147
ICOS-Sterlet-XP_058889561-LOC117426591                                  TIVYVAETSQ----------------IPLPQC----------TD-----FN-----W--           141
ICOS-Gar-XP_015214822-LOC107078819-LG12-27245174                        TLLVFPEIKT----------------EDTKPS----------IC-----LD-----D--           142
CD28-CTL4-Cami-XP_007888795-NoLOC                                       TVFYIQAKEDYCKENCTSGETVKQEPGNC--------------NE-----FT--------          170
CD28-CTLA4-Cami-XP_007888798-LOC103176829                               TLIYIAGKKH---------------PSSAALD--------LKDQL-----FQV-------          160
CD28-CTLA4-SCyCa-XP_038642918-LOC119958447                              TWIFISPNTT-------------EIKRC--------------GQ-----FT--------           154
CD28-CTLA4-SCyCa-XP_038646294-LOC119962407                              TIIYINRATN-----------------CHAG--------QESSD-----AH--------           156
CD28-CTLA4-SCyCa-XP_038642929-LOC119958457                              TIIYTRN------------------RNC--------------SQ-----LLL-------           145
CD28-CTLA4-Leueri_XP_055494226_LOC129698880_Chr7_35060123-First         TFIYPSSKD-----------------MKC--------------PR-----FSF-------          147
CD28-CTLA4-Leueri_XP_055494225_LOC129698879_Chr7_35020935-Second        TIIHVNRVAS-----------------CPPE--------VETGH-----WL--------           156
CD28-CTLA4-Leueri_XP_055494224_LOC129698878_Chr7_34987938-Third         TWIFIKPDVE--------ALSVPSEKKRC--------------PQ-----VT--------          161
CD28-CTLA4-Hemoc_XP_060684085_LOC132817598_Chr7_112525810-First         TIMFISSKEN---------------CEKC--------------FQ-----FVM-------          150
CD28-CTLA4-Hemoc_XP_060684151_LOC132817669_Chr7_112615465-Second        TIIYINQATS-----------------CPVD--------EGASN-----GF--------           70
CD28-CTLA4-Hemoc_XP_060684086_LOC132817599_Chr7_112629451-Third         TCIFIAPEMP---------------EAKPC--------------PE-----FT--------         154
CD28-CTLA4-Carcar_XP_041057727_LOC121285321_Chr12_125148145-Third       TWIFISPDRV--------------EMKPC--------------GQ-----FT--------          152
CD28-CTLA4-Carcar_XP_041057149_LOC121285085_Chr12_125379474-First       TIIYTRS------------------EKC--------------SQ-----FLL-------           145
                                                                          .

CD28-Hosa-AJ517504                                                      -----KPFWVLVVVGGVLACYSLLVTVAFIIFWVR-------------------------          194
CD28-Mumu-BC064058                                                      -----KLFWALVVVAGVLFCYGLLVTVALCVIWTN-------------------------          178
CD28-SwanGoose-XP_013027129                                             -----ISYWVMVAVTGLLAFYSILITAIFISYWQK-------------------------          178
CD28-Green_Anole-XP_003223584-NoLOC                                     -----LEYMLMIAAIIIFIVYSVTVTAACYYCWRK-------------------------          188
CD28-Xenopustrop-XP_017952834-NoLOC                                     -----QPQWPLLVAIGVMAAYSLTITAAFFYILKK-------------------------          175
CD28-Protopterus-XP_043916657-NoLOC                                     -----DISWIYLAVILVLGIYGIFMTGLMIICWKK-------------------------          173
CD28-Polypterus-XP_039613786-LOC120531949                               -----LQLALWI-LLAFLALYSLITSIALFVSYVK-------------------------          172
CD28-MsPaddlefish-XP_041120529-LOC121323502                             -----SLFWILITAIAVIVLYGVIISLLLYITCYK-------------------------          176
CD28-Sterlet-XP_033899352-LOC117426134                                  -----SIFWVLNAVIVMLAIYGMTVSLLIYITCYK-------------------------          205
CD28-Gar-XP_015214827-LOC106878821-LG12-27177048                        -----LTLVLMI-AGCVILLYSLVITVIMAICYIK-------------------------          186
Anguilla-CD28-XP_035264839-Chr3-si:dkey-1h24.6                          -----TEAWIWA-ALGVAAAYSLIMTGIAVGLWHY-------------------------          170
CD28-1-Esox-XP_010898663-Chr20-42937353-LOC105027959                    -----LLLWLL-VACGLLTVYSIIATT---IIWRK-------------------------          198
CD28-2-Esox-XP_010898665-Chr20-42928519-LOC105027960                    -----LLLWLLLVGCGVLFFYSVIITIITIFVTRK-------------------------          197
CD28-Esox-XP_010891418-Chr22-17852626-si:dkey-1h24.6                    -----DVGWIWKPLFWINLVYGLAVTIIALACCVR-------------------------          180
CD28-Onmy-XP_036831245-LOC118964336-Chr3-1-83647331                     -----LLLCLLLVGCGVLLLYSTILTITVVLIWRK-------------------------          198
CD28-Onmy-XP_021464608-LOC110527567-Chr7-1-25301800                     ------SAWTVVLGFWVTIIYGLAVTVIAFAIRLR-------------------------          188
CD28-Onmy-NP_001118004-LOC100136274-Chr7-2-25315200                     -----VPVWAWMLGFWVTTIYGLVVTVFAFVIWLR-------------------------          191
CD28-Onmy-XP_021427178-si:dkey-1h24.6-Chr18-27925782                    -----VPVWAWVLGFWITIIYGLAVTVIAFVIWLR-------------------------          196
CD28-Onmy-XP_036815388-LOC118943619-Chr22-1-50522004                    -----LPLCPLLVACGFL-LYSIIITIIAIVLWRK-------------------------          201
CD28-Icpu-XP_017313746-si:dkey-1h24.6-Chr26                             -----SHLPLWV-GFGILSVYCLIITCIALSLRFR-------------------------          182
CTLA4-Hosa-NP_005205                                                    -----FLLWILAAVSSGLFFYSFLLTAVSLSKMLK-------------------------          191
CTLA4-Mumu-NP_033973                                                    -----FLLWILVAVSLGLFFYSFLVTAVSLSKMLK-------------------------          191
CTLA4-SwanGoose-XP_013027132                                            -----IYLWVLGATASGFFLYSFIISAVVVGKAIQ-------------------------          175
CTLA4-Green_Anole-XP_003223584-NoLOC                                    -----PYFWISVMIASGLLGYSILITIYMKTKVLR-------------------------          177
CTLA4-Xenopustrop-XP_012825450-NoLOC-54,453,384                         -----LDQRILLVVCLVMFLYSMFITAVLLCG--K-------------------------          179
Protopterus-CTLA4-XP_043916244-LOC122792727                             -----LYILIPSTVAAGLLLHSIVMTLAFCIYKLK-------------------------          194
CTLA4-Polypterus-XP_039610963-LOC120530608                              ---VFMAVPVTVVVCTVIVIISILVYRTLNRK-AN-------------------------          178
CTLA4-MsPaddlefish-XP_041119710-LOC121323053                            ---IFVAVAGCIIACTVIIIAAILVIRICQQN-WK-------------------------          179
CTLA4-Sterlet-XP_033900514-LOC117426713                                 ---IFVAVSGCIIACTVIIIAAILVIRICQQN-RK-------------------------          179
CTLA4-Gar-XP_015214835-LOC102690085-LG12-27229939                       ---FFLPVSVLAFIIIMTI--LILTYRILSLKYRK-------------------------          185
Anguilla-CTLA4-XP_035266074-Chr3-NoLOC                                  ---VVLPIAVMAIFIIFIIAIISIYMFFNTSQRK-------------------------           185
CTLA4-3-Esox-XP_012986927-Chr20-42914370-No                             ---TRPPLVSVAWVSLLLMVVSNVAFVLYQVLQRK-------------------------          235
CTLA4-Onmy-XP_036831244-LOC110505466-Chr3-2-83647331                    ---VRLALAGLV-AANILMVISVVAFLLYQVLQRK-------------------------          191
CTLA4-Onmy-NP_001118005-Chr22-2-50495067                                ---VRLPLAGLA-AVLIV-ISAIAILLVHQVLQRK-------------------------          194
CTLA4-Icpu-XP_017324683-NoLOC-Chr6                                      QTLSFPNIVLSAILIT-TTITLILQVMKMILTQRK-------------------------          188
ICOS-Hosa                                                               -----LKFWLPIGCAAFVVVCILGCILICWLTKK--------------------------          167
ICOS-Mumu-XP_006496201                                                  -----LKLWLPVGCAAFVVVLLFGCILIIWFSKK--------------------------          168
ICOS-SwanGoose-XP_013027130                                             -----IMSWVTIGLIVFAMISCVCCVAACRLRNK--------------------------          183
ICOS-Green_Anole-XP_062818357-LOC134293749                              -----LNSWILVGFTAFCLVACIFCLIVCSFRNV--------------------------          214
```

```
ICOS-Turtle-XP_026503133-LOC112110999                                 -----MTSWMLIGITAFSMVSCICCIVACCLRKK--------------------------   184
ICOS-Alligator-XP_019347544-LOC102571456                              -----FLSWIIIGLTAFFMTSCICCIIACCLRNK--------------------------   184
ICOS-Xenopustrop-XP_031749766-LOC100493997-Chr9-51,952,126            -----FIIWILTGLAVFLFLCCIFTFCL-WIQNRF---------------------RRKC   178
ICOS-Polypterus-XP_039613787-LOC120531950                             -----PLLVTVAVIAGILLLYSVAISVSWGLVE---------------------------   176
ICOS-MsPaddlefish-XP_041120674-LOC121323595                           -----PVVWIIAGVSGLLLLYSIIITLTCLHAK---------------------------   175
ICOS-Sterlet-XP_058889561-LOC117426591                                -----PVVWINAGVSGLLLLYSIVITLTCLCLK---------------------------   169
ICOS-Gar-XP_015214822-LOC107078819-LG12-27245174                      -----SLLWILVGVAAFSIIYSISVTFACLKLKVSLDEEMDLSEAHSNGQVHLQAGDSPS   197
CD28-CTL4-Cami-XP_007888795-NoLOC                                     -----QATVIVLVLLLVFLLYSIVITCAHCMMKAS-------------------------   200
CD28-CTLA4-Cami-XP_007888798-LOC103176829                             ----TLLLAVFAFI---LLIYSIAITFVHFHSKR--------------------------   187
CD28-CTLA4-SCyCa-XP_038642918-LOC119958447                            -----QTTLIVLAFTAIFILYSVFITCLHWMLKKN-------------------------   184
CD28-CTLA4-SCyCa-XP_038646294-LOC119962407                            -----LLTVIFIVLAILLFLYSVSVTVMCCRHQMK-------------------------   186
CD28-CTLA4-SCyCa-XP_038642929-LOC119958457                            ----FLTMGILGGLTLFSMIYSIVLTLMSCRPKK--------------------------   175
CD28-CTLA4-Leueri_XP_055494226_LOC129698880_Chr7_35060123-First       ----LLIMGILIPVLTLCMINIFFLTTKVSNKANFT------------------------   179
CD28-CTLA4-Leueri_XP_055494225_LOC129698879_Chr7_35020935-Second      -----PLTAFFTSLAALLLLYSVSVTVVYCRHKMK-------------------------   186
CD28-CTLA4-Leueri_XP_055494224_LOC129698878_Chr7_34987938-Third       -----EAALIILALASLFLLYSIVVTYLHWVLKNK-------------------------   191
CD28-CTLA4-Hemoc_XP_060684085_LOC132817598_Chr7_112525810-First       ----LLIMGILSFLTLSSMIYSIVLTSKICRVKK--------------------------   180
CD28-CTLA4-Hemoc_XP_060684151_LOC132817669_Chr7_112615465-Second      -----LPTIILIVLAALLFLYSASLTVLYFKHKMK-------------------------   100
CD28-CTLA4-Hemoc_XP_060684086_LOC132817599_Chr7_112629451-Third       -----QSFMIVLVIAALFMFSSIFISCIHWPLGTT-------------------------   184
CD28-CTLA4-Carcar_XP_041057727_LOC121285321_Chr12_125148145-Third     -----QTTLIILVLTAFFMLYSVLITCLHWMLKKN-------------------------   182
CD28-CTLA4-Carcar_XP_041057149_LOC121285085_Chr12_125379474-First     ----LLSMGILGSMTLSCMIYSIVLTLMNCRAKK--------------------------   175


CD28-Hosa-AJ517504                                                    ----SKRSRLLHSDYMNMT--------PRRPGP-TRKHYQPYAPPR-DFAAYRS------   234
CD28-Mumu-BC064058                                                    ----SRRNRLLQSDYMNMT--------PRRPGL-TRKPYQPYAPAR-DFAAYRP------   218
CD28-SwanGoose-XP_013027129                                           ----SKKNRYHQSDYMNMT--------PRHPPYQKNKGYPPYAPTR-DYTAYRSWQP---   222
CD28-Green_Anole-XP_003223584-NoLOC                                   ----AKKNRIVRNDYFNMT--------PWQSNGVKKRQLPQSVPAR-NYTAYRSWEP---   232
CD28-Xenopustrop-XP_017952834-NoLOC                                   ----GRRTRVLQSEYINVV--------PRRPKC-----HQPYAPAP-VHCRPR-------   210
CD28-Protopterus-XP_043916657-NoLOC                                   ----GKVNGYGQADYMNMT--------PRRPINQK---YHPYAPQPRTYPKYC-------   211
CD28-Polypterus-XP_039613786-LOC120531949                             ----LTRSKQMQNDYMNMP--------SKQK-----KKQRVVHPNC--YHTRR-------   206
CD28-MsPaddlefish-XP_041120529-LOC121323502                           ----IQQNKLIQNEYMNMK--------PRGLK--NNKQQGVCHPSR--NGRY--------   212
CD28-Sterlet-XP_033899352-LOC117426134                                ----IRQNKLIQNDYMNMK--------PRGLK--NNKQQGVLHPSR--NGRY--------   241
CD28-Gar-XP_015214827-LOC106878821-LG12-27177048                      ----QKNKKHNQNDYMNMK--------PRGLK----KHQGVLHPTR--NGRY--------   220
Anguilla-CD28-XP_035264839-Chr3-si:dkey-1h24.6                        ----LKNKQNIQHDYMNMK--------PKAVR----KKQGVQHPVR--TGRY--------   204
CD28-1-Esox-XP_010898663-Chr20-42937353-LOC105027959                  ----LKKETSVQSVYMNVT--------PAERR----GQNRIQQPAI---GRF--------   231
CD28-2-Esox-XP_010898665-Chr20-42928519-LOC105027960                  ----LKKKKSENNIYMNVT--------PAEMK----GQKRVQKPAI---RR---------   229
CD28-Esox-XP_010891418-Chr22-17852626-si:dkey-1h24.6                  ----LRKGEGTQSDYMNTR--------PRAPPRVLKKNQGVQHPVR--MGRY--------   218
CD28-Onmy-XP_036831245-LOC118964336-Chr3-1-83647331                   ----LKKEVSAQTTYLNIR--------LGEKR----VPQRVQYPVT---GRF--------   231
CD28-Onmy-XP_021464608-LOC110527567-Chr7-1-25301800                   ----LRRVECSQSDDMNIK--------PKAPLRWPRKKQGVQHPIR--MGRY--------   226
CD28-Onmy-NP_001118004-LOC100136274-Chr7-2-25315200                   ----LRRVECSQSDYMDIK--------PKAPLRGHRKKQGVQHPIR--MGRY--------   229
CD28-Onmy-XP_021427178-si:dkey-1h24.6-Chr18-27925782                  ----LRRVKCSQSDYMNIK--------PKAPLWGPRKKQGVRHPIR--MGRY--------   234
CD28-Onmy-XP_036815388-LOC118943619-Chr22-1-50522004                  ----LKKEVSVQSDYVNMK--------PGEKT----RPKRVQHPVT---RRF--------   234
CD28-Icpu-XP_017313746-si:dkey-1h24.6-Chr26                           ----LKREDMSTHDYMNMK--------PRTRR----KKQGIHHPTH--HSWYNDTTNGTA   224
CTLA4-Hosa-NP_005205                                                  -----KRSPLTTGVYVKMP--------PTEPECE-KQFQP-YFIPIN-------------   223
CTLA4-Mumu-NP_033973                                                  -----KRSPLTTGVYVKMP--------PTEPECE-KQFQP-YFIPIN-------------   223
CTLA4-SwanGoose-XP_013027132                                          -----RRRRLTTGVYVKMP--------SEKLEKK---VIP-FHITVN-------------   205
CTLA4-Green_Anole-XP_003223584-NoLOC                                  -----KSMYFTPGIYEKII--------PM-------------------------------   193
CTLA4-Xenopustrop-XP_012825450-NoLOC-54,453,384                       -----QRKKFTVGNYEKML--------ESDQ--GNG--FSPYYIRVN-------------   209
Protopterus-CTLA4-XP_043916244-LOC122792727                           -----SRKNKEDTDYENMS--------TEYS-TRNT--TLPYQILND-------------   225
CTLA4-Polypterus-XP_039610963-LOC120530608                            ------------SEYVDTA--------PVIPEKMKT--NSRYVMF---------------   201
CTLA4-MsPaddlefish-XP_041119710-LOC121323053                          ------------ADYVDMS--------PVISEKKN--SNNRYVIL---------------   202
CTLA4-Sterlet-XP_033900514-LOC117426713                               ------------ADYVDMS--------PVISEKRN--SNNRYVIL---------------   202
CTLA4-Gar-XP_015214835-LOC102690085-LG12-27229939                     ------------SEYVDMA--------PVISRKV--DCQYGYENFL--------------   209
Anguilla-CTLA4-XP_035266074-Chr3-NoLOC                                ------------RMYPHMT--------PVTSNRV--DCRFGYENFL--------------   209
CTLA4-3-Esox-XP_012986927-Chr20-42914370-No                           ------------KRFGGIA--------PMISQ--N-DGRFGYGNFQ--------------   258
CTLA4-Onmy-XP_036831244-LOC110505466-Chr3-2-83647331                  ------------RRFGGIT--------PMISQ--N-DGRFGYGNFQ--------------   214
CTLA4-Onmy-NP_001118005-Chr22-2-50495067                              ------------RRFEAIV--------PMMSK--N-DGRFDYGNFQ--------------   217
CTLA4-Icpu-XP_017324683-NoLOC-Chr6                                    ------------SNHV-------------IQTHVS--QKGDYRNFW--------------   207
ICOS-Hosa                                                             --KYSSSVHDPNGEYMFMR--------AVNTAKKSRLTD---VTL---------------   199
ICOS-Mumu-XP_006496201                                                --KYGSSVHDPNSEYMFMA--------AVNTNKKSRLAG---VTS---------------   200
ICOS-SwanGoose-XP_013027130                                           NQQCESNSHEYNSEYMPMA--------AVNAAKKTRI-----------------------   212
ICOS-Green_Anole-XP_062818357-LOC134293749                            VCRHVKSSHDYNNEYMSMA--------AVKPS----------------------------   238
ICOS-Turtle-XP_026503133-LOC112110999                                 TQQCESNSHEFNSEYMPMA--------AVNAARKPAF-----------------------   213
ICOS-Alligator-XP_019347544-LOC102571456                              TKQCESNSHEYNSEYMPMA--------AVTAARKPRF-----------------------   213
ICOS-Xenopustrop-XP_031749766-LOC100493997-Chr9-51,952,126            LSQGNTQNNECNSEYMPMA--------SVNPAKRPVIPR---L-----------------   210
ICOS-Polypterus-XP_039613787-LOC120531950                             TRRRAAMKLDNNSEYMDMR--------KGLANGGGRR------PDH--WCAT--------   212
ICOS-MsPaddlefish-XP_041120674-LOC121323595                           --KRNTGEPESNSEYMDMR--------KVMSENTNRK------KER--DRNHNSEQFHF-   216
ICOS-Sterlet-XP_058889561-LOC117426591                                --KRNTVEPESNSEYMDMR--------KVMSENTNRN------RKR--DRNHNSEQFHF-   210
ICOS-Gar-XP_015214822-LOC107078819-LG12-27245174                      RQKRRRGDPDANSEYMDMR--------KVQARMQ--E------PSR--DRNFNSQHFHI-   238
CD28-CTL4-Cami-XP_007888795-NoLOC                                     ------EEQS--SEYVNM--------DP-AIQNS-RRQW---MNSA--YRT---------   228
CD28-CTLA4-Cami-XP_007888798-LOC103176829                             -EKGTTTELDHNSVYEKM-----------APTVNGDQSR---RNTK--TPNLVGSHHRLA   230
CD28-CTLA4-SCyCa-XP_038642918-LOC119958447                            ------QEEQS-NEYVNM--------EPPKLSSG-RRRG---NHIG--YRT---------   214
CD28-CTLA4-SCyCa-XP_038646294-LOC119962407                            ----------DDTIYINVR--------K--------------------------------   196
CD28-CTLA4-SCyCa-XP_038642929-LOC119958457                            ----LIKKEEENSVYERM-----------APSIGGEQSR---RNTE--SAATLS------   209
CD28-CTLA4-Leueri_XP_055494226_LOC129698880_Chr7_35060123-First       VITGISRDELRQDFYQEP-----------TPYSFKRENR------K--SRDTYQFKQQLQ   220
CD28-CTLA4-Leueri_XP_055494225_LOC129698879_Chr7_35020935-Second      ----------IDEVYINVR--------T--------------------------------   196
CD28-CTLA4-Leueri_XP_055494224_LOC129698878_Chr7_34987938-Third       ------QEEQN-NEYINM--------EPPKHASG-RKRR---MQSE--HRT---------   221
CD28-CTLA4-Hemoc_XP_060684085_LOC132817598_Chr7_112525810-First       ----VGKKEEENSVYEQM-----------APSNGGEHSR---RNTE--SATTLS------   214
CD28-CTLA4-Hemoc_XP_060684151_LOC132817669_Chr7_112615465-Second      ----------DDTIYINVR--------K--------------------------------   110
CD28-CTLA4-Hemoc_XP_060684086_LOC132817599_Chr7_112629451-Third       ------ACILRMKEAVDMKCELIHNYDEPATATW-TGQR---WETG--PRQEVAKEPRRT   232
CD28-CTLA4-Carcar_XP_041057727_LOC121285321_Chr12_125148145-Third     ------QEEQN-NEYVNM--------EPPKHSSG-RRRG---NHIG--YRT---------   212
CD28-CTLA4-Carcar_XP_041057149_LOC121285085_Chr12_125379474-First     ----LIKKEEENSVYERM-----------APSTGGEQSR---RNTE--SAATL-------   208


CD28-Hosa-AJ517504                                                    -------------------------------    234
CD28-Mumu-BC064058                                                    -------------------------------    218
CD28-SwanGoose-XP_013027129                                           -------------------------------    222
CD28-Green_Anole-XP_003223584-NoLOC                                   -------------------------------    232
CD28-Xenopustrop-XP_017952834-NoLOC                                   -------------------------------    210
CD28-Protopterus-XP_043916657-NoLOC                                   -------------------------------    211
CD28-Polypterus-XP_039613786-LOC120531949                             -------------------------------    206
CD28-MsPaddlefish-XP_041120529-LOC121323502                           -------------------------------    212
CD28-Sterlet-XP_033899352-LOC117426134                                -------------------------------    241
CD28-Gar-XP_015214827-LOC106878821-LG12-27177048                      -------------------------------    220
Anguilla-CD28-XP_035264839-Chr3-si:dkey-1h24.6                        -------------------------------    204
CD28-1-Esox-XP_010898663-Chr20-42937353-LOC105027959                  -------------------------------    231
CD28-2-Esox-XP_010898665-Chr20-42928519-LOC105027960                  -------------------------------    229
CD28-Esox-XP_010891418-Chr22-17852626-si:dkey-1h24.6                  -------------------------------    218
CD28-Onmy-XP_036831245-LOC118964336-Chr3-1-83647331                   -------------------------------    231
CD28-Onmy-XP_021464608-LOC110527567-Chr7-1-25301800                   -------------------------------    226
CD28-Onmy-NP_001118004-LOC100136274-Chr7-2-25315200                   -------------------------------    229
CD28-Onmy-XP_021427178-si:dkey-1h24.6-Chr18-27925782                  -------------------------------    234
CD28-Onmy-XP_036815388-LOC118943619-Chr22-1-50522004                  -------------------------------    234
CD28-Icpu-XP_017313746-si:dkey-1h24.6-Chr26                           VSKKLPT-------KGTSV------------    236
CTLA4-Hosa-NP_005205                                                  -------------------------------    223
CTLA4-Mumu-NP_033973                                                  -------------------------------    223
CTLA4-SwanGoose-XP_013027132                                          -------------------------------    205
CTLA4-Green_Anole-XP_003223584-NoLOC                                  -------------------------------    193
CTLA4-Xenopustrop-XP_012825450-NoLOC-54,453,384                       -------------------------------    209
Protopterus-CTLA4-XP_043916244-LOC122792727                           -------------------------------    225
CTLA4-Polypterus-XP_039610963-LOC120530608                            -------------------------------    201
CTLA4-MsPaddlefish-XP_041119710-LOC121323053                          -------------------------------    202
CTLA4-Sterlet-XP_033900514-LOC117426713                               -------------------------------    202
CTLA4-Gar-XP_015214835-LOC102690085-LG12-27229939                     -------------------------------    209
Anguilla-CTLA4-XP_035266074-Chr3-NoLOC                                -------------------------------    209
CTLA4-3-Esox-XP_012986927-Chr20-42914370-No                           -------------------------------    258
CTLA4-Onmy-XP_036831244-LOC110505466-Chr3-2-83647331                  -------------------------------    214
CTLA4-Onmy-NP_001118005-Chr22-2-50495067                              -------------------------------    217
CTLA4-Icpu-XP_017324683-NoLOC-Chr6                                    -------------------------------    207
ICOS-Hosa                                                             -------------------------------    199
ICOS-Mumu-XP_006496201                                                -------------------------------    200
ICOS-SwanGoose-XP_013027130                                           -------------------------------    212
ICOS-Green_Anole-XP_062818357-LOC134293749                            -------------------------------    238
ICOS-Turtle-XP_026503133-LOC112110999                                 -------------------------------    213
```

```
ICOS-Alligator-XP_019347544-LOC102571456                               -------------------------------    213
ICOS-Xenopustrop-XP_031749766-LOC100493997-Chr9-51,952,126             -------------------------------    210
ICOS-Polypterus-XP_039613787-LOC120531950                              -------------------------------    212
ICOS-MsPaddlefish-XP_041120674-LOC121323595                            -------------------------------    216
ICOS-Sterlet-XP_058889561-LOC117426591                                 -------------------------------    210
ICOS-Gar-XP_015214822-LOC107078819-LG12-27245174                       -------------------------------    238
CD28-CTL4-Cami-XP_007888795-NoLOC                                      -------------------------------    228
CD28-CTLA4-Cami-XP_007888798-LOC103176829                              -------------------------------    230
CD28-CTLA4-SCyCa-XP_038642918-LOC119958447                             -------------------------------    214
CD28-CTLA4-SCyCa-XP_038646294-LOC119962407                             -------------------------------    196
CD28-CTLA4-SCyCa-XP_038642929-LOC119958457                             -------CRY---------------------    212
CD28-CTLA4-Leueri_XP_055494226_LOC129698880_Chr7_35060123-First        IEKQNINAQYCLILSGANCTLLQFHAVYQLW    251
CD28-CTLA4-Leueri_XP_055494225_LOC129698879_Chr7_35020935-Second       -------------------------------    196
CD28-CTLA4-Leueri_XP_055494224_LOC129698878_Chr7_34987938-Third        -------------------------------    221
CD28-CTLA4-Hemoc_XP_060684085_LOC132817598_Chr7_112525810-First        -------RKY---------------------    217
CD28-CTLA4-Hemoc_XP_060684151_LOC132817669_Chr7_112615465-Second       -------------------------------    110
CD28-CTLA4-Hemoc_XP_060684086_LOC132817599_Chr7_112629451-Third        EQ-----------------------------    234
CD28-CTLA4-Carcar_XP_041057727_LOC121285321_Chr12_125148145-Third      -------------------------------    212
CD28-CTLA4-Carcar_XP_041057149_LOC121285085_Chr12_125379474-First      -------CKY---------------------    211
```

B - CD28H. Multiple alignment.

```
CD28H_Hosa-S1                                        ----------MGSP------------------G----------MV-LGLLVQIWA-----    16
CD28H_Gray-short-tail-opposum-XP_007488910-S1        ---------MLGEPLGIKESAINARDQKSGDMS----------SS-PSSASQSLL-----    35
CD28H_TasmanianDevil-XP_031803754-S1                 ------------------------------------------------------------    0
CD28H_Galgal-XP_015155314-S1                         -------MSREGA--------VGTAERTARGWG----------PHGAVGTASVWGC----    31
CD28H-threetoed-BoxTurtle-XP_029768145-S1            -------MQRLGER-----------------------------LL-LLLL-AAFLL----    18
CD28H_alligatormississippi-XP_019350811-S1           ---------MGGIL-----------------------------LL-LALL-PAFLP----    16
CD28H_Leueri_XP_055514805.1_LOC129711285_Chr29_20144833-S1 -----------------------------------------MDFFRRMFT-LHILL----    14
CD28H_Heoc_XP_060702444_LOC132829134_Chr28_35893212-S1 -----------------------------------------MNLFSKVLI-FQLLL----    14
CD28H_Cami-NoXp-LOC103183582-S1-NW_024704762-Partial -------------------------------------------------V-FRFLT-SAF    9
CD28H_Protopterus-XP_043946518-LOC122817548-S1       -----------------------------------------MGEANLSVI-IALLT----    14
CD28H_A-ruth-XP_058869996-LOC131721040-Chr47-S1      MLNYKRERERERERERERERENENENRTGPGWAGLAGLGGSMRRWNGVVF-LQLLT-CAV    58
CD28H_Gar-XP_015221026_LOC_107079672-LG19-9573216-S1 -----------------------------------------MVTGQGGLY-LLLLT----    14
CD28H_Anguilla-XP_035271816_LOC118226355-Chr4-7716843-S1 -----------------------------------------MDFFFFKIF-YLLLL-KAT    17
CD28H_Pike-XP_010870070-LOC105011605-Chr8-13751499-S1 -----------------------------------------MDIWKEVTL-LLLLL-ET-    16
CD28H_Salmon-XP_014072379-LOC106614013-S1            --------------------------MTCGDRLRSPPLSHKMDIWIKVTL-LLFLI-GTL    32
CD28H_Icpu-XP_017333266-LOC108270826-S1              -----------------------------------MLLTRTMDYLFNRNF-LLLLTVGAI    24
CD28H_Stickleback-XP_040041332-LOC120024500-S1       -------------------------------------------MCLERLT-LVLLS-VTL    15

CD28H_Hosa-S1                                        --LQEASSLSVQQGPN-------LLQVRQGSQATLVCQVD-QATAW------ERLRVKWT    60
CD28H_Gray-short-tail-opposum-XP_007488910-S1        --LARGTNYTVQQEYQ-------TLRVSEGGKVNMTCKVI-W-AKW------EQVRVEWK    78
CD28H_TasmanianDevil-XP_031803754-S1                 ----------MVQQAE-------QIKVDSGSKVNMDCQVT-W-THW------EQFRVEWK    35
CD28H_Galgal-XP_015155314-S1                         GGLGRSSPSSVQPQPCGWTSGRLSCGWVVGANASLPCQLV-PARPW------SVVRIAWL    84
CD28H-threetoed-BoxTurtle-XP_029768145-S1            AASKESETLQVRQDPA-------RILAPLGGSVELSCQIN-AAQHW------ERLRVEWR    64
CD28H_alligatormississippi-XP_019350811-S1           SEDNYTEALSVIQDPP-------QSQTLPGGSVTMRCHVFTRDQEW------SRLRLEWK    63
CD28H_Leueri_XP_055514805.1_LOC129711285_Chr29_20144833-S1 SAAANDVNLQVIQSPS-------YFNATEGDTVNMTCQIITWN---------ADLRIEWN    58
CD28H_Heoc_XP_060702444_LOC132829134_Chr28_35893212-S1 SVVGKSVILMVTQSPP-------YLSALEGGTATMTCWINSSS---------LERRIEWS    58
CD28H_Cami-NoXp-LOC103183582-S1-NW_024704762-Partial FTVQSNQFLEVVQTPE-------KLWQEPGGSVTMVCKFNSSS---------KDLRIEWN    53
CD28H_Protopterus-XP_043946518-LOC122817548-S1       LVGGLSYDQHVQQFPS-------HITAETGNAVTMNCIYNSTEGT-------QRVRMAFK    60
CD28H_A-ruth-XP_058869996-LOC131721040-Chr47-S1      RA--LWQGVSVSQSPS-------ALTVSWNGSVTLNCSFQTPAGA-------DRVRVEWI    102
CD28H_Gar-XP_015221026_LOC_107079672-LG19-9573216-S1 VSAGAQSILWVSQPPG-------PVMAAGGSSVSLNCSFGAESAA-------VQLRVEWR    60
CD28H_Anguilla-XP_035271816_LOC118226355-Chr4-7716843-S1 VEAVASRITFHPQTPP-------LVL--LRPFLTLNCSFDIEGDAYRVRVSAYRVRVNWF    68
CD28H_Pike-XP_010870070-LOC105011605-Chr8-13751499-S1 VKAA-LSTVILNQPPR-------EENVVLGSDLTLNCSFQN--L--------QRISVNWI    58
CD28H_Salmon-XP_014072379-LOC106614013-S1            VKPR-LSTVILDQPPR-------TVEVHLGSSLTLNCSFMP--Q--------TRVKVNWY    74
CD28H_Icpu-XP_017333266-LOC108270826-S1              INARVQGTRIEPEVPK-------MVKACTGSHTTISCMFHTDV---------KGLKVAWY    68
CD28H_Stickleback-XP_040041332-LOC120024500-S1       VISG-PVPVILDYEPR-------RVEVLLGSSVAFQCRLKTPSY--------GRVRVQWI    59

CD28H_Hosa-S1                                        KDGA--IL---CQPYITNGS---LS------LGVCGPQGRLSWQAPSHLTLQLDPVSLNH    106
CD28H_Gray-short-tail-opposum-XP_007488910-S1        KDEG--VL---CQSAPIKRE---SS------RVLCGSRDLLFWIPENIVILSLDRVTPND    124
CD28H_TasmanianDevil-XP_031803754-S1                 KDGK--KL---YQSLPISQN---SN------QVIWSSRNMSVLRRNNTITLSLNNVNVND    81
CD28H_Galgal-XP_015155314-S1                         KDGGSGAL---CTTRLRPEA---AA------VPCATPYLQLAWSPP-HANLSLRGAQEGH    131
CD28H-threetoed-BoxTurtle-XP_029768145-S1            RDSAPRAF---CQVVLDNAS---VSSCCRGMGACDDARLSFTWDPP-EFTLRMGNISEDD    117
CD28H_alligatormississippi-XP_019350811-S1           RRDLPTTL---CTALILLLS---------NLNHSCIPRFNLTWQWP-MVTLHLDNATEQD    110
CD28H_Leueri_XP_055514805.1_LOC129711285_Chr29_20144833-S1 KELPEGKTS------------LLTSKWNRTTYYLNYSERLQHFANETVSLLTISRVELND    106
CD28H_Heoc_XP_060702444_LOC132829134_Chr28_35893212-S1 KVLSEQRIL------------VLTSKGNITKPNLDYTKRMKHSLNDTVSTLSIYPIHRND    106
CD28H_Cami-NoXp-LOC103183582-S1-NW_024704762-Partial KHWPEGSLT------------VLTAKNKMSKAFQTYQNRTQHNFNSTFASLTISDVNPHD    101
CD28H_Protopterus-XP_043946518-LOC122817548-S1       RLAKQPNE-------------IIFSINNNTTIHHSYLKKVTYKKKDSFLEMTILNVEEDD    107
CD28H_A-ruth-XP_058869996-LOC131721040-Chr47-S1      RHSQQAGTDKDCPKQSKVSDTVLHGNKTETQSNQDTFQRVVQRTWYNGSALTLNQLTPQD    162
CD28H_Gar-XP_015221026_LOC_107079672-LG19-9573216-S1 KRDRPGSGGSGCQNGTKLISALASSNQ---TSARDGAGRLVQRVEANWSSLTLTGVTASD    117
CD28H_Anguilla-XP_035271816_LOC118226355-Chr4-7716843-S1 KKEGQSS---GCGEP-K-----FSSGLSNETASLVTRDRVTQWTGRNWSSLTLRDVTAND    119
CD28H_Pike-XP_010870070-LOC105011605-Chr8-13751499-S1 FHQNNKLS---CNDMTSAK----------------KLNDNSITTKDTWSALNLKQVTYND    99
CD28H_Salmon-XP_014072379-LOC106614013-S1            FSPTGHSS---CSSNTLLYS---STHSADKTVKLGAGGHESKESRKSWSRLILKDVTHNN    128
CD28H_Icpu-XP_017333266-LOC108270826-S1              FDKTSDFNDR--KNINE----LH-------SPPGSNVRHYHEEKGHTASYLTIRNVTLND    115
CD28H_Stickleback-XP_040041332-LOC120024500-S1       FSPSPSGN---TTILNI--------------SIYVLNQTQQTQETNRTWPTYTLANATKED    103

CD28H_Hosa-S1                                        SGAYVCWAAVEIPE-LEEAEGNITRLFVDPDDPTQ------------------------N    141
CD28H_Gray-short-tail-opposum-XP_007488910-S1        SGCYECHMKKEIPL-LEETNKT-TCLQVSGSGDFS------------------------K    158
CD28H_TasmanianDevil-XP_031803754-S1                 SGHYVCHVTMEIPE-LQTVEGNGTHLIVSGMGRRQEGRENEQCF-SLPISPLHDVSTGSP    139
CD28H_Galgal-XP_015155314-S1                         AGCYVCHVTVEVPY-LATAAGNGTALHVGAGADGAHGAGM--CAPTAPHGPPPPAAGGDE    188
CD28H-threetoed-BoxTurtle-XP_029768145-S1            VGWYVCKAIVEIPV-YLDATGNGTMLNTSDL-----------------------------    147
CD28H_alligatormississippi-XP_019350811-S1           VGVYECSVNVEIPN-LLSAVGNGTALNVSASAGRA-------------------------    144
CD28H_Leueri_XP_055514805.1_LOC129711285_Chr29_20144833-S1 TGQYICQASIEIPPPVFTKYGNGTYLQVQVGFKE-------------------------N    141
CD28H_Heoc_XP_060702444_LOC132829134_Chr28_35893212-S1 TGMYVCEVLIEIPPPVYRMSGNGTHLQVQVVNNE-------------------------M    141
CD28H_Cami-NoXp-LOC103183582-S1-NW_024704762-Partial SGLYVCEVFIEIPS-LHRDSGNGTSLQV--------------------------------    128
CD28H_Protopterus-XP_043946518-LOC122817548-S1       SDIYVCEVNFEIPL-LKQMKGGGTNLTIKDSFNYTAVNDDRISNPS--------------    152
CD28H_A-ruth-XP_058869996-LOC131721040-Chr47-S1      SGHYFCRVILEIPA-LQTSCGNGTLLYVEERPEQ-------------------------L    196
CD28H_Gar-XP_015221026_LOC_107079672-LG19-9573216-S1 SGHYVCVVVMEIPS-LAWHCSNGTQVDLEMSDGQSNSSDTAISV-----------INEDK    165
CD28H_Anguilla-XP_035271816_LOC118226355-Chr4-7716843-S1 SGWYFCEVVVEIPN-FQQKCNPGTEVIVPSSLRSSPATKTAAKHTT---------PRSPA    169
CD28H_Pike-XP_010870070-LOC105011605-Chr8-13751499-S1 SGWYYCKISVEIPI-LKQNCSNGTKVTVTAKRKD--------------------------    132
CD28H_Salmon-XP_014072379-LOC106614013-S1            SGWYFCHVSVEIPV-LQQACSNGTQVNISDNQMK--------------------------    161
CD28H_Icpu-XP_017333266-LOC108270826-S1              SGWYFCQVTQDIPI-LISNLSNGSELVISPPTECNVEDHTTNSS-T---------I---Y    161
CD28H_Stickleback-XP_040041332-LOC120024500-S1       GGCYHCKIIAEIPV-LDTTMSNTTQVVIRLPP----------------------------    134

CD28H_Hosa-S1                                        RNRIASFPGFLFVLLGVGSMG-VAAIVW-GAWFWGRR-SCQQRD--------SGNAFYSN    190
CD28H_Gray-short-tail-opposum-XP_007488910-S1        SDWINGYLGLLLWLLVACVMV-GSGAVL-GIKICR-F-LRRSRE--------SVHHFYGN    206
CD28H_TasmanianDevil-XP_031803754-S1                 SPLTISSQDFLIWLLMACVMI-GLGAVL-ARMIWR-C-FRRNTD--------SENHFYGN    187
CD28H_Galgal-XP_015155314-S1                         AVAHPAVLAELPWKLGGAAGG-SLLLVA-LISLCCWRRRR--RA--------DTPDVYVN    236
CD28H-threetoed-BoxTurtle-XP_029768145-S1            ---------VLWLGLAGALAV-TALLLL-PAVICCYKRRR--RD--------PGQAIYVN    186
CD28H_alligatormississippi-XP_019350811-S1           ----MSYIGLWWWLVIGGAAV-GIILLL-AVGLCCYRHRHLCRK--------TGEEIYEN    190
CD28H_Leueri_XP_055514805.1_LOC129711285_Chr29_20144833-S1 AISNGSRFGTATWILIGLLSPLALVITV-HVVFLTR--RFFCPR--------RGNPTYVN    190
CD28H_Heoc_XP_060702444_LOC132829134_Chr28_35893212-S1 AVSSNDNTTGSTWILIGCILSIILIVSV-TTSFLAR--KLI--K--------KEEPMYVN    188
CD28H_Cami-NoXp-LOC103183582-S1-NW_024704762-Partial ------------------------------------------------------------    128
```

```
CD28H_Protopterus-XP_043946518-LOC122817548-S1                 -HGKG-------NDFIGGITATAV---L-LIICLLL--AVYCFS-RSRKRQTKDSPVYVN   197
CD28H_A-ruth-XP_058869996-LOC131721040-Chr47-S1                EIPLS---HWVLWLLVGLGA---LGAVL-GAVWLCR--CLYGRW-RRSSPQLTENQIYEN   246
CD28H_Gar-XP_015221026_LOC_107079672-LG19-9573216-S1           NHGVTSPAGWRLWLLMSVGT---IAAVL-AVIAVCY--CLLRWKQKHRNTGTRDNPIYEN   219
CD28H_Anguilla-XP_035271816_LOC118226355-Chr4-7716843-S1       SLPHHVNAHWWVWVTVGVGSLMLAAIFIGGVRILCR--RMRCQN--------KENPIYEN   219
CD28H_Pike-XP_010870070-LOC105011605-Chr8-13751499-S1          ---PTYSPDWKVWVAVGVLGAILIIILVV-TFWMLFE--RKRRMR--------RENPIYTN   178
CD28H_Salmon-XP_014072379-LOC106614013-S1                      ------STTYTPLMTVGAASAIPVTLLV-LIWILLQ--RRRCKS--------RENPIYSN   204
CD28H_Icpu-XP_017333266-LOC108270826-S1                        ASEPPNASPWWLWVAVAGGCVLIITAAI-TTTVICR------RK--------KEAPVYEN   206
CD28H_Stickleback-XP_040041332-LOC120024500-S1                 ---PSQLMDGWMWISLGVSSVLLVVLLL-ICVSLSR--RRCRRR--------GEEPVYAN   180

CD28H_Hosa-S1                                                  VLYRPRG----APKKSEDCSGEGKDQRGQS-----------IYSTSFPQPAP--RQPHLA   233
CD28H_Gray-short-tail-opposum-XP_007488910-S1                  VLYYHQKSKEAKPGKNKTLACPVERERGES-----------IYSTSFPKPSP--RLAQAP   253
CD28H_TasmanianDevil-XP_031803754-S1                           VLYFHKETKGATPNKNKPLQGTPEKKRGER-----------IYSAGLQLPTP--TPRA--   232
CD28H_Galgal-XP_015155314-S1                                   IVSLRTPRNRS------PQGAAAGGQRIQR-----------GLDRAWEPPSP-------K   272
CD28H-threetoed-BoxTurtle-XP_029768145-S1                      VLFRKKQE----GKK--DGGQARSETKPGS-----------LYIQEFQRRGHNWKPPAAE   229
CD28H_alligatormississippi-XP_019350811-S1                     VEYCKQQE----ANK--STCCKGSQRNSPD-----------VYTVNLQSRGQLPVPPQYV   233
CD28H_Leueri_XP_055514805.1_LOC129711285_Chr29_20144833-S1     VEFRKK--GSKN--------SQLTEQ-RNARYVVS-------------------------   214
CD28H_Heoc_XP_060702444_LOC132829134_Chr28_35893212-S1         VRYRNK--TGEN--------KRQTESKCEVRYVV--------------------------   212
CD28H_Cami-NoXp-LOC103183582-S1-NW_024704762-Partial           ------------------------------------------------------------   128
CD28H_Protopterus-XP_043946518-LOC122817548-S1                 MISLKG--KQATN--------QSTCQEYEDHLYSR-------------------------   222
CD28H_A-ruth-XP_058869996-LOC131721040-Chr47-S1                MVRRVK--PSPP--------PPR-----SSTAVSTGA---------------S---RG--   271
CD28H_Gar-XP_015221026_LOC_107079672-LG19-9573216-S1           MTRSRP--VPPP--------STL-EQKPPELQCTPKP---------------Q---Q---   247
CD28H_Anguilla-XP_035271816_LOC118226355-Chr4-7716843-S1       MHPVHK--NAGHPQ---PSPRPRTQQNPSDPPSS--------------------------   248
CD28H_Pike-XP_010870070-LOC105011605-Chr8-13751499-S1          MPPPA--KKQPSPR---PGKQMDNQKISALEE-------------------LS---TAFD   211
CD28H_Salmon-XP_014072379-LOC106614013-S1                      MPPPRSAIKQPSPH---PGIQMDNQKIPSPLKHTRTP-PTAHYSEHLRTPTPA---RAND   257
CD28H_Icpu-XP_017333266-LOC108270826-S1                        TKEASKR--------------HWKEDKYLH-----HGMPSKGYS----------------   231
CD28H_Stickleback-XP_040041332-LOC120024500-S1                 TRPVT--SKQPSPR---PGSDHL--KEPSSLQNIRKPSPCRKYNEGKRRYK---------   224

CD28H_Hosa-S1                                                  SRPCPSPRPCPSPRPGHPV-SMVRVSPRPSPTQQPRPKGFPKVGEE-----   278
CD28H_Gray-short-tail-opposum-XP_007488910-S1                  VILPVHPRM--HPATSRPI-KTAYFPPTPHLS-------------------   282
CD28H_TasmanianDevil-XP_031803754-S1                           ---------------------------------------------------   232
CD28H_Galgal-XP_015155314-S1                                   G--------------------------------------------------   273
CD28H-threetoed-BoxTurtle-XP_029768145-S1                      RSPGPT------KASKKASSFTQRATERP----------------------   252
CD28H_alligatormississippi-XP_019350811-S1                     HHGK-A------PRAQHIHSSKQRAAKRP----------------------   255
CD28H_Leueri_XP_055514805.1_LOC129711285_Chr29_20144833-S1     ------------------GRCTDLKTPAPGLPG-TREGDLKRLNFDRPWMK   246
CD28H_Heoc_XP_060702444_LOC132829134_Chr28_35893212-S1         ---------------------------------------------------   212
CD28H_Cami-NoXp-LOC103183582-S1-NW_024704762-Partial           ---------------------------------------------------   128
CD28H_Protopterus-XP_043946518-LOC122817548-S1                 ---------------------------------------------------   222
CD28H_A-ruth-XP_058869996-LOC131721040-Chr47-S1                ---TVRPKP---PATRSTQSCEDASAPL--IRGADRSGPDPRSGPERP---   311
CD28H_Gar-XP_015221026_LOC_107079672-LG19-9573216-S1           -----RRLP---PS---SAAC----RPTPQSRSRSAVGV------------   271
CD28H_Anguilla-XP_035271816_LOC118226355-Chr4-7716843-S1       ----VHPGT---PK----------------IL-------------------   257
CD28H_Pike-XP_010870070-LOC105011605-Chr8-13751499-S1          NKLIRNPNP---PR---VHNCRQLRIPTPSQANGSKWSPKT----------   246
CD28H_Salmon-XP_014072379-LOC106614013-S1                      RQLFRTPNP---PR---VHEGKHLTIPTPATAHDNKFSPKP----------   292
CD28H_Icpu-XP_017333266-LOC108270826-S1                        HKQMDTLKP---HNHEYVHNG---RKPSPKL--------------------   256
CD28H_Stickleback-XP_040041332-LOC120024500-S1                 ---------------------------------------------------   224
```

C - CD28Hlike1. Multiple alignment.

```
Wombat-XP_027692242-LOC114023674-S3                      -----------------------------------------------------------M   1
Kiwi-XP_025910597-LOC112960496-S3                        ------------------------------------------------------------   0
SwanGoose-XP_013035024-LOC106035038-S3                   ------------------------------------------------------------   0
SoftShellTurtle-XP_025043104-LOC102462366-S3             ----------------------------------------------------------MM   2
ChineseAlligator-XP_025050903-NoLOC-S3                   -----------------------------------------------------------M   1
XenopusTropicalis-XP_031751177-LOC100489121-S3           -----------------------------------------------------------M   1
Leueri_XP_055499055.1_LOC129701708_Chr1_125270749-S3     -------------------------------------MQC----NRFLTAAEMNLNHIYL   19
Heoc_XP_060678526_LOC132833288_Chr36_26889991-S3         ----------------------------------------------------MNFNHDYL   8
Cami-NP_001279065-LOC103181322-S3                        ----------------------------------------------------MQLNPYYL   8
GrayBichir-XP_039607381-LOC120527712-S3                  ------------------------------------------------------------   0
Sterlet-XP_058863796-LOC131705367-S3                     MFCSWKTQDEVQQIRVEWCKVNQSRQELLHPLFMNGIKHCITDESAELTVGSVTQSDAGS   60
Gar-XP_015200293_LOC107077098-LG4-36807716-S3            ------------------------------------------------------------   0
Anguilla-XP_035282115_LOC118231881-Chr7-20402674-S3      ----------------------------------------------------------MS   2
Pike-XP_010886301-LOC105020751-Chr24-7296154-S3          ----------------------------------------------------------MK   2
RainbowTrout-XP_036814148-LOC110500750-S3                ----------------------------------------------------------MK   2
ChannelCatfish-XP_017328639-LOC108268284-S3              ----------------------------------------------------------MQ   2
Atlantic-Halibut-XP_034424619-LOC117751805-S3            ----------------------------------------------------------MK   2
Seabass-LOC127349514-XP_051231287-S3                     ----------------------------------------------------------MK   2
Seabass-LOC127349519-XP_051231292-S3                     ------------------------------------------------------------   0
Mosquitoefish-XP_043953957-LOC122820531-S3               ----------------------------------------------------MNRGGKMK   8
tilapia-XP_005463212-LOC102080180-S3                     ----------------------------------------------------MNQCGKMK   8
Tuna-XP_042256555-LOC121888973-S3                        ----------------------------------------------------------MK   2
Danio-XP_003200869-LOC795570-S3                          ----------------------------------------------------------MK   2
Danio-XP_021324888-LOC110438240-S3                       ----------------------------------------------------------MR   2
Stickleback-XP_040035616-LOC120821294-S3                 ----------------------------------------------------------MK   2
Stickleback-XP_040036613-LOC120821731-S3                 ----------------------------------------------------------MK   2

Wombat-XP_027692242-LOC114023674-S3                      ARVLELI-------------------------LL---VAPCGYLVLTSVNNLTITQSPSE   33
Kiwi-XP_025910597-LOC112960496-S3                        MIAIALQ-------------------------LI---LIMCFGSLATGPNNLAVIQTPTK   32
SwanGoose-XP_013035024-LOC106035038-S3                   MIIISLK-------------------------LL---LAMCFGSLATGPNNLTVIQTPTK   32
SoftShellTurtle-XP_025043104-LOC102462366-S3             TTIILML-------------------------IL---FTECFGCLANETNELSVRQTPSE   34
ChineseAlligator-XP_025050903-NoLOC-S3                   TMIILKV-------------------------FL---LTMFSDCLAMDTDTLKVSQTPTK   33
XenopusTropicalis-XP_031751177-LOC100489121-S3           ARSSDL--------------------------FI---LTLPLYWQVFGGAGLRVTQTPGT   32
Leueri_XP_055499055.1_LOC129701708_Chr1_125270749-S3     LLMKSLH-------------------------CI----QVLTQVNAVVTEGINVSQTPPE   50
Heoc_XP_060678526_LOC132833288_Chr36_26889991-S3         QFILTLQ-------------------------YT----HVLLQTTEAVLNRINVSQTPPE   39
Cami-NP_001279065-LOC103181322-S3                        MFILMLH-------------------------HI----HLLIPIAAETTFRNNISQSPAE   39
GrayBichir-XP_039607381-LOC120527712-S3                  --MLQLR-------------------------CL-VGSLLIFYLLTEYVMSIKVSQTPLH   32
Sterlet-XP_058863796-LOC131705367-S3                     YYCKVTVEIPSLRRGQSEAAVLSIRGNNRKSKGDKFNKTDKIFNKEGAKQNITISQFPLS   120
Gar-XP_015200293_LOC107077098-LG4-36807716-S3            --MSLFT-------------------------AL-LGCLLLPSLSSLDASKMNITNFPER   32
Anguilla-XP_035282115_LOC118231881-Chr7-20402674-S3      VLQNVLQ-----------------------SAFIFFGLFCKASC---NNEALTVTQSPPS   36
Pike-XP_010886301-LOC105020751-Chr24-7296154-S3          YLL---V-------------------------CLLLSSLWSISSWGITNTGLVVIQSPHY   34
RainbowTrout-XP_036814148-LOC110500750-S3                HLL---V-------------------------WLLLSSLCSLSSWGIRD-MLVVIQSPHN   33
ChannelCatfish-XP_017328639-LOC108268284-S3              HLCSTLY-------------------------GLLASCLC---CWGPSD-GFFVSQSPST   33
Atlantic-Halibut-XP_034424619-LOC117751805-S3            LLL---S-------------------------SLLLGSLCAISSRSASS-TDIYVTQTPD   33
Seabass-LOC127349514-XP_051231287-S3                     LLL---S-------------------------SLLLASLCALSSWSVSS-GTLVVTQTPA   33
Seabass-LOC127349519-XP_051231292-S3                     ---------------------------------------------MGVSSGTLVVTQTPA   15
Mosquitoefish-XP_043953957-LOC122820531-S3               LLL---R-------------------------SLLLSSLCALSSWSVSS-DALVVTQSPD   39
tilapia-XP_005463212-LOC102080180-S3                     ILL---S-------------------------SLLFASLCTLSSWSVSS-GTLVVIQIPG   39
Tuna-XP_042256555-LOC121888973-S3                        LLL---S-------------------------CLLLASLCDLSSWSVSSTGTLDVTQTAD   34
Danio-XP_003200869-LOC795570-S3                          FSSRLLF-------------------------GLLMPCLY---FRGS-S-EFCVYQSPSS   32
Danio-XP_021324888-LOC110438240-S3                       FYCTLIC-------------------------GFTLSCIC---FKGSYE-EVSVNRSLPN   33
Stickleback-XP_040035616-LOC120821294-S3                 LLL---S-------------------------SLLLASFCALSSWSVSSAGEPAVSQTPD   34
Stickleback-XP_040036613-LOC120821731-S3                 LLL---S-------------------------SLLLASFCALSSWSVSSAGEPAVSQTPD   34

Wombat-XP_027692242-LOC114023674-S3                      LQLLVGQSVEIICSWNVS-SEV---EQFRVEWKKVSHVQDSVTHPTKTLKDNATLIKKNL   89
Kiwi-XP_025910597-LOC112960496-S3                        VHLSIGDSTEIACIWEKI-SSI---ERYRVSWYLVN---EENI--TKTVT------SELV   77
SwanGoose-XP_013035024-LOC106035038-S3                   IHLSIGDSAEIACIWEK---SI---ARYRISWYLVT---KENI--TKTIS------SSLF   75
SoftShellTurtle-XP_025043104-LOC102462366-S3             IRVSTGMPAEINCSWKNN-DSV---KRFRVTWKIQNLTCKEGT--SKDLF------SKLY   82
```

```
ChineseAlligator-XP_025050903-NoLOC-S3                  IIAEAGQSVEVTCTWENH-DRV---ERYRVTWEKQDLSSPGTS--SEKLK------EEVH     81
XenopusTropicalis-XP_031751177-LOC100489121-S3          LSVTEGGNANITCSWDTE-YED---ERIRVWWKLYYESPRAAP--NGTVL------ANVM     80
Leueri_XP_055499055.1_LOC129701708_Chr1_125270749-S3    IEAFEGDTINMQCSCEKG--KI---DSIRVAWKKDSKEFLQATT--NT------------     91
Heoc_XP_060678526_LOC132833288_Chr36_26889991-S3        INACEGDSINMECSCEED-VQM---ESIRVDWTKDSKSILNATS--SNG-----------     82
Cami-NP_001279065-LOC103181322-S3                       IEAVEGDSVTMTCSYKRD-QKF---ESIRVEWTKESQLVLTGTS--SNL-----------     82
GrayBichir-XP_039607381-LOC120527712-S3                 ITVKVGDSVEMMCSWETS-QTL---PRIQVEWKKNNISHVISKK--TYL-N---------     76
Sterlet-XP_058863796-LOC131705367-S3                    ITAVEGESVEIHCSWEGV-EEA---GQMQVEWGKDERTILTSFKEQMIL-----------    165
Gar-XP_015200293_LOC107077098-LG4-36807716-S3           ITARVGDSVQMVCCWETQLQNL---EQLRLEWWKNNQSKLSSLN--YKKVK---------     78
Anguilla-XP_035282115_LOC118231881-Chr7-20402674-S3     ITVKEGEKVHMQCCWGGT-LNM---ERVRVIWNKTNTSSVIIN--TM-----------H     78
Pike-XP_010886301-LOC105020751-Chr24-7296154-S3         VTVTEGETVHIQCCWN---KNL---TRVTVNWKSNAAN----T--SELV----------N     72
RainbowTrout-XP_036814148-LOC110500750-S3               VTVTEGDSVQIQCCWN---QNV---SRGTVNWHKEGHIEIKYK--SVLV----------T     75
ChannelCatfish-XP_017328639-LOC108268284-S3             VTLWRGESVQITCSWN---ISI---SGVKVTWFKDNQRVE--------------------     67
Atlantic-Halibut-XP_034424619-LOC117751805-S3           VSVTEGDALNITCCFT---SKS---ERARINWRKHLTE-NGNQ--SSVI-----------     73
Seabass-LOC127349514-XP_051231287-S3                    VSAMQGETVNVSCCWTWT-GKP---ERMRVDWLKNQTS-VKKD--IFGL-----------     75
Seabass-LOC127349519-XP_051231292-S3                    VSAMEGETVNVSCCWTWT-GKP---ERMTVDWLKNQTS-VKID--IFSL-----------     57
Mosquitoefish-XP_043953957-LOC122820531-S3              VSVQEGETGNITCCWE---GTA---ERVGIKWLKNQTL-INNK--TVLI-----------     79
tilapia-XP_005463212-LOC102080180-S3                    VSFMEGETVNITCCWT---EIL---DRVRVTWLKNQSK-IKSE--SN-------------     77
Tuna-XP_042256555-LOC121888973-S3                       VSVVEGETVNITCCWT---GTF---GRVGVYWLKNQRE-IKNK--IRII-----------     74
Danio-XP_003200869-LOC795570-S3                         ITVNEGEYAQISCCWN---TST---YSVKVVWYINETKLS--------------------     66
Danio-XP_021324888-LOC110438240-S3                      ITVNEGESAQIICCWN---KRTEINKSLKVVWYKDEERIS--------------------     70
Stickleback-XP_040035616-LOC120821294-S3                VSVVEGETAEISCCWT---LEY---QKLRVTWLKNHTE-IKSK--M-IF-----------     73
Stickleback-XP_040036613-LOC120821731-S3                VSVVEGETAEISCCWT---LKY---QSVRVKWLKNHTE-IKSK--MMIF-----------     74

Wombat-XP_027692242-LOC114023674-S3                     YDR------------NVFIQDPGKEPLELKNLKVHDSGLYLCKITVDIPIYK-QAQGPGT    136
Kiwi-XP_025910597-LOC112960496-S3                       YIS------------N--VTHKNKDVLVIEAANVNNTGFYYCEIIIEIPFCE-RVYGNGT    122
SwanGoose-XP_013035024-LOC106035038-S3                  YLP------------N--VTHGNKDVLVIKAVNVNDTGFYYCEIIIEIPFCK-KLCGSGT    120
SoftShellTurtle-XP_025043104-LOC102462366-S3            YTV------------N--YTHKDQTVLRIDVVKGNDTGIYFCEITIEIPFMK-KGMGNGT    127
ChineseAlligator-XP_025050903-NoLOC-S3                  NTN------------T--TVQKNELVLTLKAVHVNNTGIYFCEINVEIPYLK-SGKGNGT    126
XenopusTropicalis-XP_031751177-LOC100489121-S3          WANNKSEVRVTSRDQSDYRLMKDKAELWLFSVNKSDAGTYICEVTIDKPELR-FGKGTGT    139
Leueri_XP_055499055.1_LOC129701708_Chr1_125270749-S3    ---------TANINRSSTSINETTARLTIKNIIQHDSGMYVCQVTVEIPPPIRKGSGNGT    142
Heoc_XP_060678526_LOC132833288_Chr36_26889991-S3        ------SVNQAYIDRLSYSVEKAKSKLTIKSITRDDAGLYRCNMVIEIPAPVRKGNGNGT    136
Cami-NP_001279065-LOC103181322-S3                       ------SL--KSTDRFSQVIRESGAILTIKNINLNDTGLYLCNVIVEIPILL-KVNGNGT    133
GrayBichir-XP_039607381-LOC120527712-S3                 ------TTVIEVVNRTTLKILNTSATLLISNTERSDVGKYYCILIYEIPILT-RESGNGT    129
Sterlet-XP_058863796-LOC131705367-S3                    -----------FRSQVNYCTKATSATMTIHKLTKQDAGTYYCRTR--------RGQGGGT    206
Gar-XP_015200293_LOC107077098-LG4-36807716-S3           ------GAQRCEENKYTYCVSRNCSEMTIPSVSENDTGSYSCKIIIEIPLLM-MAQGLGT    131
Anguilla-XP_035282115_LOC118231881-Chr7-20402674-S3     YKD---NATCERKDKYSSCFSGNCSKFNISSVSTNNTGVYLCKITIEIPIHK-EGNGSGT    134
Pike-XP_010886301-LOC105020751-Chr24-7296154-S3         KSQ-------CQKSASEQNVSC-CSNLTISNLNRNDSGTYTCKVSSEIPVLQ-QSVGNGT    123
RainbowTrout-XP_036814148-LOC110500750-S3               YSQ-------CYDRASQQTVACNCSNWTISNVTRNYTGTYICKVSLEIPSLI-ESVGDGT    127
ChannelCatfish-XP_017328639-LOC108268284-S3             --F-------NQTDKFTKTNNNNNTSSKLVLNDVNDAGFYICQVVQDIPRLV-TVNGTGT    117
Atlantic-Halibut-XP_034424619-LOC117751805-S3           --N-------KWPQGSQ--MNHSCSTLTFSNITREDSGKYICNVTVEIPLYS-MKEGNGT    121
Seabass-LOC127349514-XP_051231287-S3                    --T-------NQSQGSLRMNTSDCLNLTFTNISREDSGRYTCKASVEIPLLV-EFEGNGT    125
Seabass-LOC127349519-XP_051231292-S3                    --T-------NHSQGSLRMDTSDCLNLTFMKISREDSGRYTCKASVEIPLLV-EFEGNGT    107
Mosquitoefish-XP_043953957-LOC122820531-S3              -----------QSTGSKNEQKSKCSVLIIENMIRSDSGRYICKVTVEIPLLA-IAEGKGT    127
tilapia-XP_005463212-LOC102080180-S3                    --S-------NGSQVVLKEEGKNCASLNFSKITTEDSGTYICKVTVEIPSLT-EVKGNGT    127
Tuna-XP_042256555-LOC121888973-S3                       -ST-------NTSQGCQQKETCNCSTLTFTNITREDEGKYYCKVSVEIPFLT-VVKGNGT    125
Danio-XP_003200869-LOC795570-S3                         --D-------PK-QKLQEHQNLNCSILNLTNIQKNDTGHYVCEVTQDIPVLE-KKNGTGT    115
Danio-XP_021324888-LOC110438240-S3                      --D-------EKR-LNQTSLEENCSVLNIIYTIPNDTGDYVCKAIQDIPVLM-EYEGNRM    119
Stickleback-XP_040035616-LOC120821294-S3                --K-------NSSHGSLSDKTSYCSHLTLMNVARDDSGRYICEVTVEIPNLN-KAQGVGT    123
Stickleback-XP_040036613-LOC120821731-S3                --K-------NFSHGSLSDKTSNCSHLTLMNVARDDSGRYICEVTVEIPNLN-KAQGNGT    124

Wombat-XP_027692242-LOC114023674-S3                     LLTVKEKNTKK-------------------------------------------------    147
Kiwi-XP_025910597-LOC112960496-S3                       TLTVEMKEADS-------------------------------------------------    133
SwanGoose-XP_013035024-LOC106035038-S3                  TLIVEEKEAQS-------------------------------------------------    131
SoftShellTurtle-XP_025043104-LOC102462366-S3            TLIVEEKGLFAEAGS---------------------------------------------    142
ChineseAlligator-XP_025050903-NoLOC-S3                  TLIVAEKDST---SM---------------------------------------------    138
XenopusTropicalis-XP_031751177-LOC100489121-S3          ILYVTDSPSST--ES---------------------------------------------    152
Leueri_XP_055499055.1_LOC129701708_Chr1_125270749-S3    KLTVQANVNY--------------------------------------------------    152
Heoc_XP_060678526_LOC132833288_Chr36_26889991-S3        ILTVQADNDTPKV-----------------------------------------------    149
Cami-NP_001279065-LOC103181322-S3                       KLTVQERKEKN-W-----------------------------------------------    145
GrayBichir-XP_039607381-LOC120527712-S3                 QLDITEDVAKSNTAE---------------------------------------------    144
Sterlet-XP_058863796-LOC131705367-S3                    VLSVHGKTQKSEDVS---------------------------------------------    221
Gar-XP_015200293_LOC107077098-LG4-36807716-S3           TLIVNAKETDTQGSS---------------------------------------------    146
Anguilla-XP_035282115_LOC118231881-Chr7-20402674-S3     QLTVKRTRNPSTKEAGGSVKKAGDKVKEAEDKVKEDGGSVQETGDKVQEDGGSVQEAGGK    194
Pike-XP_010886301-LOC105020751-Chr24-7296154-S3         HLTVTSKNDGISGD----------------------------------------------    137
RainbowTrout-XP_036814148-LOC110500750-S3               RITVTSQNTTNNDS----------------------------------------------    141
ChannelCatfish-XP_017328639-LOC108268284-S3             NVTYNESGTSTTQCG------------TLTT--------QPIT-----------------    140
Atlantic-Halibut-XP_034424619-LOC117751805-S3           VITVKPRGSAENKDV-------------------------PPA-----------------    139
Seabass-LOC127349514-XP_051231287-S3                    VITVTAREARENTTD-------------------------AAE-----------------    143
Seabass-LOC127349519-XP_051231292-S3                    VITVTARENTKDHTN-------------------------PPT-----------------    125
Mosquitoefish-XP_043953957-LOC122820531-S3              TITVTSRKNITDSTT-------------------------KD------------------    144
tilapia-XP_005463212-LOC102080180-S3                    VITVREKT--ENDTG-------------------------NIS-----------------    143
Tuna-XP_042256555-LOC121888973-S3                       VITVKDRDNIKDKT---------------------------EE-----------------    141
Danio-XP_003200869-LOC795570-S3                         NVSISISKQNTTSNN------------DSDQ--------SPNT-----------------    138
Danio-XP_021324888-LOC110438240-S3                      MLYVNEIHTTTEEYVYT-VPSSTSRKPDVFH--------HPSL-----------------    153
Stickleback-XP_040035616-LOC120821294-S3                VLTVTAKDNTT-------------------------------------------------    134
Stickleback-XP_040036613-LOC120821731-S3                VLTVTAKDNAT-------------------------------------------------    135

Wombat-XP_027692242-LOC114023674-S3                     ----------Q----WP----WWTLVIFLLLT--F-STCLSYYC-KKRKNSQRI------    179
Kiwi-XP_025910597-LOC112960496-S3                       ---------LE----KRE---MAILAIIPFLV--A-VGSLYLCC-KKKQDSKLSGSTQLP    173
SwanGoose-XP_013035024-LOC106035038-S3                  ---------SE----KKV---LAVWSIIPLLV--A-VGSLYCCY-KKKRNSKFSGSAPLP    171
SoftShellTurtle-XP_025043104-LOC102462366-S3            --------TEK----YNF---MAILAIFPCLA--S-VACFYFCY-KKQQNSENS------    177
ChineseAlligator-XP_025050903-NoLOC-S3                  --------NKI----YYF---MTISVIIPILL--S-GGWLYVHC-KRQQDSQYS------    173
XenopusTropicalis-XP_031751177-LOC100489121-S3          --------TTD----FPK---VAYLAVFVIIA--A-VISLGYFL-YKRKKKANI------    187
Leueri_XP_055499055.1_LOC129701708_Chr1_125270749-S3    ----------------------SPKFCAVLLLLIL-VPAI-IYY-KQRQKRKTNARASG-    186
Heoc_XP_060678526_LOC132833288_Chr36_26889991-S3        ----------------------GALVGAVVLLLIL-VPVV-LFY-KKQQKIQRNARSTT-    183
Cami-NP_001279065-LOC103181322-S3                       ----------------------SLVGCVVLVVVIP-IIAV-LYY-KRRWKKHPETQVVS-    179
GrayBichir-XP_039607381-LOC120527712-S3                 --------SSY----I-----TTPTAVLLLLLLVL-IIVT-LVY-RRRRKPTST-RPEH-    182
Sterlet-XP_058863796-LOC131705367-S3                    --------DTAS--VYELTAIVYLLRSLPFLALLAAVFYL--TRGKKKTSAYQ--REVSS    267
Gar-XP_015200293_LOC107077098-LG4-36807716-S3           ----------------------AVLTVFPILLVLL-GVSL-LWY-WKRKKLTSYGPGLAP    181
Anguilla-XP_035282115_LOC118231881-Chr7-20402674-S3     VKEAGGSVDKAGDG-LEE-AVIFILRCLPFLTLLMAVFCL--CR--TETKAAA----PEN    244
Pike-XP_010886301-LOC105020751-Chr24-7296154-S3         --------LHNNLH-WSI-I-ALLVLLPPPLLLAL--LYL--YR-VKRKQGGASAKMVRV    181
RainbowTrout-XP_036814148-LOC110500750-S3               --------SEEKLP-QLL-PIMIPLAVLPLFLLPL--ICL--YRAWKRHQGGAKDRTVRV    187
ChannelCatfish-XP_017328639-LOC108268284-S3             ----V---LKNTPE-QP--VTLLVATLSSAVVILF--ICV--CLSVWRMKRGCKQSGRMV    186
Atlantic-Halibut-XP_034424619-LOC117751805-S3           ----------RSDE-VPI-IIA-AAVVGLLLLIAL--GCY--CTLRIRHGTTQ---AVRV    179
Seabass-LOC127349514-XP_051231287-S3                    ----DNSESSNSPP-LPL-IIS-VAAMVPLLLIAL--VCF--CS--LRRRQAQ---AARV    187
Seabass-LOC127349519-XP_051231292-S3                    ----ADNSDSNTNL-EDI-LIH-ALRCLPILALVT--SFI--CLKKLRTRAQQ---HTPA    171
Mosquitoefish-XP_043953957-LOC122820531-S3              --------QKENLP-QPV-IIA-MGVVVPLFLITL--ICF--CC--LRKKEGQ---AARV    184
tilapia-XP_005463212-LOC102080180-S3                    ----SGKSNFGRPP-ISV-IIS-LAVVVPLLLTTL--VCY--CT--LRRKQAL---AARV    187
Tuna-XP_042256555-LOC121888973-S3                       ----ESFSSSTGNP-LPL-IIT-LAVVAPILLLTL--VCV--CI------QAI---AARV    181
Danio-XP_003200869-LOC795570-S3                         ----K---TTGTQTWLL--DNFLPLSLGSAIGLLT--LCL--AFSICKM-RNCKKTERRV    184
Danio-XP_021324888-LOC110438240-S3                      ----ED--NKNQQEPQKKSSSGNIVMIYILRSLPF--ICL--LMAFFYLNRDYKRDTVSK    203
Stickleback-XP_040035616-LOC120821294-S3                ---------------------------------------------------GS-------    136
Stickleback-XP_040036613-LOC120821731-S3                ---------------------------------------------------VH---ATRV    141

Wombat-XP_027692242-LOC114023674-S3                     EPNRTDQEEKEDTEYIEMK-----RKMKENKRTPSNTTEWAISVLYEPMTSFAKKP----    230
Kiwi-XP_025910597-LOC112960496-S3                       MPEERHEEEQM-LQLDELHGRSEFINEAAEENSSCNSAEWAVSTLYESFDLFCNEP----    228
SwanGoose-XP_013035024-LOC106035038-S3                  VPEGGRQDEQT-LEAAELYGRNES---------TSEEAEWAVSTLYESLDSFYNES----    217
SoftShellTurtle-XP_025043104-LOC102462366-S3            VSERRTHEEQT-LQVMELDEGNEI-NEADERNSSSNSSEWVTSTLYESLDYFAMKQ----    231
ChineseAlligator-XP_025050903-NoLOC-S3                  VPVRRHQEEQM-LQVPELDEGNEERNEANDGNSSSNSSEWAISALYDSLDYFAMRR----    228
XenopusTropicalis-XP_031751177-LOC100489121-S3          LPLHQRAPE--AVELNEMQ---ENMN-DREESSSSDSVQWAISTLYESCNYFAMGT----    237
Leueri_XP_055499055.1_LOC129701708_Chr1_125270749-S3    ----RVVT--E--VINE-ENQENE--GSEENTHSRGSSHWMASSLYESFDYFTIKQ----    231
```

```
Heoc_XP_060678526_LOC132833288_Chr36_26889991-S3            ----AVPVLFD--VAKEEIIEQNQ--ENEESSSSRGSSHWLTSSLYESFDYFAIQN----      231
Cami-NP_001279065-LOC103181322-S3                           ----D---AVN--KINCEENRAEE--RNPESLSSRGSSQWMASTLYESIDYFAVKE----      224
GrayBichir-XP_039607381-LOC120527712-S3                     ----DQALDI---ELEEIESPYEE--VRDNSLSSRGSTQWATSMVYESFDYFAVKD----      229
Sterlet-XP_058863796-LOC131705367-S3                        RSFTEP------------------------------------------------------      273
Gar-XP_015200293_LOC107077098-LG4-36807716-S3               RIADAAPGESGALDQGEEDRHNEI--MEENSNSSPGSTHWALSAIYESFDYFNLHD----      235
Anguilla-XP_035282115_LOC118231881-Chr7-20402674-S3         KVQEGSVKEDE--EED------EA-LNESSTL-------LL-------------------      269
Pike-XP_010886301-LOC105020751-Chr24-7296154-S3             -IHQTPDYEDD--ELKTED-LRET-ADQTSDSSSRGSTQWCQVQVYESLDYLALP-----      231
RainbowTrout-XP_036814148-LOC110500750-S3                   -IHEAPNHEDE--ELKMEE-LEEA-ANQSSSSSSRGSTQWCQVQVYESLDYLALP-----      237
ChannelCatfish-XP_017328639-LOC108268284-S3                 -IREGPPSEGT--EPENS-------EDGGSSRTSRGSTQWYMVPVYESYFDLQRSD----      232
Atlantic-Halibut-XP_034424619-LOC117751805-S3               -IYEVPHIDSE--EAD---------MDKHSTSSSRGSSQWCQVPVYESFDY---------      218
Seabass-LOC127349514-XP_051231287-S3                        -IYEVPHTDSE--VAE---------MDKHSTTSSRGSSQWCQVPVYESFDY---------      226
Seabass-LOC127349519-XP_051231292-S3                        ALGNKPPSAQR---TE---------EDQEE--EDRDEREIVEV-----------------      200
Mosquitoefish-XP_043953957-LOC122820531-S3                  -IYEVPHTDSV--EAD---------MDKSSTSSSRGSTQWCEVAMYDSLDY---------      223
tilapia-XP_005463212-LOC102080180-S3                        -IYEVPHVDSD--VAE---------MDKHSTNSSRGSSQWCQVPLYESFYF---------      226
Tuna-XP_042256555-LOC121888973-S3                           -IYEVPHIDSE--VAD---------MDKHSTSSSSGSFQWCQVPVYESFDY---------      220
Danio-XP_003200869-LOC795570-S3                             -IHQTPHSEGE--EHEHME------EEEGSTGSSRGSLQWYQVPVYWSYFDLRRGE----      231
Danio-XP_021324888-LOC110438240-S3                          -PDEDAVESGE--GQN-QD------LE-------VGETQMNKT------EEVKQGD----      236
Stickleback-XP_040035616-LOC120821294-S3                    --------------------------------------HWEEVPVYVLRSLPLLALILAV      158
Stickleback-XP_040036613-LOC120821731-S3                    -IYEVPHIDSE--EVE---------MDKHSTSSSKGSSQWCQVPVYESFD----------      179

Wombat-XP_027692242-LOC114023674-S3                         ----------GEQRDDDVYTC---FC------SDPLQVLSP---GPSAIAPTTSTGAPET      268
Kiwi-XP_025910597-LOC112960496-S3                           ----------R-------------------------------------------------      229
SwanGoose-XP_013035024-LOC106035038-S3                      ----------K-------------------------------------------------      218
SoftShellTurtle-XP_025043104-LOC102462366-S3                ----------NEDKEEDNKHS-VAFS------SNAAD-----------------------      251
ChineseAlligator-XP_025050903-NoLOC-S3                      ----------DEDKRLPVTST-----------SDVADQ----------------------      245
XenopusTropicalis-XP_031751177-LOC100489121-S3              ----------PQNPEDYVYSL-AK------------------------------------      250
Leueri_XP_055499055.1_LOC129701708_Chr1_125270749-S3        ----------EEKEEACNISE-S-------------------------------------      243
Heoc_XP_060678526_LOC132833288_Chr36_26889991-S3            ----------INEETRCTVST-S-------------------------------------      243
Cami-NP_001279065-LOC103181322-S3                           ----------DENKESN-------------------------------------------      231
GrayBichir-XP_039607381-LOC120527712-S3                     ----------KEDAADMHKES---------------------------------------      240
Sterlet-XP_058863796-LOC131705367-S3                        ------------------------------------------------------------      273
Gar-XP_015200293_LOC107077098-LG4-36807716-S3               ------------------------------------------------------------      235
Anguilla-XP_035282115_LOC118231881-Chr7-20402674-S3         ------EAAAPADAEVAD-----------------------GSIGVSCSAP---------      291
Pike-XP_010886301-LOC105020751-Chr24-7296154-S3             ----------TKDNG---------------------------------------------      236
RainbowTrout-XP_036814148-LOC110500750-S3                   ----------TQDKG---------------------------------------------      242
ChannelCatfish-XP_017328639-LOC108268284-S3                 -----KEECADS-----DK---TACASALK------------------------------      249
Atlantic-Halibut-XP_034424619-LOC117751805-S3               -----FERVKNKESG---------------------------------------------      228
Seabass-LOC127349514-XP_051231287-S3                        -----FERVQTKE-----------------------------------------------      234
Seabass-LOC127349519-XP_051231292-S3                        ------------------------------------------------------------      200
Mosquitoefish-XP_043953957-LOC122820531-S3                  -----FERVEPKRTK---------------------------------------------      233
tilapia-XP_005463212-LOC102080180-S3                        ------EHVDPKESK---------------------------------------------      235
Tuna-XP_042256555-LOC121888973-S3                           -----LEHTESKQSSQTDLRQRDSIK--------PLNLIATA------VNS---------      252
Danio-XP_003200869-LOC795570-S3                             -----EQ-----------------------------------------------------      233
Danio-XP_021324888-LOC110438240-S3                          -----KVSVQDEESGNTEKAEATAVAEDEKQEKETFLVVDTEKLNESCLKT---------      282
Stickleback-XP_040035616-LOC120821294-S3                    LFITFLVRKAQQQTPAAPQNQHTPAR--------R-------------------------      185
Stickleback-XP_040036613-LOC120821731-S3                    ----YFERVETKESG---------------------------------------------      190

Wombat-XP_027692242-LOC114023674-S3                         PSEIPGKGRNLDLGYKQIEFSSEGTNLT      296
Kiwi-XP_025910597-LOC112960496-S3                           ----------------------------      229
SwanGoose-XP_013035024-LOC106035038-S3                      ----------------------------      218
SoftShellTurtle-XP_025043104-LOC102462366-S3                ----------------------------      251
ChineseAlligator-XP_025050903-NoLOC-S3                      ----------------------------      245
XenopusTropicalis-XP_031751177-LOC100489121-S3              ----------------------------      250
Leueri_XP_055499055.1_LOC129701708_Chr1_125270749-S3        ----------------------------      243
Heoc_XP_060678526_LOC132833288_Chr36_26889991-S3            ----------------------------      243
Cami-NP_001279065-LOC103181322-S3                           ----------------------------      231
GrayBichir-XP_039607381-LOC120527712-S3                     ----------------------------      240
Sterlet-XP_058863796-LOC131705367-S3                        ----------------------------      273
Gar-XP_015200293_LOC107077098-LG4-36807716-S3               ----------------------------      235
Anguilla-XP_035282115_LOC118231881-Chr7-20402674-S3         SSDVPNQD--------------------      299
Pike-XP_010886301-LOC105020751-Chr24-7296154-S3             ----------------------------      236
RainbowTrout-XP_036814148-LOC110500750-S3                   ----------------------------      242
ChannelCatfish-XP_017328639-LOC108268284-S3                 ----------------------------      249
Atlantic-Halibut-XP_034424619-LOC117751805-S3               ----------------------------      228
Seabass-LOC127349514-XP_051231287-S3                        ----------------------------      234
Seabass-LOC127349519-XP_051231292-S3                        ----------------------------      200
Mosquitoefish-XP_043953957-LOC122820531-S3                  ----------------------------      233
tilapia-XP_005463212-LOC102080180-S3                        ----------------------------      235
Tuna-XP_042256555-LOC121888973-S3                           SSDVRGA---------------------      259
Danio-XP_003200869-LOC795570-S3                             ----------------------------      233
Danio-XP_021324888-LOC110438240-S3                          EEDTRLMSDVEEERVTVL----------      300
Stickleback-XP_040035616-LOC120821294-S3                    ----------------------------      185
Stickleback-XP_040036613-LOC120821731-S3                    ----------------------------      190
```

D - CD28Hlike2. Multiple alignment.

```
Protopterus-LOC122794129-XP_043918243-S4                    --------------MYFRAHLFCLLLVYIGGHTQLNIIQYPPEVDEFLQNSVILSCQWET      46
Bichir-XP_039607697-LOC120527860-S4                         -----------------------------------------------------MICSWNV      7
MississippiPaddlefish-XP_041124223-LOC121325573-Chr1-S4     ---------M-ACLQTIKNLLFFHLIC--IEGCDINVTQSPSEISIDRGSSALLTCQWNS      48
Gar-XP_015200297_LOC107077099-LG4-44951368-S4               ---MRNSQFD-KPLFWIISHLFLFHIN--ASGYSLIVTQNPTEVDIHLKTSVSLSCQWTT      54
Bonefish_albula_goreensis_KAI1883834                        ---MSTQIFVVFMPFWIITSVV-TVTV--VGGDTCNISQSPAELTVEVGANIQLLCTWIS      54
EuropeanConger-XP_061106870-LOC133134649-S4-partial         ------------------------------------------------------------      0
greatwhiteshark-XP_041039503-LOC121275849-S4-Tooshort       MKIRSTSLYK-RLLLIIQQILLLPHLASAGETGDLKITQLPQAINISEGESITMTCLWNI      59
Heoc_XP_060678526_LOC132805451_Chr1_35475713-S4             MDVTSASLCK-RLLFIIQHILLLPLLTTADGVENLTIRQIPKAINISEGDTATLKCSWTI      59

Protopterus-LOC122794129-XP_043918243-S4                    KPTFERISIEWFKCEDTKTTLTEIKGGEKMNYTLFTKDKTRRLNESILNIGKALLNHRGI      106
Bichir-XP_039607697-LOC120527860-S4                         TQDIRRLSVTWNNINNSWSFLHMYNQTNKHKTEG-NKEGTISGTNAIIDIINTTMNDSGL      66
MississippiPaddlefish-XP_041124223-LOC121325573-Chr1-S4     SCEVQRIRISWFKDRKKINSTIQTT--------K-N-ISSLHRGKTYFKIHNTGVNETGV      98
Gar-XP_015200297_LOC107077099-LG4-44951368-S4               NSSAQKVRVIWYKTDQPANELQTVV--------L-N----GTERTNTFSLKISRSNDSGV      101
Bonefish_albula_goreensis_KAI1883834                        DYRVGTIRILWSKDGERKLSTRENA------------SKPVMNGTFSFNITSLKANDSGL      102
EuropeanConger-XP_061106870-LOC133134649-S4-partial         ----------------------------------------------------MKANDSGV      8
greatwhiteshark-XP_041039503-LOC121275849-S4-Tooshort       SH-SESVRVDWLKDNINIVTKTN-----KGIYKSDGRKYIIERNYSNLTIPNTVLNDSGL      113
Heoc_XP_060678526_LOC132805451_Chr1_35475713-S4             NQ-SESVRVDWLQNSDTIFTKPA-----KGQYKFDRREYIIEENFSMLIISKAVLNDSGL      113

Protopterus-LOC122794129-XP_043918243-S4                    YYCVVTVEIPPPPKPHFGNGTRLIIKERERKV-EI-KWIM--ITAGGISLLIAVTGLGCC      162
Bichir-XP_039607697-LOC120527860-S4                         YQCEVRIEIPAPLLKGKSNLIELLVQKRFQTNSDLSVVTVSIITISAGLVVVFIV---VA      123
MississippiPaddlefish-XP_041124223-LOC121325573-Chr1-S4     YWCNVIVEIPLPMQNGTGPGTTLKFNANKTNEDWKSAWTVPIAAALAGAAI-LVILFTG-      156
Gar-XP_015200297_LOC107077099-LG4-44951368-S4               YICKVIVEIPLFLQQQGN-GTQVFVYGYNSTMDAGLSWKVLTAACAGGAAV-LLILAAG-      158
Bonefish_albula_goreensis_KAI1883834                        YGCEVTVEIPTLASCYGT-GTNLTVKDSPKETKPF-PQYLLIVAICGGSLILLTITLSAS      160
EuropeanConger-XP_061106870-LOC133134649-S4-partial         YVCKIKVFIPVLEQCDGN-GTILSVDVLVAKTTLI-PQSVLIAAVCGATLIIFLVALCGS      66
greatwhiteshark-XP_041039503-LOC121275849-S4-Tooshort       YHCEVLVEIPPPVHRAIGEGTLLIVFAAQAKNNLI-IWLL--AAI-SPTILIAIVVLSCS      169
Heoc_XP_060678526_LOC132805451_Chr1_35475713-S4             YHCKVFVEIPAPIRRASGEGTLLIVFASQVKNYLI-IWLL--ASI-SPIMLIVIVVACCC      169
```

```
Protopterus-LOC122794129-XP_043918243-S4                LRNNAKRVNTDGTSQLRKSFRFHIEDQDAPESKCKMLTKAATSPATEDLSDNNEYKRELK    222
Bichir-XP_039607697-LOC120527860-S4                     VCRWLRNYT----SFKGENP-IYENQRTVKIKKQR----------------------MKG    156
MississippiPaddlefish-XP_041124223-LOC121325573-Chr1-S4 -YRCLRRTCWHGG---SDIP-VYVNNKVPPKS--------------------------LK    185
Gar-XP_015200297_LOC107077099-LG4-44951368-S4           -CLALRRTLKQEKKEEPSVP-IYMNSTQGFQKKQMS-S-------------DNKWKKMNE    202
Bonefish_albula_goreensis_KAI1883834                    LCKFFRRRQWEDHTYSNTSG-VGRKQNLRGADGQSEEK-------------EDQNKNIND    206
EuropeanConger-XP_061106870-LOC133134649-S4-partial     LWKSVRHRRLDEHTHTNTSE-GYMDKR-KRPDNQT--E-------------DDEKKDIKE    109
greatwhiteshark-XP_041039503-LOC121275849-S4-Tooshort   VRKIKNRRQNANSVYENSSV-IKKEA--F---KPKIEKR---------------------    202
Heoc_XP_060678526_LOC132805451_Chr1_35475713-S4         FRKVKNRRQNGNLSNLRRSSQLYVNNIELSQACQKMEAKSA-----------NIYKNALN    218

Protopterus-LOC122794129-XP_043918243-S4                SENGNKS-------------------SKQNAKRKVMKHQCQEKKERTANNPASKAMSCNN    263
Bichir-XP_039607697-LOC120527860-S4                     SHTKEHKEIS---------------R-QKVAARR--------------------------    174
MississippiPaddlefish-XP_041124223-LOC121325573-Chr1-S4 DHSTKTAEEIKTASDFSSNKDFQVNPTELVETRNKMKKQKAHFFHGN-------------    232
Gar-XP_015200297_LOC107077099-LG4-44951368-S4           DHGPENTTVSKTLPDSYSNQFIRVDCEDIFAVQRKMKNKRANFFMGN-------------    249
Bonefish_albula_goreensis_KAI1883834                    EKNETEVKAN-DKSLADDGRPFQVNREDIVEARKNLKTKRAYFFRRG-------------    252
EuropeanConger-XP_061106870-LOC133134649-S4-partial     VKDKVNITES-ETCADDLGTLFQVSKEDIVEARKKLKVKRAGFFTGG-------------    155
greatwhiteshark-XP_041039503-LOC121275849-S4-Tooshort   ------------------------------------------------------------    202
Heoc_XP_060678526_LOC132805451_Chr1_35475713-S4         VQQKKHQ-----------------------SANKVIEEKSSN------------EL----    239

Protopterus-LOC122794129-XP_043918243-S4                DNKRELKSEKRIMSSKQNGNMKVMKHQCQAKKERAVHKNPATPSCKPPGSAPVKSSSNPR    323
Bichir-XP_039607697-LOC120527860-S4                     ----NPKS------------------DSLPSPDSSSHIYINSRDLRKHPFPGNQ------    206
MississippiPaddlefish-XP_041124223-LOC121325573-Chr1-S4 -AQLKRKT------------------EGQFKKKRSLHIYINSRELKKHCKQTTDASDN--    271
Gar-XP_015200297_LOC107077099-LG4-44951368-S4           -ANSSLNT------------------EGKFQRKRSLHIYINSRDLRKQTQQPVGDNLA--    288
Bonefish_albula_goreensis_KAI1883834                    -EKSNANT------------------DGHFKRKRSLHLYINSRDLKKQAQIKKAPGGDPA    293
EuropeanConger-XP_061106870-LOC133134649-S4-partial     -GKPDTSG------------------NAHLKR-KSFHLYINSRELKKQSERTPHVG--PT    193
greatwhiteshark-XP_041039503-LOC121275849-S4-Tooshort   ------------------------------------------------------------    202
Heoc_XP_060678526_LOC132805451_Chr1_35475713-S4         --VNNKTQ---------------------------------------------------     246

Protopterus-LOC122794129-XP_043918243-S4                SGQLRPSSTPCSGHQKHSNKRNLPKKRQTPVLQDDSDAIYQNL----    366
Bichir-XP_039607697-LOC120527860-S4                     ----TKV---VTHK---KK-VSLP-------HEDRSKDIIEDIAPNV    235
MississippiPaddlefish-XP_041124223-LOC121325573-Chr1-S4 ----NKY---KA-Q---RSHKALQKQNRMK---GMPEDVFQTVVC--    302
Gar-XP_015200297_LOC107077099-LG4-44951368-S4           ----HKI---CDPV---KNH-----KNKMVKSGDNCEDV--------    312
Bonefish_albula_goreensis_KAI1883834                    QGKTKHT---CVHQ---KRQQ------RARVGQPRAKDLYENI----    324
EuropeanConger-XP_061106870-LOC133134649-S4-partial     QA--KYN---CTPQ---KKR---------------------------    205
greatwhiteshark-XP_041039503-LOC121275849-S4-Tooshort   -----------------------------------------------    202
Heoc_XP_060678526_LOC132805451_Chr1_35475713-S4         -----------------------------------------------    246
```

E - CD28Hlike3. Multiple alignment.

```
Bichir-XP_039627580-LOC120540683-SNew                   MH------CFIRW-LCLSTVFLSGQM------NFTVYQHPESITVSAGSNITLRCFFTAP   47
Gar-XP_015221322-LOC107079700-Snew                      MA------ALLRLLVIVQAARASGQR------LLGVRQWPESLRAEEGGEAAISCSFNGT   48
Sterlet-XP_058888659-LOC117427588-Chr11-SNew            MH------SFLRIVLCACLISPSGHA------FTPVHQTPRSVTVQAGGSVTLTCSFNGT   48
Sterlet-XP_058850216-LOC117973280-Chr21-SNew            MH------SFLRIVLCACLISPSGHA------LTPVHQTPRSVTVQAGGSVTLTCSFNGT   48
Xyrauchen-Texanus-XP_051965377-LOC127631324SNew         MIHTIC--PGALIIICLTG-LVPGFQL-------SLQQTPERINTTEGSNVSITCHIEVD   50
Cyprinus-carpio-XP_018965266-si:dkey-63d15.12-ChrA8-SNew MTRISL--SALFVTIWFTG-LVPGDVSLPNLGILSVWQIP-EITAKEGTNVIITCHIKAG   56
Sinocyclocheius-XP_016361154-NoLOC-SNew                 MTRTSL--STLFVTIWFTG-LVPGDVSLPNLGI-SVWQIP-EITAKEGTDVIITCHIKAD   55
Danio-XP_017213004-si:dkey-63d15.12-SNew                MTRSRL--SALILTICFTG-LFSRDAFSFHLP-ISVWQTP-NITAKEGTNVSITCHINAD   55
Icpu-XP_053536004-LOC108264924-SNew                     MTHTRRAAHLLFLIVFLTG-CVCGLNL-------SVWQTPRNITAESGERVTLTCHFTLR   52
Pangasius-XP_034162676-LOC113546007-SNew                MTHTRRIACLIFLIVFLEG-CVCGLNP-------SVWQTPRIITAESGERVNISCHFTLT   52
Silurusm-XP_046727643-LOC124400094-SNew                 MTHTRRAALLFFLLVFFTG-YVCGFNL-------SVWQTPRNITAVCGEHVNITCHFTLK   52
RedPiranha-XP_017539400-si:dkey-63d15.12-SNew           MTGIRAL-RTLLLSLFVPG-CVCGSDL-------TVWQTPEIINTTEGQHVNISCHFTVK   51
MexicanTetra-XP_049342048-si:dkey-63d15.12-SNew         MAHIHSV-YTLLLSAFLTG-CVCESDL-------TVWQNPERINAIEGQPVNINCSFKFN   51

Bichir-XP_039627580-LOC120540683-SNew                   E----FANLTQVRWYKILNQSENFQVIEE--SKLKSVLANRFYFSNESSNSLQLYLALVG   101
Gar-XP_015221322-LOC107079700-Snew                      G----LE-TVSVEWYRNPTASEAPPEKRYT----------RQNV-TGQGTVAVLTLRALR   92
Sterlet-XP_058888659-LOC117427588-Chr11-SNew            G----LE-IIKVRWFRYSSDNTLLQIDISLQHNVCSEFINRAAVKENQSIS-ILSLNALL   102
Sterlet-XP_058850216-LOC117973280-Chr21-SNew            G----LE-MVKVRWFRYSADNTSLQIDISLQHNVCSEFINRAAVKENQSIS-ILSFNALL   102
Xyrauchen-Texanus-XP_051965377-LOC127631324SNew         S----RTERLHVLWLKNNVSIM-DYEI-FN----VSKNQFSTCSTGYMCINASIHIPSVY   100
Cyprinus-carpio-XP_018965266-si:dkey-63d15.12-ChrA8-SNew S----HVKRIMVKWYRDNQTIP-F--T-TT-------SHHRSPVNGSVRINATLHLLSVH   101
Sinocyclocheius-XP_016361154-NoLOC-SNew                 S----HVERIMVKWCQDNQTVP-N--S-TT-------SYHRLPVNGSGRISATLQLLSVN   100
Danio-XP_017213004-si:dkey-63d15.12-SNew                S----RVDRILVKWWQDNQTEV-RSEG-IT-------SPNRLSVNGFVFINSTLDLLSVH   102
Icpu-XP_053536004-LOC108264924-SNew                     T---SEGGRWKLQWRKNNSTVA-PNES-YN----ISVINTNT--VTEVNKSHTLTLHPVS   101
Pangasius-XP_034162676-LOC113546007-SNew                NA--SQQGRWKVAWRKNNDTVF-LNGI---------NISDFT--LTDVNKSHTLTLSSVN   98
Silurusm-XP_046727643-LOC124400094-SNew                 MISTSYTEEWKVYWKKDNINET-SNKL-YN----ISDFNSDT--GSDVNKSHTLKLHAVS   104
RedPiranha-XP_017539400-si:dkey-63d15.12-SNew           S----TWEGLKVEWWRNNKTEV-KQEL-FN----ISALNSIN--PESMNHSSVLQIPSVR   99
MexicanTetra-XP_049342048-si:dkey-63d15.12-SNew         S----TTELLKVKWLKNNTEVD-KHEP-YN----ISALRSQNFNSRTVSQTSVLHLPSVQ   101

Bichir-XP_039627580-LOC120540683-SNew                   SADSGTYICEVTRWIPSPLIRRSGNGTAVTVTDEDP------------------------   137
Gar-XP_015221322-LOC107079700-Snew                      PNDSGVYACKITPLIPVQLEGAWGNGTRLTVSGASG------------------------   128
Sterlet-XP_058888659-LOC117427588-Chr11-SNew            PNDSGVYVCEVQRWAPAPYAWEQGNGTLVNVTREDP------------------------   138
Sterlet-XP_058850216-LOC117973280-Chr21-SNew            PNDSGVYVCEVQRWVPGPYAWEQGNGTLVNVTREDP------------------------   138
Xyrauchen-Texanus-XP_051965377-LOC127631324SNew         LNHSGVYYCKVWSDLPKLFE-AYGEGSHVYVGTSQISNITFSTLVT-----------SPG   148
Cyprinus-carpio-XP_018965266-si:dkey-63d15.12-ChrA8-SNew LNHSGIYYCTAQVDLLMLGPVEHGNGTHVYVVPNST-VT---------------------   139
Sinocyclocheius-XP_016361154-NoLOC-SNew                 LNHSGIYYCTAFVDLPTLEPAELGNGTHVYVVPNSTTVT---------------------   139
Danio-XP_017213004-si:dkey-63d15.12-SNew                SNHSFVYYCSAQLDLPELGPVEYGKGTHVFVESAQVTVED--------------------   142
Icpu-XP_053536004-LOC108264924-SNew                     VSDSGVYYCKVWQDVPRLRSETVGGGTELTVYNKTNNSSSTPSTLSTSTPSTLSTSTPSS   161
Pangasius-XP_034162676-LOC113546007-SNew                ESHSGIYYCTVWQDVPHLMSGADSTGTQLIVNSIS----------------------PDT   136
Silurusm-XP_046727643-LOC124400094-SNew                 VNHSGVYYCTVLKHIPKLVQFTDTTGTRLIVEPLVRN-----------------------   141
RedPiranha-XP_017539400-si:dkey-63d15.12-SNew           LNHSGIYYCAVFQELPMLGEKVYGPGTELRVD----------V-----------------   132
MexicanTetra-XP_049342048-si:dkey-63d15.12-SNew         LNDSGTYYCKVWYDVPLLGVKISEQGTELRVVPVNFTIVTSPA-----------------   144

Bichir-XP_039627580-LOC120540683-SNew                   --VDIGSRSFLTEVLLYGALSSVPLI-----VVALILR-----CKRAKKRGKNLNPNSDL   185
Gar-XP_015221322-LOC107079700-Snew                      --S------RLVAALPWALVPALVLL-----AAAAALC-----CSRARRDTGNNI-YTEA   169
Sterlet-XP_058888659-LOC117427588-Chr11-SNew            --ALISTDTRPADIIYYIAGVLIIIIALGLGIVALVYF-----NLRVNKSSVSRVPGIQA   191
Sterlet-XP_058850216-LOC117973280-Chr21-SNew            -------DTRPADIIYYIAGVLI-IIALGLGIVALVYF-----KLRVNKSSVSRVPGIQA   185
Xyrauchen-Texanus-XP_051965377-LOC127631324SNew         PPSTMGPANHYKYIVMWLPLSGCAILLLVIGIACVIYIYNKGHCRSQVKAKESNV---DA   205
Cyprinus-carpio-XP_018965266-si:dkey-63d15.12-ChrA8-SNew ------TQTLTMNTSMWSVLSGLGFVLLIICIVCVIHIHYKGHCRIEPRAKESLV---DV   190
Sinocyclocheius-XP_016361154-NoLOC-SNew                 ------TQTLTMNTVMWSVLSGLGFVLLIICIVCVIHIHYKGHCRIESRAKEALV---DV   190
Danio-XP_017213004-si:dkey-63d15.12-SNew                ------SGNLHSNTVMWSLFSGLGLVVLIICIACGIHISFKGHSKMEPGTKHGIV---AA   193
Icpu-XP_053536004-LOC108264924-SNew                     TPSSTPATHQDVKLVIWM--ASLALLLIIACLTSAMCFIYRGRCVRPKLKTDR-------   212
Pangasius-XP_034162676-LOC113546007-SNew                TADQSVTSVTSVMLVMWA--APLALALIIGCFTSVTCCFYRARCMRRKK--EN-------   185
Silurusm-XP_046727643-LOC124400094-SNew                 ------ATGRSVESVMWT--APLALALALFCVVSVVCCKNRGLCRRGKKEG---------   184
RedPiranha-XP_017539400-si:dkey-63d15.12-SNew           ------PTDHSAVTVLGA--SVLTSVLITVFITCVVFLIYRGCCTRPKQQS---------   175
MexicanTetra-XP_049342048-si:dkey-63d15.12-SNew         ------PPKPFAVTIMGV--SALGLVLMTACITAALCITYKRRRKRPKDEGED-------   189

Bichir-XP_039627580-LOC120540683-SNew                   QEENSI----LNKTEEPSVINVCYAEVAFLKKTRREENTSTHNKIVP------GGLQPE-   234
Gar-XP_015221322-LOC107079700-Snew                      DRGPPQARASREPEPEPELEDFGYSVLRFPAGPSSRGTC------A----AARGGGEPAA   219
Sterlet-XP_058888659-LOC117427588-Chr11-SNew            H-STPE----DTVCSRPDTHEVCYASLNFNKTRRTTDPSTT-GLMHSQYEATESTLQAKA   245
Sterlet-XP_058850216-LOC117973280-Chr21-SNew            H-STPE----DTVCSRPDTHEVCYASLNFNKTRRTTDPSTT-GLMHSQYEATESTLQAKA   239
Xyrauchen-Texanus-XP_051965377-LOC127631324SNew         F--------SGSLCPVP-SETIVYAALNIPKSADSAKTRNEDC-------------L--A   241
Cyprinus-carpio-XP_018965266-si:dkey-63d15.12-ChrA8-SNew T--------RGSPCPDAHQTHVVYAALNIPQ--DSVKSRKGNC-------------P--A   225
Sinocyclocheius-XP_016361154-NoLOC-SNew                 T--------RGSPCPDAHQTHVVYAALNIPR--DSVKSRKENC-------------P--A   225
Danio-XP_017213004-si:dkey-63d15.12-SNew                N--------SESPGSDT-TERVIYTALNIPQ--DSVKSRNENC-------------A--A   227
Icpu-XP_053536004-LOC108264924-SNew                     -----------QSAQAE-DLGVVYAAVKIHKPREKKQTQEASE-------------AQ--   245
Pangasius-XP_034162676-LOC113546007-SNew                -----------QPAQKD-DEGVVYAAVNIHKPREKKNTQEASE-------------AP--   218
```

```
Silurusm-XP_046727643-LOC124400094-SNew                      ------------QVEQE-DTGVVYAAVKLHKCSRDKTSQELRE-------------VP--   216
RedPiranha-XP_017539400-si:dkey-63d15.12-SNew                ------------AGGAEEPSSVLYAALNIRKPQDKRNSQEVSP-------------QPDV   210
MexicanTetra-XP_049342048-si:dkey-63d15.12-SNew              -----------QPAQREDDSSVLYAALKILKPEDRKNTQAIQQ-------------EL--   223

Bichir-XP_039627580-LOC120540683-SNew                        ----------------VAV---TSVKKSEERPEIVYSLITQ----------CKR------   259
Gar-XP_015221322-LOC107079700-Snew                           ARQARAGERHEEVRRRQEDRERRKKRSQKEGPRERLAHTPRRVASSRKRLDCLYISHQPL   279
Sterlet-XP_058888659-LOC117427588-Chr11-SNew                 -KESNYM-----------------------------------------------------   251
Sterlet-XP_058850216-LOC117973280-Chr21-SNew                 -KTPSEGSARIKHPKTQDA---IEQRGAEEEPEVLYAHVRG------------------   276
Xyrauchen-Texanus-XP_051965377-LOC127631324SNew              LA--------------------P-STGICSEDSVTYSEVHIKKGPKDEG----------   269
Cyprinus-carpio-XP_018965266-si:dkey-63d15.12-ChrA8-SNew     KA--------------------L-TSVTCTEDSVTYSEVHLKKGPKDEG----------   253
Sinocyclocheius-XP_016361154-NoLOC-SNew                      MA--------------------L-TSVTCTEDSVTYSEVHLKKGPKDEG----------   253
Danio-XP_017213004-si:dkey-63d15.12-SNew                     AA--------------------L-TSESCTEITVTYSEVHIKKRQKDE-----------   254
Icpu-XP_053536004-LOC108264924-SNew                          ------------------------DAGCEESPEVLYSDIRIKS----------------   264
Pangasius-XP_034162676-LOC113546007-SNew                     ------------------------DAGCAPNAEVLYSEIRIKS----------------   237
Silurusm-XP_046727643-LOC124400094-SNew                      ------------------------DAGCEGNPEVLYSDIHIKL----------------   235
RedPiranha-XP_017539400-si:dkey-63d15.12-SNew                GG--------------------PADAEGPTDSEVLYSDVRIKKK---------------   234
MexicanTetra-XP_049342048-si:dkey-63d15.12-SNew              ------------------------NQDCSTDNEVLYSGIRLKPQKQKHTTHSE-------   252
```

F - CD28x. Multiple alignment.

```
Galgal-XP_004938478-LOC101750509-S2                     ----MRSGILFMISYLVNI--TASSGPELLVLQYPEDASVLLNSTVWMLCVFEYPKEENE      54
Turtle-XP_025035993-LOC106731380-S2                     ---MMPLVIFIMISFFIQL--TVSSGAELSVVQYPQEINVSLDSTVWILCEFDYPEKAEV      55
Alligator-XP_014458214-LOC106737694-S2                  ---MMRLVILMTIPYCIHF--SVSSGSELSVIQYPQDINVSLDSTVLILCEFDYPAKTTV      55
Leueri_XP_055520789.1_LOC129715000_Chr45_486240-S2      MLALMRSLILMATISS-----LAGYEGTLSVRQHPGEVTSSVGRTVMVVCGAYYESQIPA      55
Heoc_XP_060682866_LOC132816884_Chr6_117049645-S2        MHPFKWSLPLMVACTC-----WAGDQSKVSVSQHPGEINVSVGMTAVIACEANYHRGTPE      55
SpottedGar-XP_015216968_LOC107079178_LG14-21903879-S2   ---MFHLLKLMFIPGIIGELRMVATDSQITARQHPQKITSFPGGSVAFICEFNYVVANPL      57
Sterlet-XP_033866345-LOC117406806-S2                    ---MFTSATLMFITGCIINSQIVTGGSKITVKQYPQEIRSSHGASVAITCEFNYQGKVPF      57

Galgal-XP_004938478-LOC101750509-S2                     EPVVYWRKGPSCDNQQSLRS-SS---GAGRPHIHIIKDIFRGFSILKLSSVDKNDSSSYF      110
Turtle-XP_025035993-LOC106731380-S2                     DTDVYWRKGPTCNQPG-LQS-STNTSHVQKSKVQIKTKESKRFSILKLDNVQMDDSGIYF      113
Alligator-XP_014458214-LOC106737694-S2                  DTEVYWRKGPDCNQPG-LKP-SPGISHFQKSQVQITKETTKRFSILKLNNVQLDDSNMYF      113
Leueri_XP_055520789.1_LOC129715000_Chr45_486240-S2      HVDVYWYKGEPSPQNALTN--LMESGSPRGGRLYLHGSHSNHYRILVISNLTLADAGVYT      113
Heoc_XP_060682866_LOC132816884_Chr6_117049645-S2        HTDMYWYKGNRRDQPILAFSKDSKSLSSQDDHLYVRGNLSKVYSILVINNLTVNDSGVYV      115
SpottedGar-XP_015216968_LOC107079178_LG14-21903879-S2   VIDVSWHHQSDVHQY--TFAKDNARLVQYSSNIHLHGNFSKGFSVLVIEDIQPNYTGTYF      115
Sterlet-XP_033866345-LOC117406806-S2                    QTDVSWFRSHNRTIN--HSSKNEKISTTDNLHIYVYGNHSKCFTILVIQDLQLNDTDKYF      115

Galgal-XP_004938478-LOC101750509-S2                     CDVILTQK--THGKCGKGTKLTVHDFI-CKDCIRSEPTWWCWFL--LLGYTLFVTTVIIA      165
Turtle-XP_025035993-LOC106731380-S2                     CDLNLSEPPPVRRKCGNGSKLTVHESK-CNSSIRHEKTSWVWFF--LLGYSICSSSILIF      170
Alligator-XP_014458214-LOC106737694-S2                  CDVTLTDPPPPQNKCGKGTKLTVHGPT-CHDS-RSDKNIWMWLL--LLGYSISATMIIIG      169
Leueri_XP_055520789.1_LOC129715000_Chr45_486240-S2      CEVSVPLPPPLVSQHGNGTRLHVEEATSCVALKTPTVMKQAIIIPALAIYSMVLTIAVMV      173
Heoc_XP_060682866_LOC132816884_Chr6_117049645-S2        CEVLVSLPPPHVSGYGHGTRLTVHESNSCQTSKAPQEVWKHTVFLAILAYSLAVTITTVS      175
SpottedGar-XP_015216968_LOC107079178_LG14-21903879-S2   CEVLLIQPPPV-KVTGKGTVLKVYDKEECKDQKSY---WWNYIFIALSATSCTLLFAVMF      171
Sterlet-XP_033866345-LOC117406806-S2                    CEVLLIVPPPI-TSRGNGTRLIVYDPEECQTQKCP---WLKYFFIALIACGGVVVAVLLM      171

Galgal-XP_004938478-LOC101750509-S2                     FGIHQCCKKCK---NDSRNTDSSTTLPSEWIYDKP-----SKPVNNGSNQEYEDMSLIRT      217
Turtle-XP_025035993-LOC106731380-S2                     FCILRCCKKYR---INSRNTDDLSTLPSEWTYDKP-----SRAVNNGFNQEYEDMTLMRN      222
Alligator-XP_014458214-LOC106737694-S2                  FLIHQCCKKFR---INSRTTDGSTTLPSEWTYDKP-----ARTVNNGFNQEYEDMTLIRS      221
Leueri_XP_055520789.1_LOC129715000_Chr45_486240-S2      LAVLLRFHMKSKGTLITKEISRGPEDPQPNTCRGHTRSLAGDRSRETHPQEYEDMTVFRT      233
Heoc_XP_060682866_LOC132816884_Chr6_117049645-S2        LGIFMCVHKRSKGRPNSKEALRSQEESSLTSSRKSALALSTDRAREAQNQEYEDMALIRT      235
SpottedGar-XP_015216968_LOC107079178_LG14-21903879-S2   MLVRKCL-----GRAKHITNKE------------QRKSEKKMEPIATISQEYEDMTFLRS      214
Sterlet-XP_033866345-LOC117406806-S2                    ICAKHCW-----GTVRNKMT---------------------CNPTDEKNTVYEDMTLVKS      205

Galgal-XP_004938478-LOC101750509-S2                     FSHIERKVI-----          226
Turtle-XP_025035993-LOC106731380-S2                     FTHNERKI------          230
Alligator-XP_014458214-LOC106737694-S2                  FTHNGHTMK-----          230
Leueri_XP_055520789.1_LOC129715000_Chr45_486240-S2      MNQR----------          237
Heoc_XP_060682866_LOC132816884_Chr6_117049645-S2        MSQIPR--------          241
SpottedGar-XP_015216968_LOC107079178_LG14-21903879-S2   QPAC----------          218
Sterlet-XP_033866345-LOC117406806-S2                    SQKGSQLPARKHAA          219
```

G - PD1. Multiple alignment.

```
Human-NP_005009-PD-1                          MQIPQAPWPVVWAVLQLGWRPGWFL----DSPDRP------WNPPTFSPALLVVTEGDNA   50
Gallus-XP_040534565-PD-1                      --MALGTSRTMWDSTEAALVVLCVLLLCCNPPLAG------CHQVTLFPATLTRPAGSSA   52
Pheasant-XP_031459296-PD-1                    --MALGTSRTMWDSMEGVLVVLCVLLLCCSPPLAG------CHQVTLFPAMLTRPVGSSA   52
ChineseSoftShelledTurtle-XP_014434034-PD-1    -----------------------------------------------------MPEGSNA   7
Anole-XP_062832999-PD-1                       --MESRHRIM-------HWVGAWLLLLCCRSALLL------KPSATFWPTQLNQSTGTTA   45
Xenoptrop-XP_031758711-PD-1                   ----MTHSPILCASLMSVERVFLIIIGICVITILGHARPEGVILFEHLPFEHHLTPGKTA   56
Gar-XP_015216859-LOC107079166                 --------------MSHPASRLSM-LSL---LLLLP----NANLQEEKMEAFRGTEGEHF   38
Sterlet-XP_033883562-NoLOC                    --------------MGKVTRVISAVTSL---LLLLI----QQGDSQPSELNLSSKEGQIL   39
Pike-XP_010894275-LOC105025357                --MHYCSCIMVCHIISKPLLQVVVIVQV-----LL-----N---GPSWAEEVVGNMGNNI   45
ElephantShark-XP_007897882-LOC103182617       ------------MILFIYQALSIFTVSLCSNVQD--KSPYIARQVKQHPASIEKKCGQSA   46
EpauletteShark-XP_060690415-LOC132821724      ---------------MNLSSLPWFVVGLG-----------ESLILKQYPMNIEKKSGQSA   34
                                                                                                          *

Human-NP_005009-PD-1                          TFTCSFSNTS---ESFVLNWYRMSPSNQTDKLAAFPEDRSQPGQDC--RFRVTQLPNGRD   105
Gallus-XP_040534565-PD-1                      TFICNISMEN-SSLEFNLNWYQKTNNSNPQKIAGIIRNIPQKKME-----KYRLFNNTPV   106
Pheasant-XP_031459296-PD-1                    TFICNISMEN-SSLEFNLNWYQKTNNSSPQKIAGIIRNIPQKKME-----KYRLFNDTPV   106
ChineseSoftShelledTurtle-XP_014434034-PD-1    SFFCNISTASFSRSDYSLNWYKKINSTHNQKIAELNGNEQQLQKE-----NFVLINHTST   62
Anole-XP_062832999-PD-1                       EFTCNISNAE--VFEDSVNWYKFDANKQPGKLDTRNKNKYEI-----------TRLDTQT   92
Xenoptrop-XP_031758711-PD-1                   VFICNISALNFN--PTDINWSKTHNNN-TSKIADIKSPKD----TN--RIHIETNWPSRI   107
Gar-XP_015216859-LOC107079166                 NMRFVFNSSIAPSSIKGAGLYKD-----GKKINEYI-KGGQ--SIGHHRILIKLEG--NN   88
Sterlet-XP_033883562-NoLOC                    TLNFTFPKKLSGTESMNLNLKKN-----QKKIAEINFMGGQGNKYNGKRLTFIWQGETQT   94
Pike-XP_010894275-LOC105025357                NISFSFPNTS-KAKVESVGLYKD-----GIKIGECNVNHVQNCSWK--KATWIFNMATTE   97
ElephantShark-XP_007897882-LOC103182617       VIHCILSNGSFS-EDLHLVWYRYIAKHQREKIGEINLKTQNMTAAN--NVQLLWNPDKLS   103
EpauletteShark-XP_060690415-LOC132821724      QIYCELHNGNAT-EDMRLVWYRYTSK---SKVGEINLRTRNFTAIN--RVLFKWDLSTYK   88
                                               :   :                :         *:

Human-NP_005009-PD-1                          FHMSVVRARRNDSGTYLCGAISLAPKAQIKESLRAELRVTERRAEVPT-----------A   154
Gallus-XP_040534565-PD-1                      FKMEILNLHQNDSGFYYCGLITFSRSDKVVESSHSQLVVTEAPEKTNT------IDEPSE   160
Pheasant-XP_031459296-PD-1                    FKMEILNLHQNDSGFYYCGLITFSRFDKVVESNHSQLVVTEASEKPNT------TEEPNE   160
ChineseSoftShelledTurtle-XP_014434034-PD-1    VEIKILNLTKSDSGHYYCGLIIFSSFSKVLESNVSQLTVTGILVSNES--SPSGTKDGTE   120
Anole-XP_062832999-PD-1                       FRMKILNLERNDSGVYSCILVATHSSLGFTESNHGNLTVTEIAPTPGS--PEDEYES-DH   149
Xenoptrop-XP_031758711-PD-1                   AELHIRNVTVNDSGEYHCEYLNVTANSKIMLSNRSRLNVTGDNEYAKT--FTE-------   158
Gar-XP_015216859-LOC107079166                 VTVRFNSLQLSDSGVYHIGLSRSITTDPLTLSSKVNLTVSERGN---------------   132
Sterlet-XP_033883562-NoLOC                    ATLSISGLIKNDSGVYQCNYVLN---SKIEVSNSINLTVRTEES-----SYTNQTDLSDP   146
Pike-XP_010894275-LOC105025357                AVFWIQKLTLADKGTYWATLFSNSRTFPIVKSNEVTLTLESDRITTESSSITNNIEAEDN   157
ElephantShark-XP_007897882-LOC103182617       AELRISHLKTSNSGSYGCELVSFNNKMNIKKDQPTNLTVTEEPAHP----GRENRTNSNA   159
EpauletteShark-XP_060690415-LOC132821724      ASMSITQLVKNDSGEYGCELVSFSSPEIIKKANATNLTVTEEQWIGPQ--NRNNTDNNKA   146

Human-NP_005009-PD-1                          HP-----SPSPRPAGQF--QTLVVGVVGG----LLGSLVLLVW---VLAVICSRAAR---   197
Gallus-XP_040534565-PD-1                      EE--------SSPPDHI--KAVLLGTLLL-----AGVIVLLLFG--YIIINNRRADV---   200
Pheasant-XP_031459296-PD-1                    EE--------SRPPDHI--KTVLLGTLLL-----AGVIVLLLFG--YIIINNRRADV---   200
```

```
ChineseSoftShelledTurtle-XP_014434034-PD-1      DE-----VEDNSWTGDF--NVPLIVIPSV-----AGAMLLGLIA--YMLS-CRMRGQ---   162
Anole-XP_062832999-PD-1                         DA-----KENENAGHNI--PLAAIGALGL-----VLVSVLASLCFFLIKVIRRRQER---   194
Xenoptrop-XP_031758711-PD-1                     ST-----TQSPMNKGTI--KLAVSISTSIFLILLLLSTSLLLWH-------KKRNKTPQT   204
Gar-XP_015216859-LOC107079166                   GTEAPPTPTQEDIPNQISYIIIASGATVLILLVVVGYC----------LYLATHKNT---   179
Sterlet-XP_033883562-NoLOC                      ASTADSHPQDHSIPQNP--VIAASVTVMALLLLVLSIT----------VFLIWDRNK---   191
Pike-XP_010894275-LOC105025357                  DG------TESNTNSTFTILIVIAVLGLPSVAMLLGLL----------IWFCWTEKR---   198
ElephantShark-XP_007897882-LOC103182617         EK-----PTGNMMRIEI--ILAAVISALLIVALLTYITVMYWPKKR-------------   198
EpauletteShark-XP_060690415-LOC132821724        EE-----FQNKEAR-----IQIIVAAVLAAFVIICLLIYLLFRYRPKKQ-----------   185


Human-NP_005009-PD-1                            --GTIG----------ARRTGQPL-KEDPSAVPVFSVDYGELDFQWREKTPEPP------   238
Gallus-XP_040534565-PD-1                        --QK------------PSSGNTLA-EVKPPVVPVPTVDYGVLEFQRDPHSQVPL------   239
Pheasant-XP_031459296-PD-1                      --RK------------PSSGNTLAEEVKPPVVSVPTVDYGVLEFQRDPRSQVPP------   240
ChineseSoftShelledTurtle-XP_014434034-PD-1      --QK------------PQSENAPLKDDQPPGITVYTVDYGVLEFQQEKLKAL-V------   201
Anole-XP_062832999-PD-1                         --GK------------PHDENAPL-EEEPPAVAVFTVDYGVLEFQARKNARRPP-FPPPS   238
Xenoptrop-XP_031758711-PD-1                     HLKHLVPSSSQSANECSTSPVQHIQEKPPQDPEVITVDYGVLAFPNNCPYRKSV------   258
Gar-XP_015216859-LOC107079166                   -----GPPAAL---TNPTPRTKIQDSEMSRGLPVYSIEYGVLEFPGAERRVEGE------   225
Sterlet-XP_033883562-NoLOC                      -----GTSS-----PQPTTIKRQQESKQSQAVPVYSIEYGVLEFPRRDTL-PPA------   234
Pike-XP_010894275-LOC105025357                  -----NPERDK---QEDFSAKPKESGEASTSMPVYSVEYGVVSFNNKPSGGKGAQHERAG   250
ElephantShark-XP_007897882-LOC103182617         -----IPI-------------------------LGYRYKMPDVRV------RIPF------ 217
EpauletteShark-XP_060690415-LOC132821724        -----GPN-----------------ANPPPA--DVSC------------QVIV------   202


Human-NP_005009-PD-1                            -VPCVPEQTEYATIVFPSGMGTSSPARRGSADGPRSAQPLRPEDGHCSWPL-------     288
Gallus-XP_040534565-PD-1                        -ETCPAEQTEYATIVFPEEKPIT--PERG-----------KRHKDERTWQLPSQPC--     281
Pheasant-XP_031459296-PD-1                      -ETCPAEQTEYATIVFPEEKPVT--PERG-----------KRHKDERTWQLPSQPC--     282
ChineseSoftShelledTurtle-XP_014434034-PD-1      -ESPTSDHTEYATIVFAEEKPVT--PERG-----------KRTKSPSTQ---AQPC--     240
Anole-XP_062832999-PD-1                         PKPQPLDQTEYATIIFPTEKSVP--MENTGKLGPMENT--KRTKRQVRWVPTGRQQLH     292
Xenoptrop-XP_031758711-PD-1                     -ELCTLDQVEYATIMFPQGTPSL--GERSGKDAACNRSPRVCRD-------------     299
Gar-XP_015216859-LOC107079166                   GQPVVQDNVEYATITFPPCQNPR--AGRGAQSQLCCGRVR-----------------     263
Sterlet-XP_033883562-NoLOC                      TMPEHNDHVEYATITFPAGQEGG--SG--GRHCWSHGR-------------------     268
Pike-XP_010894275-LOC105025357                  NIMRPSETVEYATITFPPRQRGA--NGR-----------------------------     276
ElephantShark-XP_007897882-LOC103182617         -PQCIPQTTLY----------------------------------------------     227
EpauletteShark-XP_060690415-LOC132821724        -LKVIDMH-------------------------------------------------     209
```
