# Supplementary material for: Extraordinary diversity of the CD28/CTLA4 family across jawed vertebrates
Source: Front Immunol. 2024 Nov 13;15:1501934. doi: 10.3389/fimmu.2024.1501934 (PMC11599192; doi:10.3389/fimmu.2024.1501934)
Supplement: Supplementary file 4 [file DataSheet4.pdf]

Supplementary file S4. Motifs and domains present in all protein models used in this study forCD28H, CD28HL1, CD28HL2, CD28HL3, CD28HL4, CD28X and PD-1.

Relevant motifs discussed in the text are in red.

	Species	CDR3 region	TM dimerization motif	Y based Signaling motif	Accession
CD28H	Human	YVCWAAVEIPELEEAEGNI	no conserved motif	YSNV	NP_001162597
	Oppossum	YECHMKKEIPLLLLEETNK	no conserved motif	YGNV	XP_007488972
	Tasmanian devil	YVCHVTMEIPELLLEETNK	no conserved motif	YGNV	XP_031803754
	Chicken	YVCHVTVEVPYLATAAGNG	no conserved motif	YVNI	XP_015155314
	Threetoed box turtle	YVCKAIVEIPVYLDATGNG	no conserved motif	YVNV	XP_029768145
	Alligator	YECSVNVEIPNLLSAVGNG	no conserved motif	YENV	XP_019350811
	Lungfish (Protopterus)	YVCEVNFEIPLLKQMKGGG	no conserved motif	YVNM	XP_043946518
	Sturgeon (A. ruthenus)	YFCRVILEIPALQTSCGNG	no conserved motif	YENM	XP_058869996
	Gar	YVCVVVMEIPSLAWHCSNG	no conserved motif	YENM	XP_015221026
	Eel	YFCEVVVEIPNFQQKCNPG	no conserved motif	YENM	XP_035271816
	Pike	YYCKISVEIPILKQNCSNG	no conserved motif	YTNM	XP_010870070
	Atlantic salmon	YFCHVSVEIPVLQQACSNG	no conserved motif	YTNM	XP_014072379
	Channel catfish	YFCQVTQDIPILISNLSNG	no conserved motif	YENT	XP_017333266
	Stickleback	YHCKIIAEIPVLDTTMSNT	no conserved motif	YANT	XP_040041332
	Little skate	YICQASIEIPPPVFTKYGNG	no conserved motif	YVNV	XP_055514805
	Epaulette shark	YVCEVLIEIPPPVYRMSGNG	no conserved motif	YVNV	XP_060702444
	Elephant shark	YVCEVFIEIPS-LHRDSGNG	no conserved motif		LOC103183582
CD28HI1	Human	absent		absent	
	Wombat	YLCKITVDIPIYKQAQGPG	no conserved motif	YEPM	XP_027692242
	Kiwi	YYCEIIIEIPFCERVYGNG	no conserved motif	YESF	XP_025910597
	Swan goose	YYCEIIIEIPFCKKLCGSG	no conserved motif	YESL	XP_013035024
	Alligator	YFCEINVEIP--YLKSGKG	no conserved motif	YDSL	XP_025050903
	Soft Shell Turtle	YFCEITIEIPFMKKGMGNG	no conserved motif	YESL	XP_025043104
	Clawed frog (X. tropicalis)	YICEVTIDKPELRFGKGTG	no conserved motif	YESC	XP_031751177
	Bichir	YYCILIYEIPILTRESGNG	no conserved motif	YRRR	XP_039607381
	Sterlet	YYCRTR-------RGQGGG	no conserved motif	YLTR	XP_058863796
	Gar	YSCKIIIEIPLLMMAQGLG	no conserved motif	YESF	XP_015200293
	European eel	YLCKITIEIP--IHKEGNG	no conserved motif	absent	XP_035282115
	Northern pike	YTCKVSSEIPVLQQSVGNG	no conserved motif	YESL	XP_010886301
	Rainbow trout	YICKVSLEIPSLIESVGDG	no conserved motif	YESL	XP_036814148
	Zebrafish??	YVCEVTQDIPVLEKKNGTG	no conserved motif	YWSY	XP_003200869
	Channel catfish	YICQVVQDIPRLVTVNGTG	no conserved motif	YESY	XP_017328639
	Stickleback	YICEVTVEIPNLNKAQGVG	no conserved motif	YVLR	XP_040035616
	Stickleback	YICEVTVEIPNLNKAQGNG	no conserved motif	YESF	XP_040036613
	Little skate	YVCQVTVEIPPP-IRKGSG	no conserved motif	YESF	XP_055499055
	Epaulette shark	YRCNMVIEIPAP-VRKGNG	no conserved motif	YESF	XP_060678526
	Elephant shark	YLCNVIVEIPILLKVNGNG	no conserved motif	YKRR	NP_001279065
CD28HI2	Human	absent		absent	
	Lungfish (Protopterus)	YYCVVTVEIPPPPKPHFGNG	no conserved motif	YKRE // -	XP_043918243
	Bichir	YQCEVRIEIPAP--LLKGKS	no conserved motif	YENQ // YINS	XP_039607697
	Mississippi paddlefish	YWCNVIVEIPLP--MQNGTG	no conserved motif	YVNN // YINS	XP_041124223
	Gar	YICKVIVEIPLF-LQQQGNG	no conserved motif	YMNS // YINS	XP_015200297
	European conger	YVCKIKVFIPVL-EQCDGNG	no conserved motif	YMDK // YINS	XP_061106870
	Bone fish (Albula goreensis)	YGCEVTVEIPTL-ASCYGTG	no conserved motif	YSNT // YINS	KAI1883834
	Epaulette shark	YHCKVFVEIPAPIRRASGEG	no conserved motif	YVNN // -	XP_041039503
	Great white shark	YHCEVLVEIPPPVHRAIGEG		YENS // -	XP_060678526
CD28HI3	Human	absent		absent	
	Bichir	YICEVTRWIPSPLIRRSGNG	no conserved motif	YAEV-x(40)-IVYSLI	XP_039627580
	Sterlet	YACKITPLIPVQLEGAWGNG	no conserved motif	FGYSVL-	XP_015221322
	Gar	YACKITPLIPVQLEGAWGNG	no conserved motif	VCYASL-x(61)-VLYAHV	XP_058850216
	Xyrauchen texanus	YYCKVWSDLP-KLFEAYGEG	no conserved motif	IVYAAL-x(30)-VTYSEV	XP_051965377
	Common carp	YYCTAQVDLLMLGPVEHGNG	no conserved motif	VVYTAL-x(28)-VTYSEV	XP_018965266
	Sinocyclocheius	YYCTAFVDLPTLEPAELGNG	no conserved motif	VVYTAL-x(28)-VTYSEV	XP_016361154
	Zebrafish	YYCSAQLDLPELGPVEYGKG	no conserved motif	VIYTAL-x(28)-VTYSEV	XP_017213004
	Channel catfish	YYCKVWQDVPRLRSETVGGG	no conserved motif	VVYAAV-x(27)-VLYSDI	XP_053536009
	Silurus	YYCTVLKHIPKLVQFTDTTG	no conserved motif	VVYAAV-x(27)-VLYSDI	XP_046727643
	Pangasius	YYCTVWQDVPHLMSGADSTG	no conserved motif	VVYAAV-x(27)-VLYSEI	XP_034162676
	Red belly piranha	YYCAVFQELPMLGEKVYGPG	no conserved motif	VLYAAL-x(33)-VLYSDV	XP_017539400
	Mexican tetra	YYCTVWQDVPHLMSGADSTG	no conserved motif	VLYAAL-x(27)-VLYSGI	XP_034162676
D28X (S	Human	absent		absent	
	Chicken	YFCDVILTQK--THGKCGKG	YTLFVT	YEDM	XP_004938478
	Turtle	YFCDLNLSEPPPVRRKCGNG	YSICSS	YEDM	XP_025035993
	Alligator	YFCDVTLTDPPPPQNKCGKG	YSISAT	YEDM	XP_014458214
	Gar	YFCEVLLIQPPP-VKVTGKG	no motif	YEDM	XP_015216968
	Little skate	YTCEVSVPLPPPLVSQHGNG	YSMVLT	YEDM	XP_055520789
	Epaulette shark	YVCEVLVSLPPPHVSGYGHG	YSLAVT	YEDM	XP_060682866
PD1	Human	YLCGAISLAPKAQIKESLRAELRV	no conserved motif	VDYGVL-x(19)-TEYATI	NP_005009
	Pheasant	YYCGLITFSRFDKVVESNHSQLVV	no conserved motif	VDYGEL-x(19)-TEYATI	XP_040534565
	Chinese Soft Shelled Turtle	YYCGLIIFSSFSKVLESNVSQLTV	no conserved motif	VDYGEL-x(18)-TEYATI	XP_014434034
	Anole	YSCILVATHSSLGFTESNHGNLTV	no conserved motif	VDYGEL-x(25)-TEYATI	XP_062832999
	Clawed frog (X. tropicalis)	YHCEYLNVTANSKIMLSNRSRLNV	no conserved motif	VDYGEL-x(19)-VEYATI	XP_031758711
	Gar	YHIGLSRSITTDPLTLSSKVNLTV	no conserved motif	IEYGEL-x(20)-VEYATI	XP_015216859
	Sterlet	YQCNYVLN---SKIEVSNSINLTV	no conserved motif	IEYGEL-x(19)-VEYATI	XP_033883562
	Pike	YWATLFSNSRTFPIVKSNEVTLTL	no conserved motif	VEYGEL-x(25)-VEYATI	XP_010870070
	EpauletteShark	YGCELVSFSSPEIIKKANATNLTV	no conserved motif	no conserved motif	XP_060690415
	Elephant Shark	YGCELVSFNNKMNIKKDQPTNLTV	no conserved motif	no conserved motif	XP_007897882
