# Supplementary material for: Extraordinary diversity of the CD28/CTLA4 family across jawed vertebrates
Source: Front Immunol. 2024 Nov 13;15:1501934. doi: 10.3389/fimmu.2024.1501934 (PMC11599192; doi:10.3389/fimmu.2024.1501934)

Supplementary File S7. Conserved synteny blocks with CD28HL3 (white). Only conserved markers are represented in the regions of interest, of which start and end location are indicated in the reference NCBI chromosome/LG of each species..

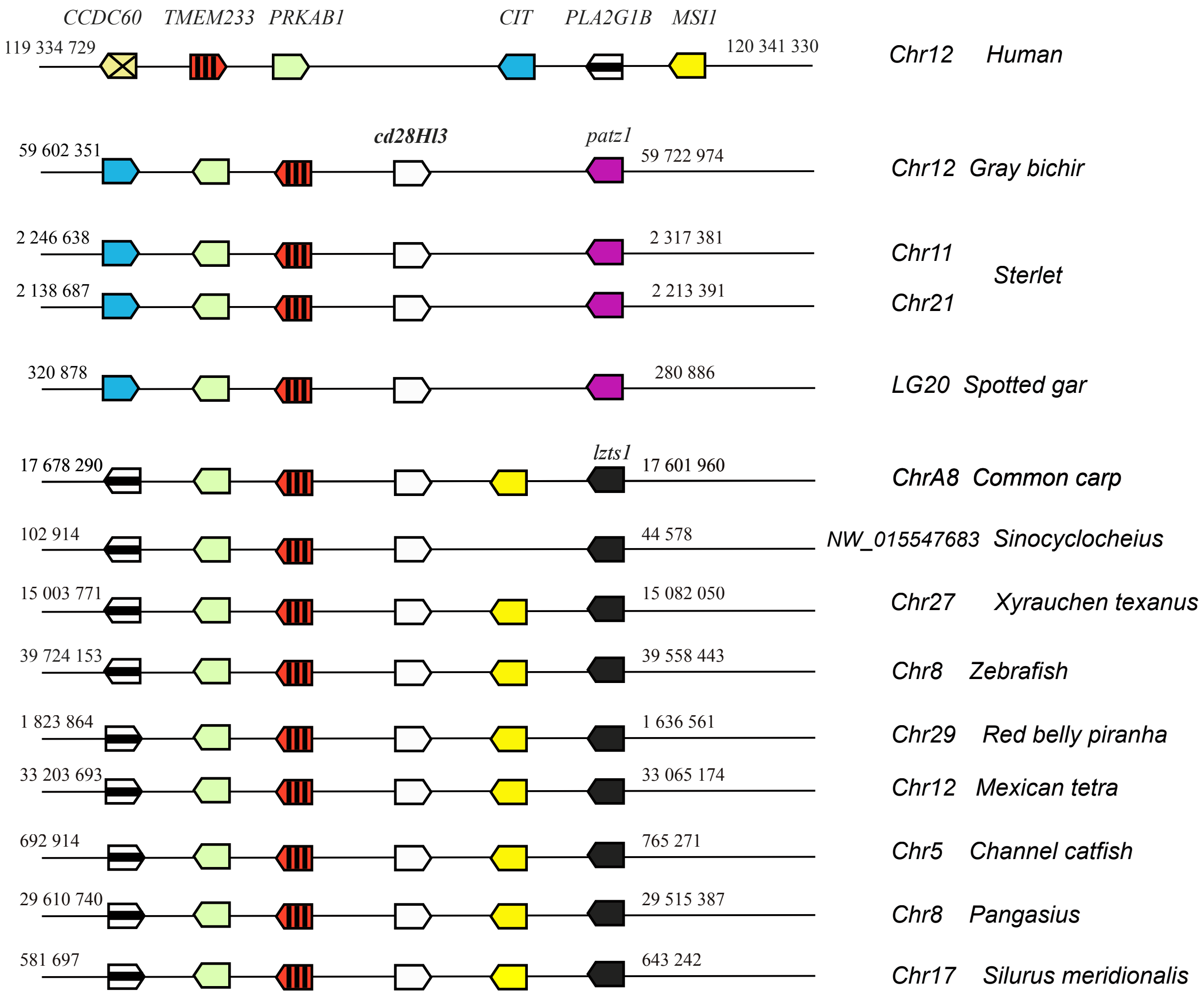

Supplement: Supplementary file 7 [file DataSheet7.pdf]
