# Supplementary material for: Extraordinary diversity of the CD28/CTLA4 family across jawed vertebrates
Source: Front Immunol. 2024 Nov 13;15:1501934. doi: 10.3389/fimmu.2024.1501934 (PMC11599192; doi:10.3389/fimmu.2024.1501934)

Supplementary File S8. Conserved synteny blocks with CD28x (white X on brown background). Only conserved markers are represented in the regions of interest, of which start and end location are indicated in the reference NCBI chromosome/LG of each species.

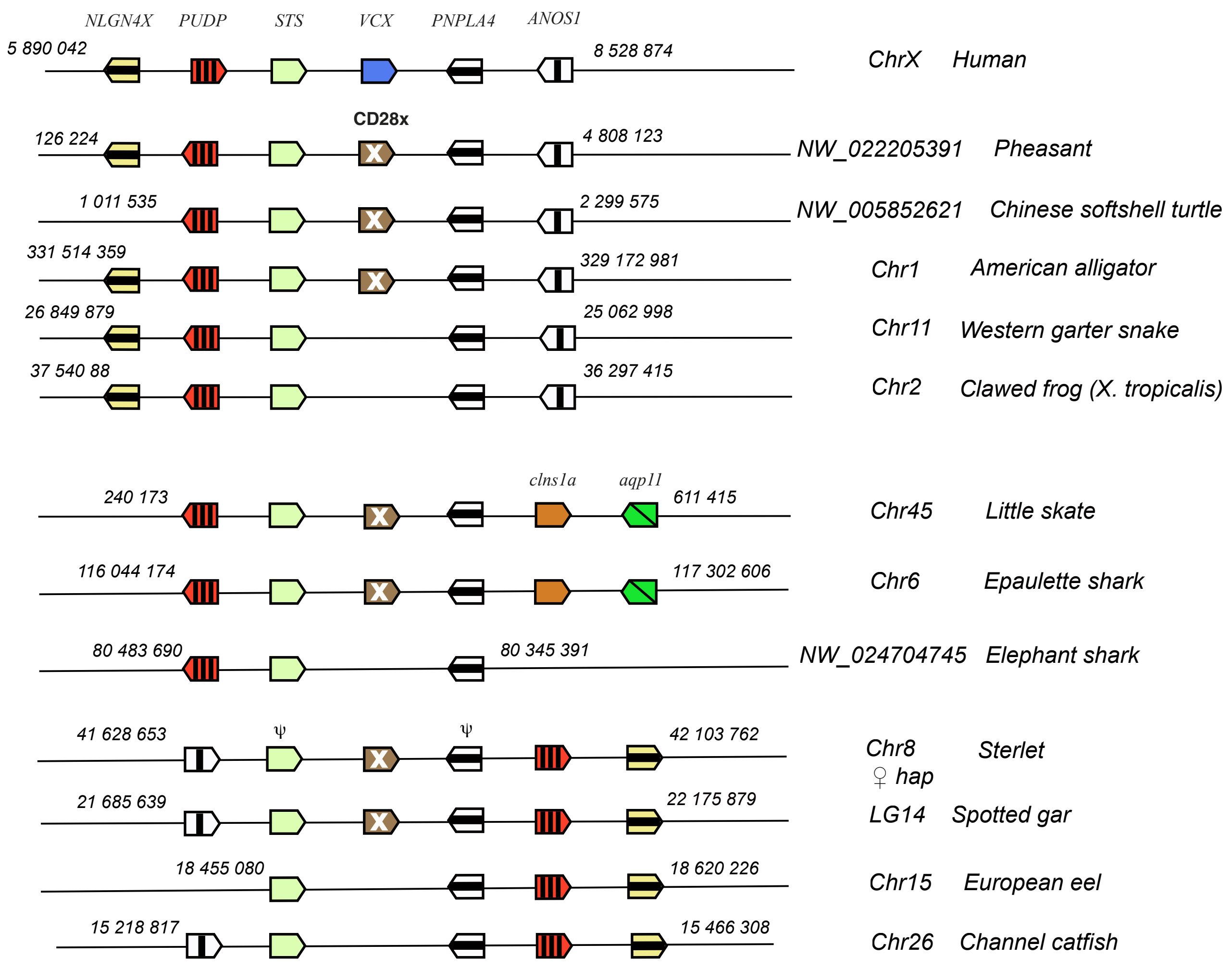

Supplement: Supplementary file 8 [file DataSheet8.pdf]
