# Supplementary material for: Extraordinary diversity of the CD28/CTLA4 family across jawed vertebrates
Source: Front Immunol. 2024 Nov 13;15:1501934. doi: 10.3389/fimmu.2024.1501934 (PMC11599192; doi:10.3389/fimmu.2024.1501934)
Supplement: Supplementary file 9 [file DataSheet9.pdf]

Supplementary file S9. CD28 and closely related genes are not found in Agnathans, but the checkpoint receptor IgSF-11 is conserved across vertebrates.

We then looked for IgSF members similar to CD28 or related sequences in lamprey and hagfish databases, but no convincing orthologous gene could be found using blast search or synteny based locus characterization. The lack of hagfish or lamprey protein model containing Interpro motifs IPR008093 (CD28) or IPR040216 (CD28/CTLA4) or IPR039695 (present in TMIGD2 aka CD28H) also supported these observations. Similarly, no ortholog of CD28 or related genes could be found in lamprey OrthoDB. In contrast, homologs of genes encoding the immune checkpoint IgSF-11/VSGIG3 (LOC116948162) and also A33 (LOC116948206), belonging to the CTX family (1), were present in this species. In human, while A33 was not clearly linked to immune regulatory functions, Ig-SF11 was identified as the ligand of VISTA, a member of the B7 family (2), and constitutes an immune checkpoint of growing importance in tumor immunology (3, 4). This membrane protein with 2 IgSF domains inhibits T-cell responses and promotes immune-suppressive microenvironment in tumors. Interestingly, cross-linking of frog CTX inhibits tumor growth, indicating that this connection is evolutionary conserved (5). Mouse IgSF-11 controls osteoclast differentiation through modulation of PKM2-mediated glucose metabolism, a mechanism requiring interaction with the adaptor PSD-95 via 75 C-terminal amino acids of IgSF1 (6, 7). Interestingly, the Cter sequences of human and lamprey IgSF-11 are highly conserved (see below), suggesting this signaling mechanism might be shared.

```
Alignment of human and lamprey IgSF-11.

human        ----MTSQRSPLAPLLLLSLH----GVAASLEVSESPGSIQVARGQPAVLPCTFTTSAA-      51
lamprey      MDREMHLSGEEL-LLLLLAIAGACIGGARGVVVTVNPAEVTVARGQAAELPCSVSSNAPT      59
                 *  . . *  ****::     * * .: *: .*..: ***** * ***:.::.*

human        LINLNVIWMVTPLSNANQPEQVILYQGGQMFDGAPRFHGRVGFTGTMPATNVSIFINNTQ      111
lamprey      LNNLVVLWIANTE--RGAGEQVLGFMAGQVVNGSASFAGRADFTSPMPGSAVSVRVERAR      117
             * ** *:*:..     .  ***: : .**:.:*:  * **..**. **.: **: ::.::

human        LSDTGTYQCLVNNLPDIGGRNIGVTGLTVLVPPSAPHCQIQGSQDIGSDVILLCSSEEGI      171
lamprey      ESDAGAYTCSVNLIADPPG-GVGVVTLTVLVPPATPRCIMEGDPTVGTNITLTCSSSEGN      176
              **:*:* * ** : *  * .:**. *******::*:* ::*.  :*::: * ***.**

human        PRPTYLWEKLDNTLKLPPTATQDQVQGTVTIRNISALSSGLYQCVASNAIGTSTCLLDLQ      231
lamprey      PAPSYLWTRLEAARKLPANAALGRKEGTLTLTNVSADSEGVYSCTARNSVGSSNCTITLA      236
             * *:*** :*: : *** .*: .: :**:*: *:** *.*:*.*.* *::*:*.* : *

human        VISPQPRNIGLIAGAIGTGAVIIIFCIALILGAFFYWRSKNKEEEEEEIPNEIREDDLPP      291
lamprey      LRIPPSVNVGLVAGVLVGSLLGV--ALVLLLLGYAWARRRKATGKEDELPNDIRMDAPPP      294
             :  *   *:**:**.:  . : :  .:.*:* .: : * ::   :*:*:**:** *  **

human        KCSSAKAFHTEISSSDN--NTLTSS--NAYNSR---YWSNNPKVHRNTESVS--------      336
lamprey      QYAASGSKTGSTPRPGSGLSSLSSAPGPPYKHPANGAAAHGPTTHRAGGSDRHHHPHHHH      354
             : ::: :   .    ..  .:*:*:    *:       ::.*..**   *

human        -----------------H---FSDLGQSFSFHSGNANIPSIYANG-THLVPGQHKT----      371
lamprey      PHHLHQPHHAHHQHNASHVAGANGHGRAPAFAVVDAGAPSVVTASLVNPAPGSGRQNAYG      414
                              *    .. *:: :*   :*. **: : . .: .**. :

human        ----------------------LVVTANRGSSPQVMS-RSNGSVSRKPRPPHTHSYTISH      408
lamprey      QNPQPSRCDEAGVPFEPLANQTSPLSPSNGPTPSPIPMTSPGPMDFGVGVSPVPARAVSP      474
                                     :: ..* :*. :   * * :.       . : ::*
human        ATLERIGAVPVMVPAQSRAGSLV   431
lamprey      GNLVRMGGVPIMVPAQSRAGSLV   497
             ..* *:*.**:************
```


References :

1. Chretien, I., J. Robert, A. Marcuz, J. A. Garcia-Sanz, M. Courtet, and L. Du Pasquier. 1996. CTX, a novel molecule specifically expressed on the surface of cortical thymocytes in Xenopus. *Eur J Immunol* 26: 780-791.
2. Mehta, N., S. Maddineni, Mathews, II, R. Andres Parra Sperberg, P. S. Huang, and J. R. Cochran. 2019. Structure and Functional Binding Epitope of V-domain Ig Suppressor of T Cell Activation. *Cell Rep* 28: 2509-2516 e2505.
3. Ghouzlani, A., S. Rafii, M. Karkouri, A. Lakhdar, and A. Badou. 2020. The Promising IgSF11 Immune Checkpoint Is Highly Expressed in Advanced Human Gliomas and Associates to Poor Prognosis. *Front Oncol* 10: 608609.
4. Xie, X., C. Chen, W. Chen, J. Jiang, L. Wang, T. Li, H. Sun, and J. Liu. 2021. Structural Basis of VSIG3: The Ligand for VISTA. *Front Immunol* 12: 625808.
5. Robert, J., I. Chretien, C. Guiet, and L. Du Pasquier. 1997. Cross-linking CTX, a novel thymocyte-specific molecule, inhibits the growth of lymphoid tumor cells in Xenopus. *Mol Immunol* 34: 133-143.
6. Kim, H., N. Takegahara, and Y. Choi. 2023. IgSF11-mediated phosphorylation of pyruvate kinase M2 regulates osteoclast differentiation and prevents pathological bone loss. *Bone Res* 11: 17.
7. Kim, H., N. Takegahara, M. C. Walsh, S. A. Middleton, J. Yu, J. Shirakawa, J. Ueda, Y. Fujihara, M. Ikawa, M. Ishii, J. Kim, and Y. Choi. 2020. IgSF11 regulates osteoclast differentiation through association with the scaffold protein PSD-95. *Bone Res* 8: 5
